# Multi-stage single-cell atlas of a chordate non-embryonic development unveils dedifferentiating bud founder cells

**DOI:** 10.1038/s41467-026-76441-3

**Published:** 2026-09-03

**Authors:** Marie Lebel, Solène Marchal, Tiphaine Sancerni, Sharon Rabiteau, Pragati Sharma, Tal D. Scully, Estelle Balissat, Laurel S. Hiebert, Anthony W. De Tomaso, Allon M. Klein, Alexandre Alié, Stefano Tiozzo

**Affiliations:** 1 Laboratoire de Biologie du Développement de Villefranche-sur-Mer (LBDV), UMR7009, CNRS, Sorbonne Université, 06230 Villefranche-sur-Mer, France; 2 Department of Systems Biology, Harvard Medical School, Boston, MA USA; 3 Molecular, Cellular and Developmental Biology, University of California, Santa Barbara, CA USA

**Keywords:** Developmental biology, Regeneration, Evolutionary developmental biology

## Abstract

Colonial tunicates are the only chordates capable of forming fully functional bodies from somatic tissues through non-embryonic development, or budding. In *Botryllus schlosseri*, budding produces new individuals (zooids) from a portion of the peribranchial epithelium of a preexisting zooid. While the anatomy of this process has been described in detail, the cellular and molecular mechanisms underpinning bud initiation remain elusive. Here, we present a multi-stage single-cell transcriptomic atlas of a non-embryonic development in a chordate, capturing initiation and development of zooids. We identify an epithelial founder cell population that gains developmental potential through possible dedifferentiation, challenging the long-standing hypothesis that a long-lived, circulating pluripotent stem cell drives new zooid formation. Although some circulating cells exhibit stem-like expression profiles, these populations are either hematopoietic or germline restricted lineages. This atlas provides a foundational resource for understanding the cellular origin and mechanisms underlying agametic development and possibly whole-body regeneration in chordates.

## Abstract (web summary)

The cellular origins of non-embryonic body formation in chordates remain poorly understood. Here, using single-cell transcriptomics across seven stages of asexual development, the authors identify epithelial founder cells that acquire developmental potential through dedifferentiation, providing a cellular framework for non-embryonic body formation in a chordate.

## Introduction

Colonial tunicates are the only chordates capable of developing a fully functional body from somatic cells, either through cyclical agametic propagation or in response to extensive injury via whole-body regeneration. These forms of non-embryonic development are collectively referred to as budding^1^. When part of the agametic life cycle, budding produces new clonal individuals that generally remain physically connected to the parent, ultimately forming colonies composed of up to hundreds of genetically identical zooids.

**Fig. 1:**
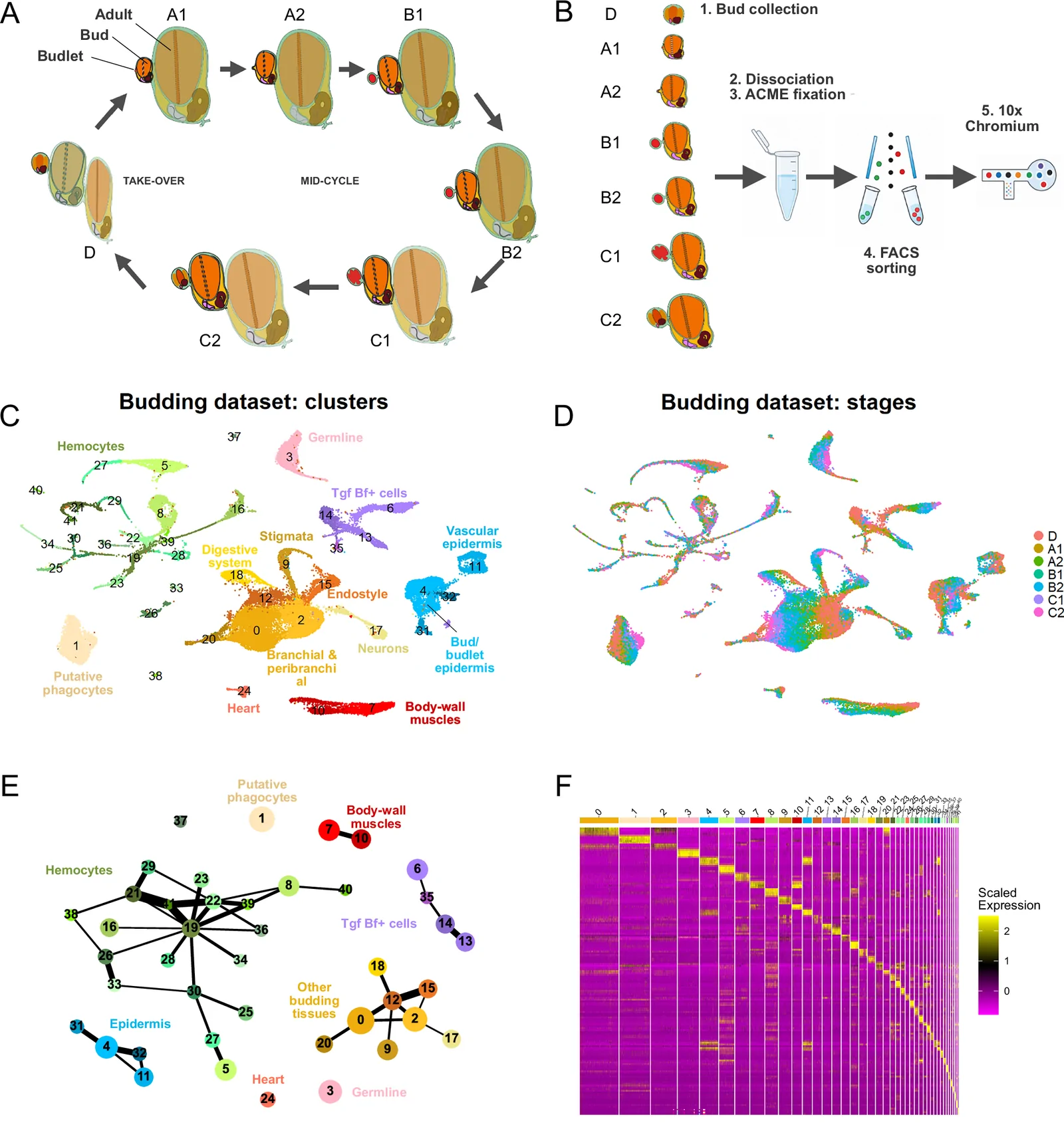
A single-cell atlas of bud development in Botryllus schlosseri. **A** Schematic representation of *B. schlosseri* peribranchial budding cycle (modified from^81^), with developmental staging based on^8^. **B** Overview of the experimental workflow. **C** UMAP projection of the full budding dataset showing the 42 clusters. **D** Same UMAP as in (**C**), with cells colored by developmental stage, illustrating the temporal distribution across clusters. **E** Partition-based graph abstraction (PAGA) of the full dataset (threshold = 0.155), revealing nine major cell groups based on transcriptional connectivity. **F** Heatmap showing the differentially expressed genes per cluster, highlighting the transcriptomic signatures defining each cell population, color represents scaled expression level of the marker genes.

Tunicate phylogeny suggests that budding is an evolutionarily labile trait, having arisen independently multiple times^1,2^. In sharp contrast to the highly conserved embryonic development observed across major tunicate orders, and despite a broadly conserved zooid body-plan, budding displays remarkable diversity: it can originate from different, often non-homologous cell types and tissues; proceed through distinct developmental trajectories; and involve variable interactions between epithelial and mesenchymal cells, sometimes even within a single species^1^. While the morphological diversity of tunicate budding has been recorded in scattered literature for over two centuries (reviewed in ref. ^1^), much of the current understanding stems from more recent investigations in a few model species, particularly within the Styelidae family^1,2^. Among these, *Botryllus schlosseri* has emerged as the leading laboratory model over the past decades, not only for the study of budding^3,4^, but also for investigating biological processes such as allorecognition and chimerism^5,6^. In *B. schlosseri*, agametic propagation occurs via a highly stereotyped budding mode known as palleal or peribranchial budding^4,7^. Peribranchial budding is a continuous, lifelong process that, unlike sexual reproduction, bypasses embryonic stages and metamorphosis, and enables the colony to grow and propagate. During this process, buds arise from specific regions of the peribranchial epithelium of the parental zooid and progress through stereotyped morphogenetic stages (Fig. 1A; see Methods^7,8^). At a given time, a colony harbors three synchronized asexual generations: the adult zooids, which are feeding and sexually reproducing; primary buds, which are completing organogenesis and growth; and the secondary buds (or budlets), which arise from the epithelia of the primary bud and are in the initial phase of development. Colonies periodically undergo a synchronized apoptotic clearance of the adults (take-over) that are replaced by their mature primary buds (phase D, Fig. 1A^7^).

The anatomy and morphological stages of budding in *Botryllus schlosseri* have been well described^7^, and several studies have investigated the molecular mechanisms underlying regulation of early cell commitment^9–11^, establishment of developmental axes and symmetry^12–14^, gametogenesis^15,16^, vasculo- and angiogenesis^17–19^ and the differentiation of specific tissues such as muscle and nervous lineages^20,21^. Parallel to these findings, various hypotheses have emerged regarding the identity and nature of the cells that initiate the budding process. One long-standing hypothesis suggests that the development of asexually derived bodies originates from a pool of stem cells functionally analogous to embryonic stem cells^22^. Supporting this view, studies have shown that circulating mesenchymal cells can engraft and contribute long-term to the gonads and to certain uncharacterized somatic tissues^23^. In line with this, Voskoboynik and colleagues used in vivo cell labeling, engraftment, and time-lapse imaging to identify a population of stem-like cells located antero-ventral to the endostyle^24^. The same group also described discrete structures known as “cell islands,” proposed to act as niches for *Piwi*-positive cells that may contribute to both germline and somatic lineages during budding^25^. However, direct evidence for broad somatic contributions from circulating progenitors remains limited. Notably, a longitudinal study following the source of budding of endodermal tissues in chimeric colonies for over ten blastogenic cycles found no evidence of a mobile precursor^26^. Studies in closely related species, such as *Botrylloides violaceus* and *Botrylloides diegensis*, have reported the presence of circulating undifferentiated cells, yet involved in injury-triggered whole-body regeneration, a phenomenon referred to as vascular budding^27,28^. An alternative hypothesis proposes that in colonial tunicates, the remarkable flexibility of tissue remodeling observed in whole-body regeneration and agametic reproduction is driven by two complementary mechanisms: dedifferentiation of epithelial cells and the epithelial transformation of circulating cells, thereby bypassing the need for activation of multi- or pluripotent stem cells (reviewed in ref. ^29^).

Despite detailed knowledge of bud morphology and early tissue patterning in *B. schlosseri*, the identity of bud founder cells and the molecular mechanisms initiating peribranchial budding—particularly how committed tissues regain developmental competence—remain unknown.

In this study, we present a multi-stage single-cell transcriptomic (scRNAseq) atlas for tunicate agametic development, capturing the progression of peribranchial budding in *Botryllus schlosseri*, from the earliest pre-budlet stage to the formation of a nearly mature zooid. This dataset enabled us to pinpoint the epithelial origin of budding cells by identifying and characterizing the transcriptomic signature of the founder population within the peribranchial epithelium. Beyond characterizing this key lineage, the high-resolution atlas provides a valuable resource for investigating the cellular and molecular mechanisms driving non-embryonic development in chordates. By providing a detailed map of cells engaged in budding, along with robust gene expression profiles to enable accurate cluster annotation, this work lays the foundation for re-evaluating long-standing developmental hypotheses in a comprehensive transcriptomic and genomic context.

## Results

### A multi-stage atlas of B. schlosseri budding

To investigate the molecular and cellular changes during *B. schlosseri* asexual development via peribranchial budding^7^, we collected buds and their associated budlets from phase A1 to phase C2, as well as budlets at phase D (Fig. 1A, B, Supplementary Fig. 1A), all derived from an isogenic colony (*i.e*. originating from a single founder larva). Following dissociation, single cells were sorted by FACS and loaded onto 10X Chromium V3 chips (Supplementary Fig. 1B, Supplementary Table 1). Cell viability and RNA quality were confirmed prior to processing (Supplementary Fig. 1A, C). Sequenced reads were mapped on the latest version of *B. schlosseri* genome^30^, with mapping metrics confirming overall high data quality (Supplementary Table 2). CellRanger initially retained 78,257 cells across all samples; after filtering out low-quality cells and doublets using soft thresholds (Supplementary Figs. 2, 3), the final budding dataset comprised 66,227 cells. All samples were preprocessed and analyzed jointly.

Clustering identified 42 transcriptionally distinct clusters, visualized using Uniform Manifold Approximation and Projection (UMAP, Fig. 1C). Quality metrics across the full dataset and within clusters are shown in Supplementary Fig. 4. Each cluster contains cells from multiple budding stages (Fig. 1C, D, Supplementary Fig. 5A) and most display a clear developmental gradient along the UMAP (Fig. 1D), consistent with the progressive morphogenesis of buds and budlets (Fig. 1A, B), and indicative of potential differentiation trajectories within the dataset. Importantly, we chose not to integrate the samples, as preserving stage-specific biological variation was critical to our analyses. In support to this approach, the two biological replicates for phase B2 showed strong overlap, indicating minimal batch effect and confirming that integration was unnecessary (Supplementary Fig. 5B).

To explore potential differentiation trajectories, we applied CytoTRACE and RNA velocity analyses. While CytoTRACE is generally considered less reliable in datasets comprising multiple heterogeneous tissues^31,32^, its application to our full dataset revealed patterns consistent with progressive cellular differentiation, in line with the developmental staging (Supplementary Fig. 6A–C). RNA velocity analysis was limited by the low proportion of unspliced transcripts (approximately 5% calculated by Velocyto^33^). Nevertheless, velocity vectors projected onto the UMAP broadly aligned with the gradients identified by both CytoTRACE and stage-based progression, with some local deviations (Supplementary Fig. 6D).

To further explore relationships between clusters, we applied PAGA (Partition-based Graph Abstraction^34^), which revealed nine major transcriptional groups (Fig. 1E, Supplementary Fig. 6E). Differential expression analysis identified robust marker genes for each cluster (Fig. 1F), enabling cell-type annotation and downstream pathway analyses.

Cluster 7 and 10 express *Myh3* (*Boschl.Bs15.g19623-Myh6*) a marker of body wall muscles, and *Ebf* (*Boschl.Bs10.g14018-EBF1*), which is specific of developing muscles and nervous system (Supplementary Table 3, Supplementary Fig. 7A^21^). These clusters also express myogenic markers such as *Mrf (Boschl.Bs4.g5757-MYOD1)*^21^, *Troponin-like* (*Boschl.Bs10.g14218-TNNT2.1*) and a *Titin-like (Boschl.Bs5.g6251)*^35^, consistent with muscle lineage identity. Several gene ontology (GO) terms associated with muscle identity are highly enriched in this cluster, including “skeletal myofibril assembly” or “sarcomere organization” (Supplementary Fig. 7B).

Cluster 24 was annotated as heart cells based on the expression of *Myh2 (Boschl.Bs8.g10861-MYH3.1)*. This cluster also expresses transcription factors known to be expressed during heart development, including *Gata-a, Tbx1/10, FoxF* and *Nk4* (Supplementary Table 3, Supplementary Fig. 7A^9,21^). Similarly, GO terms associated with muscular identity, and in particular with cardiac muscle, are enriched in this cluster (Supplementary Fig. 7B).

Cluster 3 was identified as germline cells based on the strong expression of *Vasa (Boschl.Bs4.g5801-DDX4)*, a well-established germline marker, as well as *Piwi1 (Boschl.Bs5.g6728-PIWIL1.1)* (Supplementary Table 3, Supplementary Fig. 7A^36^), along with the expression of multiple genes from the germline multipotency program (GMP) genes (below^37^). Accordingly, several GO terms associated with meiosis are highly enriched in this cluster (Supplementary Fig. 7B).

Clusters 6, 13 and 14 showed enriched expression of *Tgf-β-f (Boschl.Bs8.g10511)*, which has been described in follicular and germline support cells (Supplementary Table 3, Supplementary Fig. 7A^36^). Given the heterogeneity and transcriptomic complexity of these clusters, they were conservatively annotated as *Tgf-β-f+* cells.

To resolve the identity of previously unannotated clusters, we performed whole-mount fluorescent in situ hybridization and we successfully adapted a hybridization chain reaction (HCR) protocol for *B. schlosseri* (see Methods). For reliable cell-type annotation, we defined marker genes as those showing both strong differential expression and high specificity within a given cluster. To reduce the risk of misassignment, we validated at least two marker genes per cluster whenever possible.

**Fig. 2:**
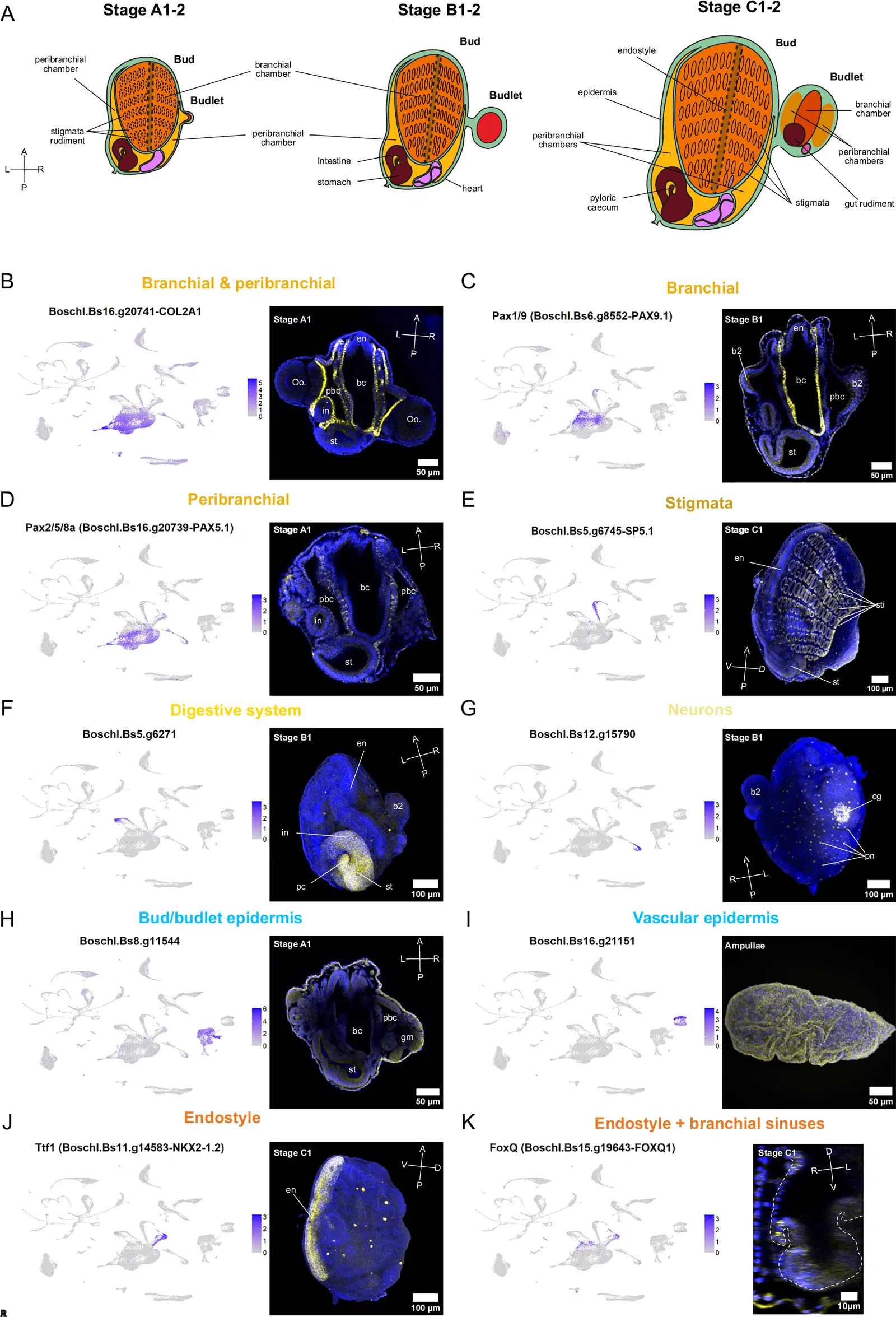
Annotation of the budding tissues clusters based on gene expression patterns. **A** Scheme of the main budding stages (dorsal view). **B–K** Left panels: expression plots of selected marker genes across the budding dataset, colors represent normalized expression of the gene. Right panels: corresponding whole-mount in situ hybridization (WMISH) or hybridization chain reaction (HCR) confocal images. Probe signal is shown in yellow; Hoechst nuclear staining in blue. **B** *Boschl.Bs16.g20741-COL2A1*, stage A1 bud. **C** *Pax1/9 (Boschl.Bs6.g8552-PAX9.1)*, stage B1 bud. **D** *Pax2/5/8a (Boschl.Bs16.g20739-PAX5.1)*, stage A1 bud. **E** *Boschl.Bs5.g6745-SP5.1*, stage C1 bud. **F** *Boschl.Bs5.g6271*, stage B1 bud. **G** *Boschl.Bs12.g15790*, stage B1 bud. **H** *Boschl.Bs8.g11544*, stage A1 bud. **I** *Boschl.Bs16.g21151*, epidermis of ampulla. **J** *Ttf1 (Boschl.Bs11.g14583-NKX2-1.2)*, stage C1 bud. **K** *FoxQ* (*Boschl.Bs15.g19643-FOXQ1*), stage C1 bud endostyle (outlined with dotted line). Color code matches Fig. 1C, E. en endostyle, oo oocyte, bc branchial chamber, pbc peribranchial chamber, in intestine, st stomach, b2 budlet, sti stigmata, pc pyloric cecum, cg cerebral ganglion, pn peripheral neurons, gm gonadal mass. All presented patterns of expression have been observed in at least 3 samples of the same stage.

Clusters 0, 2, and 20 were annotated as the branchial and peribranchial epithelia (excluding the endostyle and stigmata), based on the expression of the collagen *Boschl.Bs16.g20741-COL2A1* (Fig. 2B), as well as *Pax1/9 (Boschl.Bs6.g8552-PAX9.1*, Fig. 2C) or *Pax2/5/8a (Boschl.Bs16.g20739-PAX5.1*, Fig. 2D) for the branchial and peribranchial epithelia respectively. These clusters also express transcription factors known to be expressed in these tissues, such as *Gata-b*, *Tbx1/10*, *Tbx2/3* and *Nk4* (Supplementary Table 3, Supplementary Fig. 7A^9,21^). Notably, Tbx1/10 and Nk4 are also components of the conserved cardio-pharyngeal muscle gene regulatory network^38^. However, as these factors are not heart-specific in ascidians^9,21,38^ and are also involved in pharyngeal development, their expression is consistent with the assigned branchial/pharyngeal identity, although a broader cardio-pharyngeal potential cannot be excluded.

Cluster 9 was identified as the stigmata, characterized by the expression of *Boschl.Bs5.g6745-SP5.1* (Fig. 2E) and *Boschl.Bs15.g20397* (Supplementary Fig. 8), as well as *FoxA2* (Supplementary Table 3, Supplementary Fig. 7A^9^).

Cluster 18 corresponds to the digestive system, as shown by the expression of the marker genes *Boschl.Bs5.g6271* (Fig. 2F) and the annexin *Boschl.Bs8.g11037-ANXA13.2* (Supplementary Fig. 8). This cluster also expresses typical markers of the digestive system including *Gata-a, FoxA1* and *FoxA2* (Supplementary Table 3, Supplementary Fig. 7A^9^).

Cluster 17 was annotated as the nervous system, based on the expression of *Boschl.Bs12.g15790* (Fig. 2G) and a tubulin-α (*Boschl.Bs8.g11041-TUBA3D.2)* (Supplementary Fig. 8), although the latter is also detected in developing oocytes. This cluster also expresses *Zic.r-a*, a progenitor-associated factor enriched in neurogenic territories (Supplementary Table 3, Supplementary Fig. 7A^20^.

Clusters 4, 11 and 32 correspond to epidermal cells, while cluster 31 represents the internal siphon epithelium. These are all marked by *SoxB2* (*Boschl.Bs14.g18200-SOX14.1*, Supplementary Fig. 8) and one of the two cellulose synthase genes (*Boschl.Bs9.g12156*, homologous of *Ciona CesA*). Cluster 4 and 32 represent epidermis of the bud and budlet, based on the expression of *Boschl.Bs8.g11544* (Fig. 2H) and *Boschl.Bs10.g13389* (Supplementary Fig. 8). Cluster 11 corresponds to the vascular epidermis, expressing *Boschl.Bs16.g21151* (Fig. 2I) and *Boschl.Bs9.g12759-SEMA5A.3* (Supplementary Fig. 8). Cluster 31 expresses *Boschl.Bs1.g1247* (Supplementary Fig. 8), marking the internal epithelium of the siphons.

Cluster 12 and 15 correspond to the endostyle and adjacent regions in the branchial epithelium. *Boschl.Bs11.g14583-NKX2-1.2* and *Boschl.Bs5.g6849* are specifically enriched in cluster 14, and both are restricted to the endostyle (Fig. 2J, Supplementary Fig. 8). *Boschl.Bs11.g14583-NKX2-1.2* is potentially orthologous to *Ttf1* from *Ciona intestinalis*, a conserved endostyle marker^39–41^. *FoxQ* (*Boschl.Bs15.g19643-FOXQ1*) is specific to clusters 12 and 15, and labels part of the endostyle, its flanking regions, and part of the branchial epithelium (Fig. 2K).

### In situ hybridization assesses endostyle regionalization

The endostyle in tunicates is a multifunctional pharyngeal structure homologous to the vertebrate thyroid gland^41^, and potentially to other pharyngeal derivatives in vertebrates^42^. In *Botryllus schlosseri*, both the endostyle and the surrounding sub-endostylar region have also been proposed to function as a niche for putative stem cells involved in bud development^24^.

**Fig. 3:**
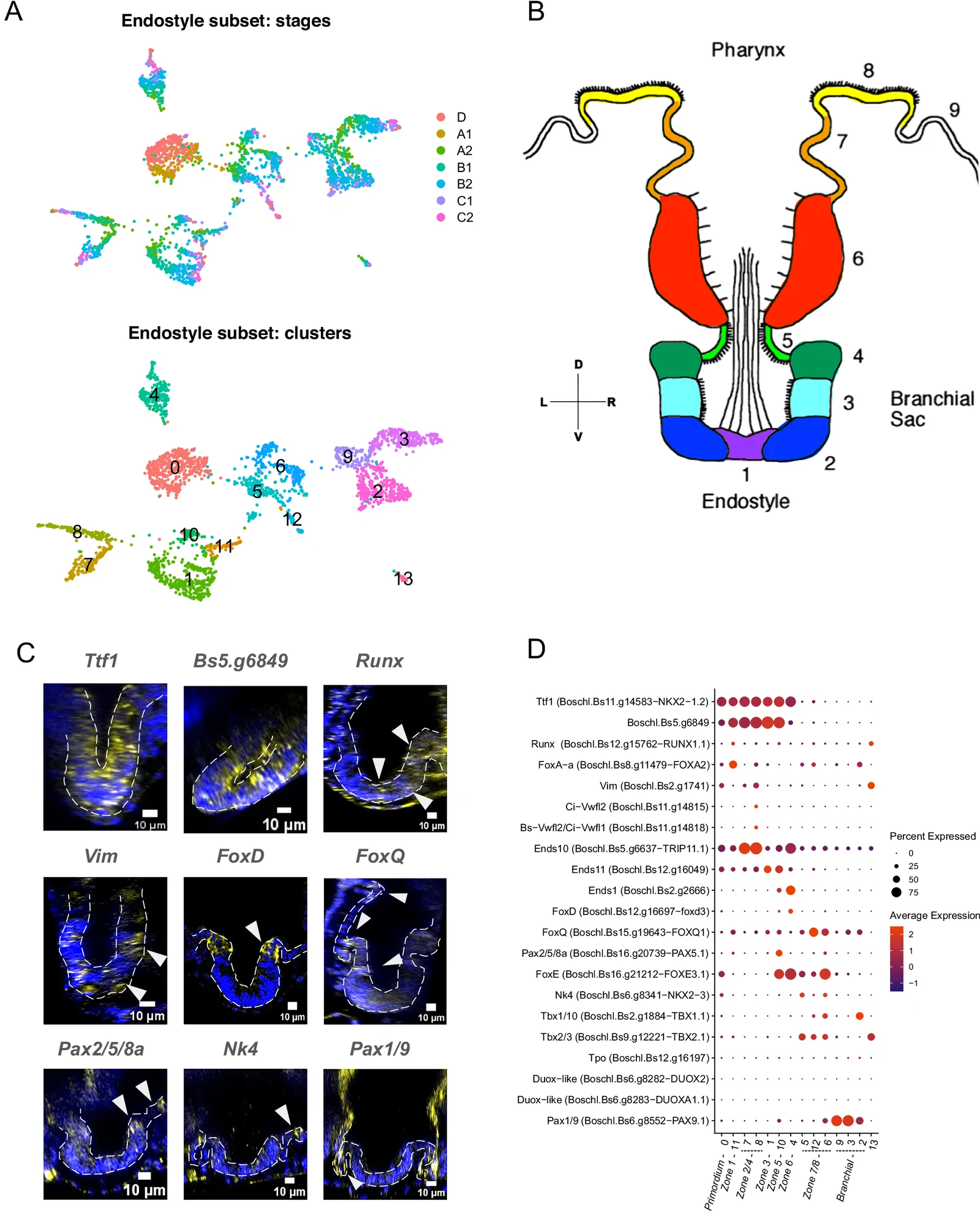
Subclustering and spatial regionalization of endostyle cells. **A** UMAP projection of the subclustered endostyle dataset, colored by developmental budding stage (top panel) and by cluster identity (lower panel). **B** Schematic transverse section of the tunicate endostyle showing its characteristic zonation, with eight histologically distinct bands (modified from Satoh Noriyuki^82^). **C** Confocal images of whole-mount in situ hybridization (WMISH) or hybridization chain reaction (HCR) for selected genes expressed in the endostyle. Arrowheads indicate the spatial domains of transcript expression. Probes in yellow, nuclear staining in blue. **D** Scaled expression of known zone-specific markers across endostyle subclusters, with inferred correspondence between transcriptomic clusters (from **A**) and histologically defined endostyle zones (from **B**). All presented patterns of expression have been observed in at least 3 samples of the same stage.

To investigate the molecular and cellular heterogeneity of endostyle cells in our dataset, we subset and reanalyzed cells from clusters 12 and 15 (Fig. 3A). The tunicate endostyle exhibits a highly organized architecture, composed of distinct zones differing in cellular morphology and associated with specialized functions such as mucus secretion, iodine metabolism, and ciliary movement (Fig. 3B^42,43^). Sub-clustering revealed 14 transcriptionally distinct cell populations. One cluster (cluster 0) was predominantly composed of cells from early stages of endostyle formation (*i.e*., budlet phase D and bud phase A1), while the remaining clusters corresponded to later stages (A2 to C2). In the UMAP, the spatial arrangement of cells within clusters reflects the developmental progression, suggesting that these clusters represent differentiation trajectories of different cell populations (Fig. 3A).

To assign an identity to each cluster, we combined known expression patterns from *B. schlosseri* and *Ciona sp*. with novel data from whole-mount classical ISH and HCR (Fig. 3C, D, Supplementary Table 4). This integrative approach allowed us to propose a correspondence between transcriptomic clusters and histologically defined endostyle zones (Fig. 3D).

Subclusters 7 and 8 likely represent populations of zones 2 and 4, although it remains unclear whether the two clusters correspond to distinct zones or represent shared cell types present in both regions. This is due to the unclear orthology between Von Willebrand Factor genes in *Ciona* and *Botryllus*, which serve as regional markers in the former (Fig. 3D, Supplementary Table 4) and underscores the need for more precise spatial validation.

Although *Tpo* and *Duox*, genes classically associated with zone 7^44,45^, were minimally expressed in our datasets (likely due to the early developmental stages sampled, Fig. 3D), two *Duox* paralogs (*Boschl.Bs6.g8282* and *Boschl.Bs6.g8283*) were specifically detected in a small number of cells in subcluster 6 (Fig. 3D). This suggests that subcluster 6 may correspond to zone 7. Branchial identity was based on the expression of *Pax1/9* (Fig. 3C, D). However, the precise boundaries between zones 7, 8 and 9, as well as their transitions to the surrounding branchial basket epithelium, remain unresolved. Further spatial validation using double HCR might be required to refine our understanding of these regions.

This molecular atlas of the endostyle could serve as a resource to explore the putative niche roles suggested for the endostyle in *B. schlosseri*. For instance, the Wnt pathway has been shown to be involved in short-range signaling and crucial in regulating the niche microenvironment for a large variety of stem cells in vertebrates and invertebrates^46,47^. In our dataset, several *Wnt* and *Fzd* are being expressed in the endostyle clusters, in particular in the one corresponding to zone 5 (subcluster 10, Supplementary Fig. 9).

### The germline multipotency program is germline-restricted

Adult pluripotent or multipotent stem cells are known to underpin various forms of non-embryonic development, namely agametic reproduction and whole-body regeneration, in diverse animals such as acoels, planarians, annelids, cnidarians, and sponges^37,48–50^. In these organisms, adult stem cells typically proliferate and express genes associated with the Germline Multipotency Program (GMP^37^). In tunicates, GMP genes such as *Piwi-2*, *Vasa* and *Pl10*, have been proposed as markers of pluripotent or multipotent cells involved in both agametic development and whole-body regeneration^16,25,28^. Indeed, *Piwi-2* knock-down disrupts vascular budding in *Botrylloides leachii*^16,25^, and both *Piwi-2* and *Vasa* are highly expressed in proliferating cells during the early stages of vascular budding in *B. diegensis*^28^. In contrast, during peribranchial budding in *Botryllus schlosseri*, the expression of *Piwi* and *Vasa* appears to be largely restricted to the germline^25,51^. Only limited protein expression has been reported outside this context, notably in the digestive epithelium and in putative phagocytic cells, only in the adult zooid^52^. Similarly, in *Botryllus primigenus* peribranchial budding *Piwi-2* and *Vasa* expression are restricted to the germline^53^.

**Fig. 4:**
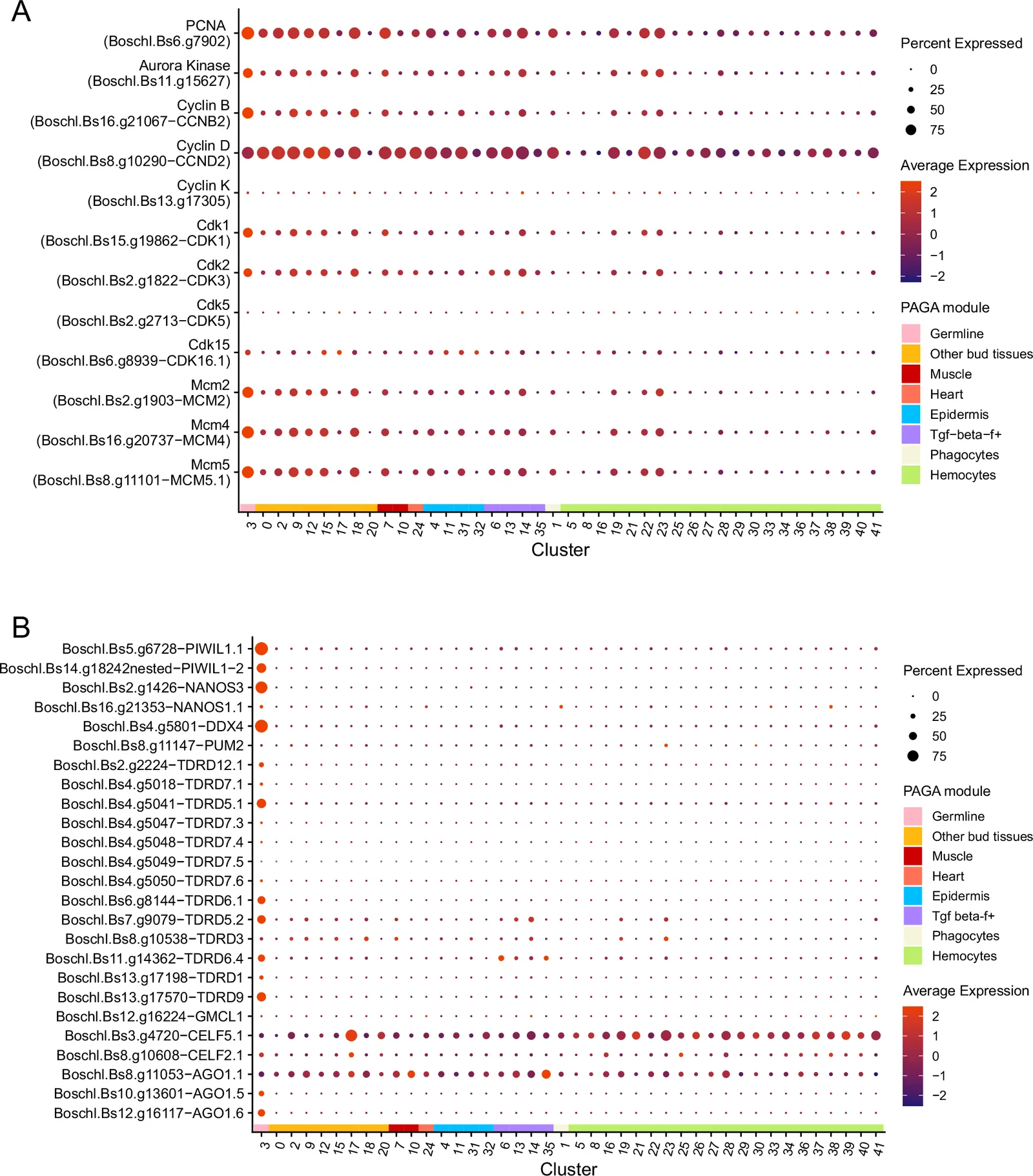
Proliferation markers and germline multipotency program (GMP) gene expression during budding. **A** Scaled expression of proliferation-associated genes across clusters in the budding dataset, highlighting widespread mitotic activity during bud and budlet development. **B** Scaled expression of germline multipotency program (GMP) genes across clusters, showing enrichment in the germline lineage (cluster 3) and weak or partial expression in a limited number of other clusters.

To identify candidate multipotent or pluripotent cells involved in budding, we first examined cluster-specific expression of proliferation markers. Most clusters displayed proliferative signatures (Fig. 4A, Supplementary Fig. 10), consistent with the active growth and morphogenesis occurring in buds and budlets. Immunostaining for phospho-histone H3 further confirmed widespread mitotic activity (Supplementary Fig. 10A). However, this proliferation was broadly distributed and did not reveal a distinct, discrete population of highly proliferative cells.

We then assessed the expression of GMP-associated genes. Co-expression of GMP markers was largely restricted to cluster 3, corresponding to the germline lineage (Fig. 4B). While some other clusters—including those associated with budding tissues— weakly expressed subsets of GMP genes, no additional population showed broad co-expression.

In previous studies, the co-expression of *SoxB1*, *Myc*, and *Pou3*—orthologs of the Yamanaka factors (*Sox2*, *c-Myc*, *Pou5f1*)— was proposed as a signature of adult stem cells in *B. schlosseri*^54^. Another paralog, *Pou2*, has been described as restricted to early budlet and the female germline^54^. In the reference genome used to map our scRNAseq reads^30^, *Pou3* gene was not annotated, while *Pou2* (*Boschl.Bs11.g15669-POU2F1.1*) shows widespread expression, with enrichment in clusters 5, 27, and 40, likely corresponding to amoebocyte populations (^55^; see below). Cells co-expressing *SoxB1* (*Boschl.Bs11.g14812-SOX3.1*) and *Myc* (*Boschl.Bs10.g13375*) were found in clusters 9, 12 and 15, associated with the stigmata, parts of the branchial epithelium, and the endostyle, respectively (Supplementary Fig. 11). These distributions match previous descriptions^54^ but do not support the presence of a distinct pluripotent population or specific links to the origin of the budlet clusters (see below).

### Circulating cells diversity and hematopoietic trajectories

*B. schlosseri* harbors a diversity of circulating cells morphotypes within both its internal (sinuses and lacunae) and extracorporeal vascular systems^56–58^. Because our scRNAseq datasets were generated from whole buds and budlets together with portions of the surrounding vasculature (Supplementary Fig. 1A) –and consistent with detection of vascular epithelial cells (cluster 11, Figs. 1C, 2I)– we hypothesized that circulating cells were also captured within the budding datasets.

To identify clusters corresponding to circulating cell types, we generated two additional scRNAseq datasets from cells directly isolated from the vasculature by bleeding out entire *B. schlosseri* colonies (see Methods). These cells were processed immediately for scRNAseq, without dissociation, FACS sorting, or enrichment. Quality control for the circulating-cells datasets is shown in Supplementary Fig. 2 and detailed in the Methods section.

**Fig. 5:**
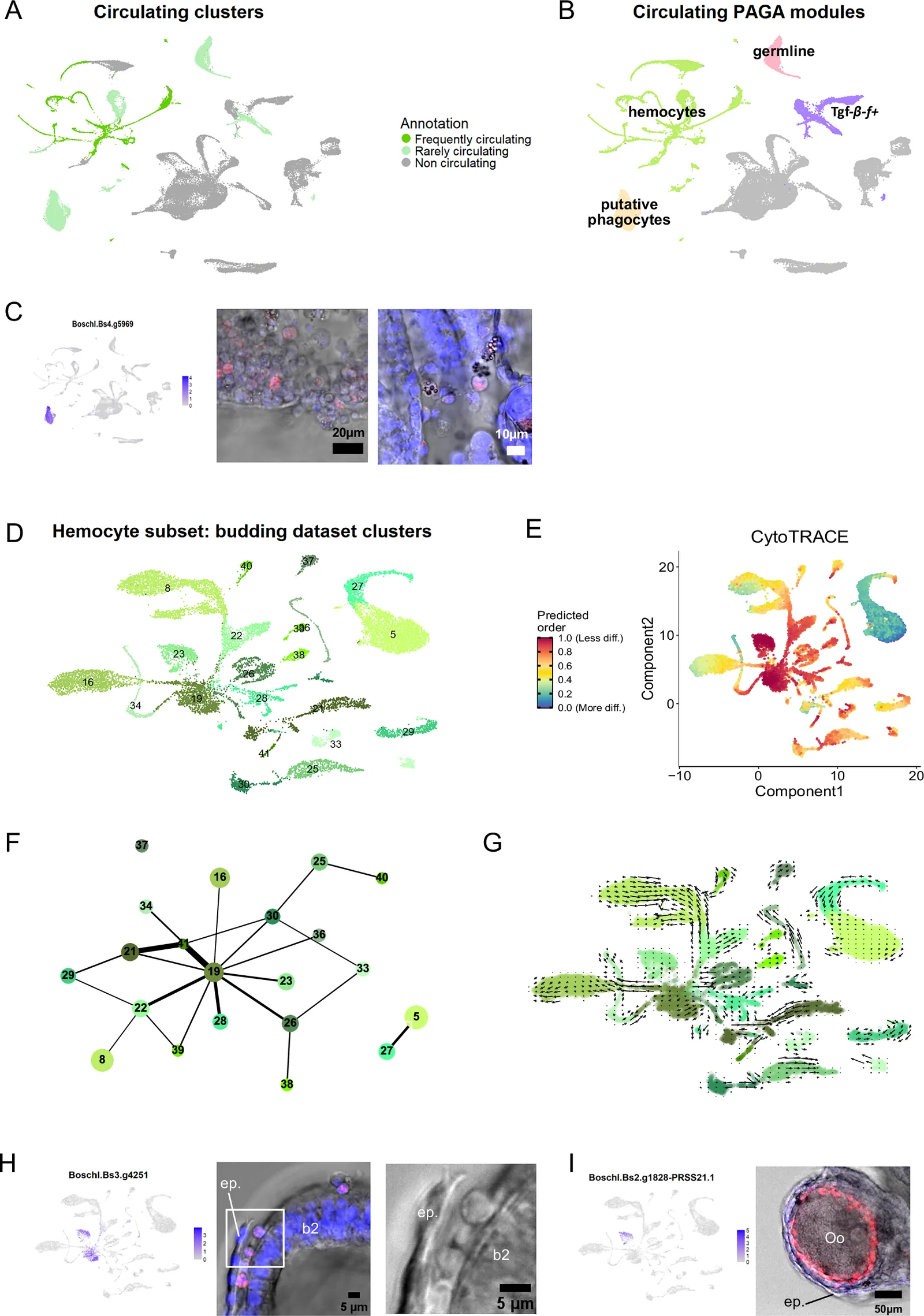
Diversity of circulating cells and differentiation trajectory inference. **A** UMAP projection of the budding dataset, colored with the clusters' circulating status from Supplementary Fig. 12C. **B** UMAP projection of the budding dataset colored according to the 4 corresponding PAGA groups of Fig. 1E. **C** Expression plots and HCR of a marker gene of the putative phagocytes cluster. **D–G** Separate analysis of the hemocyte module subset. **D** UMAP projection with cluster numbers of the full budding dataset (Fig. 1E) and **E** corresponding CytoTRACE score, **F** abstracted graph (PAGA), **G** projected RNA velocity vectors. **H–I** Expression plots (color represents normalized expression of the gene) and HCR of marker genes of (**H**) putative hemoblast (cluster 19) and resident hemoblast-like (cluster 23), or (**I**) resident hemoblast-like only (cluster 23). For HCR pictures: the probe signal is in red, Hoechst staining in blue, and the transPMT in gray. Ep epidermis, b2 budlet, Oo oocyte. All presented patterns of expression have been observed in at least 3 samples of the same stage.

Combined analyses of the budding and circulating cell datasets (Supplementary Fig. 12A, B) enabled us to determine which clusters contain circulating cells. Clusters with <1% of cells coming from the circulating dataset were considered “non-circulating” (predominantly epithelial or sessile mesenchymal); those with 1–10% were “rarely circulating”, likely correspond to mesenchymal populations largely resident in buds, budlets, or ampullae that circulate only rarely or transiently; and those with >10% were “frequently circulating” (Supplementary Fig. 12B, C). The circulating status was then mapped back onto the budding datasets UMAP (Fig. 5A).

PAGA analysis of the full budding dataset showed that the “rarely circulating” and “frequently circulating” clusters belong to four discrete, minimally connected, modules (Fig. 1E, highlighted in the UMAP of Fig. 5B), suggesting either that intermediate states were not captured or that these modules represent separate lineages. One PAGA module corresponds to the germline and a second encompasses the *Tgf-β-f+* clusters together with the “rarely circulating” small cluster 35, which strongly expresses the histocompatibility-related gene *Bhf*^59^ (Figs. 1E, 5B). Among the *Tgf-β-f+* cells, support cells are physically attached to mobile germline precursors and developing oocytes that circulate at phase B2 to colonize newly forming budlets^15,51^. Consistent with this transient migration, only one *Tgf-β-f+* cluster was classified as “rarely circulating” (Fig. 5B; Supplementary Fig. 12B, D^51^). A third module includes a single large “rarely circulating” cluster. HCR of its marker gene, *Boschl.Bs4.g5969*, revealed expression in cells of variable morphology, some of which are at least 10 µm in diameter, and are reminiscent of described phagocyte morphology (Fig. 5C^60,61^). These cells are likely to be mostly resident in tissues, as they are present in much higher numbers in our budding datasets than in the circulating dataset (Supplementary Fig. 12). GO terms enriched in this cluster include terms related to innate immune response, such as neutrophil degranulation (GO:0043312) and terms related to migration and localization of the cell (GO:0051674, GO:0030335) (Supplementary Fig. 13A, B). Hence, we annotated this cluster as putative phagocytes.

The fourth and largest module includes all the “frequently circulating”, a few “rarely circulating,” and two “non-circulating” clusters and was annotated as hemocytes. These clusters are highly connected in the PAGA graph (Figs. 1E; 5A, B), suggesting they belong to a common –possibly hematopoietic– lineage. We used genes shown to be expressed in hemocytes to identify some of these clusters. Cluster 16 expresses a phenoloxidase (*Boschl.Bs1.g975-PPO1*), a known marker for granular amoebocytes and morula cells (Supplementary Fig. 13C). Cluster 29 expresses one described *Botryllin* paralog (*Boschl.Bs11.g14579*^62^), also associated with morula cells morphotype (Supplementary Fig. 13C). Clusters 5 and 27 express the Kringle-domain containing protein *Boschl.Bs1.g1030*; HCR revealed an amoebocyte morphology with few vacuoles, consistent with hyaline amoebocyte morphotype^56,60^ (Supplementary Fig. 13C, negative controls in Supplementary Fig. 13A). These cells localize both in circulation and within the tunic, especially around the ampullae (Supplementary Fig. 13C). These clusters also express a cellulose synthase (*Boschl.Bs9.g12156*), suggesting a role in tunic turnover (Supplementary Fig. 13C). GO terms enriched in this cluster are diverse, and include apoptotic cell clearance (GO:0043277, Supplementary Fig. 13B). Notably, these clusters, as well as in cluster 21, express *Athena (Boschl.Bs11.g14504)*, a gene that was shown to be necessary at phase D/A1 for future budding organogenesis (Supplementary Fig. 13C^63^).

To identify potential progenitors, and to reduce transcriptomic heterogeneity for more robust trajectory analyses, we isolated the PAGA hemocytes module (Fig. 1E, Fig. 5B) and re-analyzed it separately from the rest of the budding dataset (Fig. 5D). CytoTRACE ranked clusters 19 and 23 highest in inferred developmental potential (Fig. 5E, Supplementary Fig. 14A). Consistent with a progenitor-like status, these clusters show strong expression of proliferation markers (Fig. 4A) and are enriched for cell-cycle and hematopoietic GO terms (e.g., “G1/S transition of mitotic cell cycle,” GO:0000082; “hematopoietic or lymphoid organ development,” GO:0048534; Supplementary Fig. 14B). Cluster 19 also occupies a topologically central position in the PAGA graph (Fig. 5F). RNA velocity analyses using scVelo yielded a vector field broadly consistent with differentiation radiating from clusters 19 and 23, but the limited fraction of unspliced reads reduces resolution; we therefore consider velocity as supportive but not decisive evidence (Fig. 5G). Together, these analyses strongly suggest that clusters 19 and 23 represent hematopoietic progenitor populations. Hemoblasts, previously described in *B. schlosseri* and other tunicates as putative progenitors for every type of hemocytes, are small (~5 µm), round cells, with a high nuclear-to-cytoplasmic ratio and classical undifferentiated morphology^56,60,61^. HCR on *Boschl.Bs3.g4251*, enriched in clusters 19/23 (Fig. 5H), and *Boschl.Bs3.g4360*, enriched in clusters 19/28 (Supplementary Fig. 14 C), confirmed their expression in cells with hemoblast-like morphology, further supporting this identity. The genes *Boschl.Bs2.g1828-PRSS21.1* and *Boschl.Bs13.g17339* enriched in cluster 23, are notably expressed in “test-like” cells surrounding the more mature oocytes (Fig. 5I; Supplementary Fig. 14C), under the first layer of follicular cells. This localization is consistent with their “non circulating” status detected above (Fig. 5A; Supplementary Fig. 12B, C). Negative controls are shown in Supplementary Fig. 14E.

Our integrative analyses identified a broad identity of circulating cells in *B. schlosseri*, most of which are part of a common hematopoietic-like lineage, and genes that are specifically expressed in probable stem cells or progenitors of the hematopoietic lineage. These results are consistent with previous evidence that *B. schlosseri* harbors a complex vertebrate-like hematopoietic system^57^.

### Identification of founder cells initiating budding

**Fig. 6:**
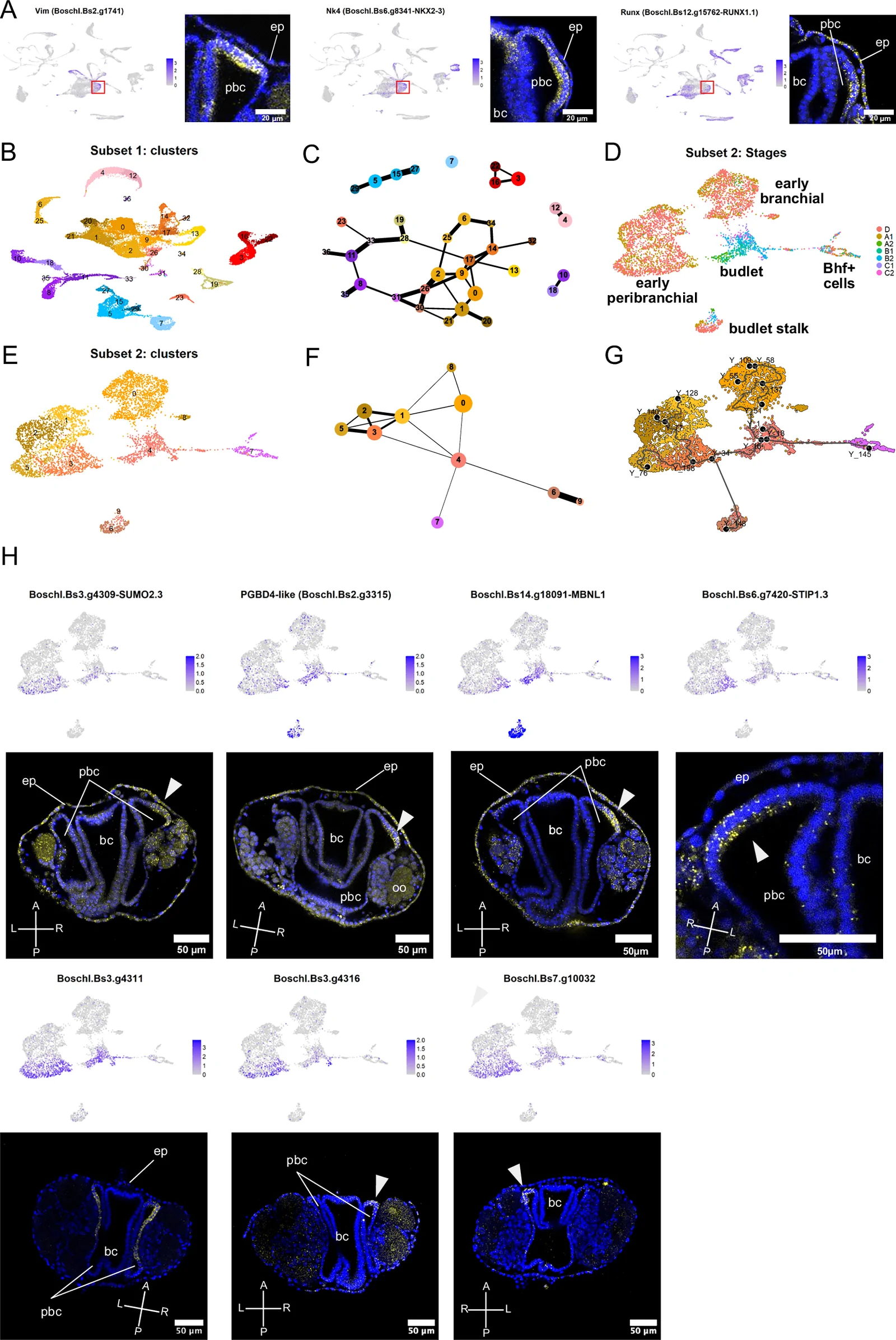
Budding founder cells in the peribranchial epithelium have a specific transcriptomic signature. **A** Expression plots (color represent normalized expression of the gene) and corresponding HCR at stage D/A1 of the known budlet markers *Vim*, *Nk4,* and *Runx*. The budlet cells population is squared in red. **B** UMAP projection of the subset without hemocytes and putative phagocytes (subset-1), showing cluster numbers of this subset. **C** Corresponding abstracted graph (PAGA) for subset-1. **D, E** UMAP projection of the subset-2 (obtained from the clusters 2, 9, 26, 30, and 31 of subset-1), showing stages (**D**) and sub-clustering of the subset-2 (**E**). **F** Abstracted graph (PAGA) of the subset-2 clusters. **G** Monocle3 inferred trajectories for subset-2. **H** Expression plots (color represents normalized expression of the gene) and corresponding HCR of marker genes of the budding founder cells (arrowhead) in stage C2/D budlets. Probes in yellow, nuclear staining in blue. Ep epidermis, pbc peribranchial chamber, bc branchial chamber, oo oocytes. All presented patterns of expression have been observed in at least 3 samples of the same stage.

To identify the earliest budlet-forming cells, we implemented a marker-guided, stage-resolved analytical framework, combining iterative sub-setting and re-clustering with spatial validation. First, in the full budding dataset, we screened for the co-expression of three genes previously reported as budlet markers: the intermediate filament *Vim* (*Boschl.Bs2.g174*^10^), and the transcription factors *Runx (Boschl.Bs12.g15762-RUNX1.1*^10,36^) and *Nk4* (*Boschl.Bs6.g8341-NKX2-3*^2,21^). Within the early branchial/peribranchial clusters 2 and 20, cells strongly co-expressing all three markers were confined to a small population (Figs. 6A, 1C, Supplementary Fig. 15A), spanning stages D to C2, consistent with these cells representing secondary bud (budlet) populations in our dataset (Fig. 1D; Supplementary Fig. 5A).

Second, to increase resolution within budding tissues, we removed from the whole budding dataset the hemocyte and putative-phagocyte modules (Fig. 5B) and reanalyzed the new subset, hereafter referred as “subset-1” (Fig. 6B; correspondence between original clusters and new clusters are shown in Supplementary Fig. 15B, C). In subset-1, budlet cells from phase A1-C2, identified by *Vim/Runx/Nk4* co-expression (Supplementary Fig. 15D) and their stage gradient (Supplementary Fig. 15E), emerged as a distinct cluster (cluster 26) branching from the early peribranchial population (cluster 2, *Pax2/5/8a*-positive; Fig. 6B). PAGA analyses of subset-1 confirmed strong connectivity between the budlet cluster 26 and early peribranchial cluster 2, early branchial cluster 9 (*Pax1/9*-positive), the stalk septum cluster 30, and cluster 31 (*Bhf*⁺ cells), with the *Bhf*⁺ cells forming a transcriptional intermediate to the *Tgf-β-f⁺* clusters (Fig. 6C). In addition, both the heart (cluster 23) and the early neuronal progenitor (cluster 28) clusters show transcriptional proximity with the *Tgf-β-f+* clusters. Expression of *Mbnl1-like* (*Boschl.Bs14.g18091-MBNL1*), *Vim* (*Boschl.Bs2.g174*), and *Runx (Boschl.Bs12.g15762-RUNX1.1)* in the stalk septum (Supplementary Fig. 15F, G) revealed that this structure remains continuous to the peribranchial epithelium, rather than being a fold of the epidermis as suggested by Burighel and Brunetti^64^ (Supplementary Fig. 15G).

Then, from subset-1 we selected the five subclusters strongly connected to the budlet (*i.e*. clusters 2, 9, 26, 30, 31), reanalyzed them, and generated a second subset, here referred as subset-2 (Fig. 6D, E; Supplementary Fig. 16A). The resulting UMAP resolved budlet cells from phase A2-C2 (labeled as “budlet” in Fig. 6D, subset-2 cluster 4 in Fig. 6E) diverging from a small, distinct population of early peribranchial cells at stages D–A1 (subset-2 cluster 3, Fig. 6E). PAGA analyses of subset-2 showed connectivity between the budlet cluster and early peribranchial clusters 1 and 3 (Fig. 6F). Trajectory inference with Monocle3 (Fig. 6G), together with the staging gradient (Fig. 6D), identified cluster 3 as the most likely founder population, whereas RNA velocity (scVelo) predicted incongruent directionalities for this subset (Supplementary Fig. 16B).

To confirm spatial localization, HCR for founder-enriched transcripts - *Sumo2*-*like* (*Boschl.Bs3.g4309-SUMO2.3*), *Pgbd4*-*like* (*Boschl.Bs2.g3315*), *Stip1*-*like* (*Boschl.Bs6.g7420-STIP1.3*), *Mbnl1-like* (*Boschl.Bs14.g18091-MBNL1*), *Gnrhr2*-*like* (*Boschl.Bs15.g20225*) as well as the unannotated *Boschl.Bs3.g4311, Boschl.Bs3.g4316* and *Boschl.Bs7.g100032* - showed signal in a peribranchial domain anterior to the gonadal primordium as early as phase C2/D, *i.e*., the site of budlet onset^7^ (Fig. 6H; Supplementary Fig. 17). Double HCR further showed that these founder markers co-localize within the same cell population (Supplementary Fig. 18A).

**Fig. 7:**
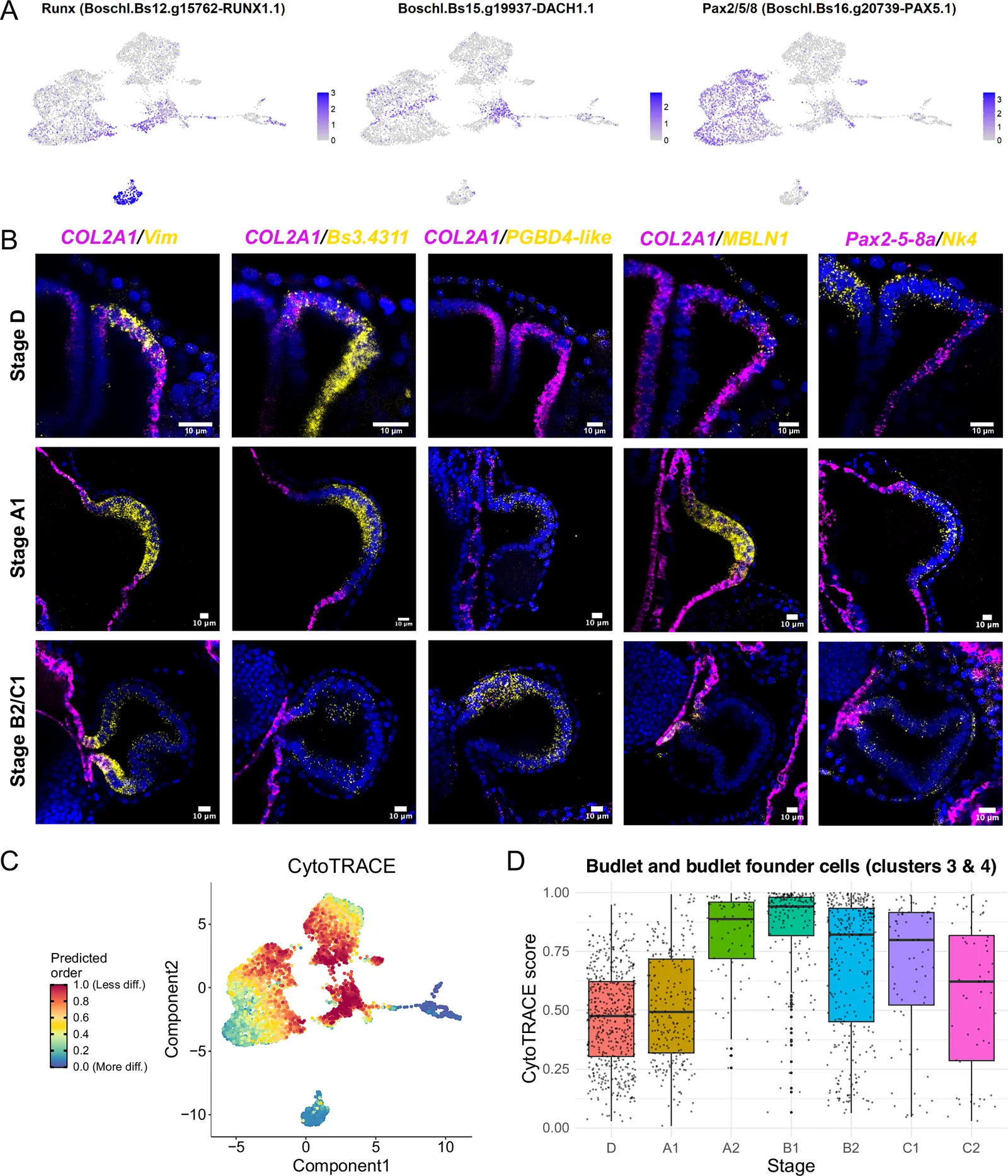
Transcriptional dynamics from budlet founder to budlet cells shows a transcriptomic switch. **A** Expression plots of transcription factors expressed in the budding founders and/or budlet with different dynamics, color represents normalized expression of the gene. **B** Double HCR of differentiating peribranchial epithelia marker genes *Boschl.Bs16.g20741-COL2A1* and Pax 2/5/8a (*Boschl.Bs16.g20739-PAX5.1*) (magenta) and budlet founder markers Vim (*Boschl.Bs2.g174*)*, Boschl.Bs3.g4311, Boschl.Bs14.g18091-MBNL1, Pgbd4-like* (*Boschl.Bs2.g3315*)*, Nk4* (*Boschl.Bs6.g8341-NKX2-3*) (yellow) **C** CytoTRACE score of the cells in the subset-2. **D** Evolution of CytoTRACE score of budding founders and budlet cells (clusters 3 and 4 of subset-2) across stages (*n* = 1480 cells, with 56-518 cells per stage). The boxplot shows the median (line in the box), the first and third quartiles (the hinges of the box), and the largest/smallest value no further than 1.5 * inter-quartile range from the corresponding hinge (the whiskers), as well as any outlier beyond that point as bold points. All presented patterns of expression have been observed in at least 3 samples of the same stage.

Transcriptional dynamics across the peribranchial-to-founder transition indicated a coordinated program switch. *Runx* is activated in founders and remains expressed throughout early budlet development (Figs. 6A; 7A); a *Dach1-like* gene (*Boschl.Bs15.g19937-DACH1.1*) is OFF in founders and ON in budlets from stage B1 (Fig. 7A), whereas peribranchial identity markers including *Pax2/5/8a* (*Boschl.Bs16.g20739-PAX5.1*) and *Spp1-like* (*Boschl.Bs8.g11353-SPP1*), and the structural collagen *Col2a1-like* (*Boschl.Bs16.g20741* = *COL2A1*) remain ON in at least some of the peribranchial epithelium but are extinguished in budlets (Figs. 2B, C; 7A, Supplementary Fig. 18B–D). Double HCR combining peribranchial epithelium markers *Col2a1-like*, *Pax2/5/8a*, which are homogeneously expressed in buds (phases A-D) and budlets at stage C, with the founder-associated genes (*Vim Boschl.Bs2.g174,Boschl.Bs3.g4311*, *MBNL1*, *Pgbd4-like*, *Nk4 Boschl.Bs6.g8341-NKX2-3*), confirm the transition (Fig. 7B, Supplementary Fig. 19). Regulators of post-transcriptional and post-translational control, such as splicing regulator *Mbnl1-like* (*Boschl.Bs14.g18091-MBNL1*), co-chaperone *Stip1-like* (*Boschl.Bs6.g7420-STIP1.3*), and SUMOylation component *Sumo2-like* (*Boschl.Bs3.g4309-SUMO2.3*) are upregulated in founders and early budlets (Fig. 6H). This suggests active RNA processing, proteostasis, and SUMO-dependent remodeling during the peribranchial-to-founder transition. Moreover, CytoTRACE scores across the subset-2 showed a characteristic arc for founder and budlet cells, rising from phase D to B1 and then declining, while other clusters lacked this pattern and generally scored lower (Fig. 7C, D), consistent with a transient, less-differentiated state in early budlets and compatible with a potential dedifferentiation step as peribranchial epithelial cells transition into budlet founders.

## Discussion

The multi-stage dataset presented here provides an unprecedented view of agametic development in tunicates, offering an important resource for understanding the cellular and molecular mechanisms underlying budding. The dataset captured a wide diversity of cell types associated with bud onset and development, with clear annotation of major clusters supported by robust gene expression profiles. This not only validates previously described expression patterns but also enables high-resolution sub-clustering of complex tissues and allows for evaluating and revisiting developmental hypotheses with broader transcriptomic and genomic context.

One notable example is the endostyle, where transcriptomic clustering allows subregional identification. This dataset also reveals the nature of under-described anatomical structures like the budlet stalk septum, which likely derives from the peribranchial epithelium and remains connected to the budlet beyond phase B2. Similarly, developmental trajectories were observed across multiple lineages, including the muscle and nervous system, where gene expression gradients reflect known stage-specific differentiation events.

Despite the strengths of the dataset, some limitations must be acknowledged. For instance, it is important to note that some stages are less comprehensively represented than others. In particular, fewer cells were collected and sequenced for the budlet phases C1 and C2, periods marked by key events in lineage commitment and early differentiation. Also, although the primary bud at phase C2 is nearing functional maturity, certain cell types or states specific to fully developed adults or to unrepresented vascular regions may be underrepresented or absent from our dataset. Additionally, the FACS gating strategy, which excluded haploid and potentially polyploid cells, resulted in the absence of mature gametes, limiting insights into the full germline spectrum.

In exploring the origin of cells contributing to budding, we focused on identifying candidate progenitors based on proliferation signatures, expression of germline multipotency program (GMP) genes, and homologs of Yamanaka factors^54^. While tunicate budding has been linked to contributions from both mesenchymal and epithelial cells (e.g.^23^), our data suggest no clear molecular evidence for a distinct pluripotent stem cells progenitor population. Although we cannot exclude the presence of GMP markers in rare cell populations, such expression was largely restricted to the germline cluster. Moreover, cells co-expressing *SoxB1* and *Myc-*like were restricted to tissues such as the endostyle and stigmata, without forming a distinct pluripotent population. We also started exploring the role of the endostyle, previously proposed as a stem cell niche^24^, and found expression of several *Wnt* and *Fzd* genes, suggesting active signaling in the developing endostyle.

Given that in different colonial tunicates the putative stem cells involved in budding have been proposed to be circulating^1,23,25,28,65^, we examined in greater detail the populations of circulating cells. In *B. schlosseri* specifically, grafting experiments demonstrated that rare, potentially circulating cells can give rise to either somatic or germline tissues, but not both^23^. By comparing our budding dataset with a reference dataset of circulating cells, we identified the clusters corresponding to cell types present in circulation. As expected, both germline and somatic cell populations were represented. Notably, the germline cluster appeared transcriptionally distinct and distant from the somatic clusters, consistent with the well-documented separation between somatic and germline lineages in tunicates^66^ as well as previous grafting experiments^23^.

Interestingly, a substantial number of the circulating cell clusters form a transcriptionally interconnected group, suggestive of ongoing differentiation trajectories. Within this group, we identified two clusters likely corresponding to the previously described hemoblasts, undifferentiated blood cells thought to harbor stem-like potential^60^.

The diversity of cell populations revealed by scRNAseq exceeds the eight morphotypes historically described based on morphology^67^. This higher diversity is consistent with recent descriptions of *Ciona robusta* blood cell diversity^68^ and with populations identified by Rosental and colleagues in *B. schlosseri* using FACS-based profiling^57^. Future efforts could aim to directly align those sorted populations with our transcriptomic clusters.

Despite the identification of putative hemoblast clusters, PAGA analysis did not reveal strong links between these clusters and the main epithelial tissues lineages of the developing bud, though lowering the connectivity threshold (Supplementary Fig. 6E) suggested possible connections between cluster 23 (resident hemoblast-like cells) and cluster 12 (branchial/peribranchial and lower quality cells), and connections between an unidentified hemocyte cluster (cluster 38), and cluster 20 (late branchial/peribranchial and stalk septum cells) or cluster 13 (*Tgf-β-f*⁺). The significance of these connections and whether they reflect an actual biological link remains uncertain and may reflect either rare transitional states or technical limitations in capturing transient intermediates.

A particularly intriguing observation is the transcriptional diversity within the *Tgf-β-f*⁺ cell population, which appears to comprise at least three transcriptionally distinct subtypes. These may correspond to different functional identities, potentially including germline support cells, somatic components of the testis, and follicular cells^36^. PAGA analysis in subset-1 revealed transcriptomic proximity of these clusters to early neuron and heart lineages (Fig. 6C), suggesting possible developmental links. While this proximity does not demonstrate lineage contribution, it raises the hypothesis that certain *Tgf-β-f*⁺ cells may contribute to, or come from, specific organ lineages during organogenetic phases of budding.

Importantly, these observations leave open alternative interpretations consistent with previous studies proposing a role for circulating, multipotent or pluripotent stem-like cells in colonial tunicates^23–25^. In this framework, *Tgf-β-f*⁺ cells or other mesenchymal populations could represent a transient or context-dependent source of progenitors contributing to budding, potentially acting as a mesenchymal complement to epithelial-derived founder cells.

Altogether, these observations indeed support the presence of circulating stem-like cells in *B. schlosseri*. However, we do not find clear transcriptomic or trajectory-based evidence for a dominant or obligatory contribution of these populations to the formation of most somatic tissues of the bud.

We identified a small population of cells corresponding to the budlet founders and early budlet characterized by a previously described specific combination of gene expression features within the early peribranchial epithelium^2,10,21,36^. CytoTRACE analysis of budlet founders and budlet cells inferred an increase in developmental potency from budlet onset (phase D) to budlet phase B2. Since CytoTRACE scores are partly based on the number of genes expressed, often higher in less differentiated cells with more open chromatin^31^, this trend is consistent with a progressive gain of potency during the early stages of budding. However, transcriptional data alone do not allow a direct assessment of functional potency. Therefore, while our observations are compatible with a model in which founder cells acquire increased developmental potential through reprogramming mechanisms, they do not exclude alternative explanations. For instance, transient contributions from lineage-restricted mesenchymal cell populations, or the presence of rare multipotent progenitors not captured in our dataset.

Within these limitations, these findings are most consistent with a scenario in which the founder cells are not intrinsically multipotent but may acquire such potential through reprogramming mechanisms during early bud development. This aligns with features commonly associated with dedifferentiation, which typically involve changes in gene expression programs, protein composition, cell morphology, and potency^69^.

Our data further reveal a marked switch in transcriptomic identity in these founder cells and early budlet cells. Genes typically expressed in the peribranchial epithelium, such as the transcription factor *Pax2/5/8a*, the PHF zinc finger protein *Boschl.Bs8.g11353-SPP1*, and the collagen *Boschl.Bs16.g20741-COL2A1*, are downregulated. Concurrently, other genes become upregulated, including *Runx* and, slightly later, *Nk4*.

Within the peribranchial epithelium at phases C2 and D, *Sumo2-like* (*Boschl.Bs3.g4309-SUMO2.3*) is expressed exclusively in the founder population. SUMOylation is a post-translational modification involved in diverse cellular processes^70^, and has been shown to play a key role in stabilizing both stemness and differentiated cell states^71,72^. Additional genes involved in post-transcriptional and proteome regulation are also enriched in the founder cells and early budlets, including the splicing regulator *Mbnl1-like* (*Boschl.Bs14.g18091-MBNL1*) and the E3-ubiquitin ligase *Boschl.Bs7.g9551*, suggesting active remodeling of the proteome and potential regulation of cell state transitions.

Several signaling pathways have been implicated in budding in styelid ascidians such as *B. schlosseri*, although functional studies often yield complex phenotypes that are challenging to interpret. Notably, a *Gnrhr2*-like gene is transiently expressed in founder cells and early budlets, as well as in the gonads, nervous system, and other cell populations. In vertebrates, gonadotropin-releasing hormones (GNRHs) and their receptors (GNRHRs) regulate the hypothalamus–pituitary–gonadal axis, particularly in response to the organism’s metabolic state^73^. In invertebrates, which lack this axis and gonadotropins, GNRHs are thought to act directly as neuropeptides modulating both reproductive and non-reproductive functions^74^. In *Ciona*, GNRH signaling has been implicated in both reproduction and metamorphosis. In *Botryllus*, the number of buds and the extent of gonad development are known to vary with environmental conditions, as well as with others factors such as age^4^. These observations suggest that a GNRH signaling axis may play a dual role in coordinating budding and reproductive development in response to the colony’s metabolic state.

In summary, the multi-stage single-cell atlas of *Botryllus schlosseri* agametic development provides a transcriptomic roadmap of peribranchial budding at single-cell resolution. Our data identify a distinct founder population emerging from the peribranchial epithelium, suggesting a model of budding triggered by context-dependent dedifferentiation and remodeling of committed somatic tissues rather than a dedicated pool of multi-/pluripotent stem cells. While we identified circulating hemoblast-like populations with potential stem-like features, our analysis did not reveal clear broad contributions of these cells to bud onset beyond the hematopoietic or gonadal lineages, although transient intermediate states may remain undersampled. By integrating trajectory analyses, founder cell signatures, and pathway expression profiles, we also propose candidate regulatory mechanisms, including GNRH-like signaling, that may couple environmental and metabolic cues to the initiation of budding. Altogether, this work expands our understanding of non-embryonic development in chordates and establishes a rich resource for future studies of regeneration, stemness, and plasticity in a vertebrate sister group model.

## Methods

### Animal collection and husbandry

All procedures complied with relevant institutional and national regulations. As this study was conducted exclusively on marine invertebrates (*Botryllus schlosseri*), no ethical approval was required.

To ensure genetic homogeneity for the budding samples, young oozoids were collected on glass slides from the port of Villefranche-sur-Mer (43.697882, 7.30735), then isolated in small containers (~1 L) at 18 °C to let them grow for a few weeks. They were fed with a mix of live *Tisochrysis lutea*, live *Chaetoceros gracilis*, ISO800, and Shellfish Diet 1800 (Reed Mariculture). The water was changed about once a day. Once the colonies had 10-20 blastozooids, they were transferred to a cold marine culture system (12 °C) described previously^36^. One of these isogenic colonies (labeled as FC*) was chosen to perform all the dissociations for the budding samples. For the circulating cell samples, animals were collected in Santa Barbara, California, USA. They were kept in a semi-flow-through mariculture system with 0.5µm-filtered seawater at 16-18 °C, and they were fed twice a day with cultures of the following live microalgae: *Dunaliella sp*. (Carolina Biological CAT#152160), *Isochrysis sp*. (Carolina Biological CAT#153180), *Nannochloropsis sp*. (Carolina Biological CAT#153220), *Tetraselmis sp*. (Carolina Biological CAT#152610). Colonies were attached to glass slides with sewing thread and allowed to grow onto the slides. The slides were kept vertically in aquarium tanks. Slides were cleaned every few days to a week with a paint brush, toothpick, and razor blade as needed to remove decay, debris, and microbe/parasite growth. Colonial animals were allowed to grow for a couple of months in the lab. They were then shipped overnight to Boston, at which point they were kept in artificial seawater (ASW, Instant Ocean SS15-10) at 17 °C.

### Sample collection and dissociation

Colonies were staged according to^8^ under a stereomicroscope. Animals in a colony develop somewhat synchronously. Each budding cycle begins with the formation of a new body. A new budlet initially appears as a disc of thickened cells (phase A1) in a specific region of the primary bud’s peribranchial epithelium, anterior to the gonad primordium. The budlet then grows and arches anteriorly relative to its parental bud (phase A2/B1), forming a transient, double-vesicle structure still connected with a stalk to the peribranchial epithelium (phase B2). As development progresses, epithelial folds reorganize the budlet into a more compartmentalized form (phase C1), eventually leading to a structure with distinct heart, gut, and neural rudiments (phase C2). In phase D, the budlet rotates to align with the primary buds and zooids, adopting a morphology similar to the adult. Organogenesis and growth continue until the bud reaches the zooid stage. The transition between primary bud and zooid is defined by the opening of the siphons and initiation of feeding, and from that point each zooid has a defined lifespan (seven days at 18 °C), after which it dies when every cell in every tissue undergoes synchronized apoptosis, the cell corpses are removed by phagocytic cells in the blood, and the zooid body is resorbed^7^. This process, called takeover, occurs in a synchronized fashion throughout a colony; thus, every week, a generation of zooids dies and is replaced by the primary buds. The secondary buds (or budlets) become primary buds, and initiate a new generation of secondary buds. Takeover occurs repeatedly during the lifespan of a genotype. Consequently, to maintain homeostasis, each zooid must be replaced by at least a primary bud. Colonies expand as each primary bud can give rise to 1-4 secondary buds, depending on environmental conditions.

For the budding dataset, samples selected for dissociation (either phases A1 to C2 buds with budlets or phase D budlets, Fig. 1A, B) were microdissected along with their surrounding tunic and some ampullae using fine syringe needles (30 G, Microlance 3). Dissections were carried out in 5µm-filtered seawater maintained between 12-15 °C, with frequent water replacement. To minimize the time between dissection and fixation, each dissociation batch included no more than 10-12 buds/budlets. Particular care was taken to avoid damaging the budding tissues during dissection to limit cellular stress. Multiple rounds of dissection were performed on the same day to increase the number of collected cells. Dissected samples were transferred to a glass 9-well plate cleaned with RNase-AWAY (Fisher) and washed twice in calcium- and magnesium-free artificial sea water (CMFASW; 31 g/L NaCl, 0.8 g/L KCl, 0.29 g/L NaHCO3, 1.6 g/L Na2SO4, pH 8.0) with 40U/ml RNAsin Plus (Promega). Samples were then incubated in 0.2µm-filtered 1% Trypsin (Sigma, T-9201) in CMFASW with 40 U/ml RNAsin Plus for 20–30 min. During enzymatic digestion, the tunic was separated from the tissues with needles, then tissues were mechanically dissociated by vigorously pipetting with a p200 pipet at least 30 times. Cell suspensions were then transferred into 2 mL Protein-LoBind tubes (VWR) kept on ice. A small aliquot was taken for quality control (QC), while the remainder was immediately fixed in ACME (10% acetic acid, 15% methanol, 10% glycerol in molecular grade water^75^) with 40U/ml RNAsin Plus for 1 h on ice, gently shaking. The QC aliquot was stained with Erythrosin B (0.25 mg/ml in CMFASW final concentration) and counted using a Neubauer chamber grid to assess cell viability and concentration. Only preparations with over 85% cell viability were retained for further processing. Following fixation, samples were stored at -20 °C for up to a few days. Fixative was removed by diluting the samples in 0.2µm-filtered 1% BSA (Sigma, A-7906) in PBS, followed by centrifugation (5 min at 800 g, 4 °C). After carefully removing the supernatant, samples from the same dissociation batch were pooled together during resuspension in 1% BSA in PBS with 40U/ml RNAsin Plus. DMSO was then added to a final concentration of 10% for cryopreservation, and samples were stored at −80 °C for up to a month.

For the circulating cells samples, colonies at phases A1 or B2 were cleaned a couple hours before dissections. A Kim wipe was used to wipe slides, and a small paintbrush and toothpick were used under a dissecting microscope to remove biofilms, algae, parasites, dead tunic sections, and other debris near the colony. Any regions of the colony with decay were removed with a razor blade. Finally, colonies were dipped in deionized water for a few seconds to lyse any remaining bacteria and parasites. Cleaned colonies were kept in 0.22 µm filtered ASW until blood collection. Slides were dried gently with a Kim wipe. Between 100 and 200 µL of PBS-M-DTT (0.7 M D-mannitol [Sigma M9647] in 1X PBS [Gibco 70011-044] with 0.01 M dithiothreitol [DTT, Invitrogen Y00147]; DTT added fresh each day) was added with a p200 pipette to the dried colony. Multiple cuts were made with a feather scalpel in vascularized regions of the colony, including along ampullae as well as regions where vessels existed in the absence of zooids. Small bent-tipped forceps or a pipette tip were used to gently press the tops of the vessels and ampullae to extrude blood from the cut vessels. The PBS-M-DTT with suspended blood cells was removed with a pipette and kept in a microcentrifuge tube on ice.

### 10X Chromium emulsion, library preparation and sequencing

For the budding datasets, cryopreserved samples were slowly thawed on ice, then centrifuged (5 min at 800 g, 4 °C) to wash the DMSO. They were then resuspended in 1% BSA-PBS with 40U/ml RNAsin Plus, filtered with a FlowMi 40 µm cell strainer (Scienceware) to remove the largest aggregates, and stained with 1:300 Draq5 solution (Thermo Fisher, stock 5 mM). They were then sorted by FACS to select only single cells (Supplementary Fig. 1) on a BD Influx cell sorter with 4-way purity by the IPMC cell cytometry platform (Institut de Pharmacologie Moléculaire et Cellulaire – Université Côte d’Azur). Around 75k to 100k cells were collected from the FACS (Aria III, BD Biosciences). The sorted cells were concentrated to around 1000 cells/µl by centrifugation. An aliquot was stained with Hoechst to assess sample quality and count cells using a Countess 3 Automated Cell Counter (Invitrogen). Around 10,000 cells/sample were loaded on 10X Genomics Chromium Single Cell 3’ v3 (10X Genomics 1000123, 1000127, and 1000190), and cDNA libraries were produced following the manufacturer's instructions. The libraries were then sequenced (paired-end) on an Illumina NextSeq2000 with read1 = 28 bp (barcode + UMI) and read2 = 100 bp (mRNA) excluding library index, to a depth of 200-500 million reads. When necessary, resequencing was performed to achieve a sequencing saturation of at least 40%. Library quality was checked with a BioAnalyzer. Individual 10X sample libraries were demultiplexed using Cell Ranger Makefastq v7.0.0 with default settings.

For the circulating cells datasets, blood cells were pooled to generate two scRNAseq libraries: 5 colonies for the sample from phase A1, and 6 colonies for the sample from phase B2 sample. Each cell suspension was filtered through a 40 µm strainer into a microcentrifuge tube which had been coated with 0.5 mL of 10% (v./v.) bovine serum albumin (BSA, Sigma 9048-46-8) in Dulbecco’s phosphate-buffered saline (DPBS, Gibco 14200075). Cells were centrifuged at 400 g for 10 min at 4 °C in a swinging bucket centrifuge (Eppendorf Centrifuge 5810 R). The supernatant was removed, and cells were resuspended in PBS-M-DTT. Cells were counted on a LUNA-FL Automated Fluorescence Cell Counter (Logos Biosystems L20001) using an ultra-low fluorescence slide (Logos Biosystems L12005). The cell viability prior to encapsulation was 79% (A1 sample) and 80% (B2 sample). Cell encapsulation, RT, cDNA amplification, and library preparation were carried out using the Chromium Next GEM Single Cell 3′ v3.1 Kits (10X Genomics 1000123, 1000127, and 1000190). The manufacturer’s guidelines were followed except for one step. To prepare the RT mix prior to cell encapsulation, the manufacturer's protocol instructs adding nuclease-free water, then adding the cell suspension; we added PBS-M instead of nuclease-free water. Libraries were sequenced with Illumina NovaSeq 6000.

### Preparation of the gene count matrix and dimensionality reduction of the single cell datasets

The *Botryllus schlosseri* reference genome was prepared by concatenating the nuclear genome^30^, with the addition of a manually annotated Piwi2 gene (*Boschl.Bs14.g18242* nested) and published mitochondrial genome (sc6a-b isolate, GenBank: HF548551.1, filtered to only the protein coding genes). Reads were quality-checked using FASTQC, and mapped with CellRanger count (v7.1.0 for budding datasets, v6.1.2 for circulating cells datasets). We used CellRanger automatic filtering of empty droplets for the budding samples. For the circulating cells samples, empty droplets were manually filtered by applying a soft threshold at the elbow of the barcode rank plot (retaining barcodes with UMI > 200, Supplementary Fig. 2B).

The single cell analysis was primarily conducted using Seurat (v5.1.0), complemented with selected packages from the Bioconductor (v3.18) and scanPy (v1.9.8) suites. Doublets were detected using scDblFinder (v1.16.0) and filtered out (for the “budding” datasets only). Low-quality cells were removed based on thresholds for low UMI number, low gene counts, and high mitochondrial reads percentage. Additionally, high-UMI cells were excluded from the “circulating cells” datasets to avoid potential multiplets (Supplementary Figs. 2, 3). Initial quality filtering was intentionally performed using permissive dataset-wide thresholds because low-quality cells were not uniformly distributed across the dataset but were enriched in specific populations, particularly cluster 12, which displayed a bimodal distribution of gene counts (Supplementary Fig. 4). Applying stricter global thresholds at the initial stage would have disproportionately removed transcriptionally low-complexity or rare populations, including clusters 5, 8, 20, 21, 26, 30 and 33. Therefore, additional quality filtering was performed after subsetting selected populations, allowing removal of lower-quality cells while preserving biologically relevant low-complexity cell states.

Datasets were analyzed jointly, as separate layers within the same Seurat object, but were not integrated. Normalization and identification of highly variable genes (HVGs) were performed independently for each sample, after which a consensus of HVGs was generated. Following gene expression scaling, PCA was used for dimensionality reduction. A K-Nearest Neighbors (KNN) graph was constructed, and clustering was computed at several resolutions. A Uniform Manifold Approximation and Projection (UMAP) projection was computed with multiple parameter sets to ensure an optimal visualization layout. Differentially expressed genes were calculated for each cluster using Seurat built-in function FindAllMarkers. Cluster-specific markers were chosen based on high average fold changes (avg_log2FC), high proportion of expressing cells (pct.1) within the cluster, and low proportion of expressing cells in other clusters (pct.2). The parameters used for each analysis will be available in the accompanying codes upon publication.

When required, subsets of clusters were reanalyzed from the normalization step to gain high-resolution insights. For the analyses of endostyle cells, clusters 12 and 15 of the full budding dataset were subset and reanalyzed using a similar pipeline. Consistent with the two-step filtering strategy described above, one cluster was identified as low quality (characterized by low UMI and gene counts, and absence of clear marker genes) and was removed before a final round of reanalysis.

For the comparison between the circulating cells dataset and the budding dataset, quality checks and preprocessing were performed on each sample separately, as described above. All samples were analyzed jointly, without integration, using a similar pipeline as described above. The circulating status of each cluster of this combined dataset was assigned based on the proportions of cells from the circulating cells dataset. Clusters with <1% of cells, between 1 and 10%, and >10% from the circulating cells dataset were annotated as “non circulating”, “rarely circulating” or “frequently circulating”, respectively. Correspondence between the cell content of the combined dataset clusters and budding dataset clusters have been identified with Clustree (v0.5.1). Circulating status of the cluster was transferred to the budding dataset clusters accordingly.

For the specific analyses of hematopoietic-lineage hemocytes, cells from the PAGA module “hemocytes” were subsetted and analyzed with Seurat. A new UMAP projection was generated using scanPy by recomputing HVGs, PCA, and constructing a KNN graph. The resulting UMAP coordinates were then transferred back to the Seurat object for downstream analyses.

For the “subset-1” subclustering, we excluded cells assigned to the “hemocytes” and “phagocytes” PAGA modules, and analyzed the remaining subset. This revealed a low-quality cluster (cluster 24 in subclustering of subset-1), which was subsequently removed during the subset-specific filtering step. We were then able to resolve and separate branchial from peribranchial epithelial cells and identify the budlet population more clearly.

For the “subset-2” subclustering, we further refined the analysis by selecting cells from the “subset-1” subclustering, specifically those belonging to early peribranchial epithelium (cluster 2), early branchial epithelium (cluster 9), budlet stalk septum cells (cluster 30), and *Bhf+* cells (cluster 31). This subset was reanalyzed using the same basic pipeline as for the full dataset.

The budlet gene signature score was calculated using UCell (v.2.6.2) based on the expression of *Vim* (*Boschl.Bs2.g1741*), *Runx* (*Boschl.Bs12.g15762-RUNX1.1*), and *Nk4* (*Boschl.Bs6.g8341-NKX2-3*)

Functional annotation was carried out using Eggnog emapper (v2.1.10) with default parameters to assign GO terms to genes. Differentially expressed genes (DEGs) for selected clusters or cluster groups were identified using the FindMarkers function in Seurat, comparing each cluster against the rest of the dataset or subset analysed. GO term enrichment analysis was performed with topGO (v2.54.0): for the body wall muscles, heart and germline clusters, up- and down-regulated genes were used to compute enrichment with the Kolmogorov-Smirnov test; for the putative phagocyte, hyaline amoebocyte, and hemoblast clusters enrichment was calculated on up-regulated genes only using Fisher’s test.

### Potency inference, trajectory inference

To infer cell potency, we used CytoTRACE (v0.3.3^31^) on both the entire dataset and on selected subsets.

For trajectory inference, we applied different complementary approaches. PAGA (Partition-based Graph Abstraction^34^) was used to assess the connectivity between clusters, revealing relationships between transcriptionally similar populations, which can reflect differentiation trajectories. For analysis on the full budding dataset, we removed the lowest quality cells (number of UMI < 400), as they tend to skew the connections. For each PAGA computation, we tested different thresholds to remove weak cluster connections.

To compute RNA velocity, we demultiplexed spliced and unspliced count matrices by running Velocyto (v0.17^33^) on the CellRanger outputs. RNA velocity vectors were then calculated with the scVelo dynamic model (v0.3.2^76^) using a scanPy object converted from the Seurat reference with sceasy (v0.0.7).

In parallel, Monocle3 (v1.3.7^77^) was used to infer putative differentiation trajectories based on transcriptome similarity.

### Immunofluorescence and in situ hybridization by WMISH and HCR

Proliferating cells were revealed by fluorescent immunostaining for phospho-histone 3 (Invitrogen MA5-15220), following the protocol in^78^, and additional phalloidin staining was performed according to^10^.

Gene expression patterns were examined using two methods. Whole mount fluorescent in situhybridization (WMISH), was performed using the protocol described in Langenbacher et al. 2005^36^. For Hybridization Chain Reaction (HCR), probes were designed using Özpolat Lab’s HCR in situ probe generator^79^. Samples were fixed, stored, and dissected, and post-fixed following the WMISH protocol. The remaining steps followed the protocol described in ref. ^80^ from the pre-hybridization stage, with some modifications. Following post-fixation washes, samples were washed twice in 2xSSC and once in 50% 2xSSC - 50% probe hybridization buffer^36^. Samples were pre-hybridized for 4 h at 37 °C in hybridization buffer. The pre-hybridization solution was removed and replaced by 1 µM of probes in hybridization solution, let to hybridize for one night to 3 days at 37 °C. Samples were amplified with 60 nmol/L each hairpin (h1 and h2) conjugated with an Alexa 488 or 647 (B1 or B2 hairpins, Molecular Instruments) and incubated one night to 2 days at 23 °C. Hoechst 33342 staining was performed either during the amplification step, or after amplification washes at 10 µg/mL, for 30 min. As negative controls, we either used no probes, or probes designed for other species, which yielded similar results with low aspecific background and some auto fluorescent cell types. Primers and probes sequences for WMISH and HCR are in Supplementary Data 1.

Samples were mounted in 75% glycerol or Vectashield Plus (Vector) and imaged using a Leica SP8 or Leica Stellaris confocal microscope. Images were processed and analyzed with ImageJ.

### Statistics & reproducibility

Each sample used for the scRNAseq is comprised of pooled buds/budlets (budding dataset) or pooled circulating cells from different colonies (circulating cells dataset), and has only one replicate, except for the budding stage B2, which has two biological replicates. The number of buds/budlets to pool was chosen to have enough singlets for processing with 10X Chromium. No statistical method was used to predetermine sample size. For the FISH/HCR and immunofluorescence experiments, each gene has been studied in at least 3 different samples per stage, in at least 3 different stages. All presented patterns of expression are representative of the samples at that stage.

Statistical analyses for the processing of the scRNA-seq dataset were performed using the established default methods of Seurat and scanpy. Lower-quality cells were filtered out as described in the methods above.

The experiments were not randomized. The Investigators were not blinded to allocation during experiments and outcome assessment.

### Reporting summary

Further information on research design is available in the Nature Portfolio Reporting Summary linked to this article.

## Supplementary information

Supplementary Information

Description of Additional Supplementary Files

Supplementary Data 1

Reporting Summary

Transparent Peer Review file

## Data Availability

The raw and processed single-cell RNA sequencing data generated in this study have been deposited in the Gene Expression Omnibus (GEO) under accession code GSE338836. All scripts and processed data required to reproduce the computational analyses presented in the manuscript are deposited in Zenodo (https://zenodo.org/records/21039528).

## Code availability

The code used for all the analyses in this study is available in Zenodo (https://zenodo.org/records/21039528).
