# Supplementary material for: Multi-stage single-cell atlas of a chordate non-embryonic development unveils dedifferentiating bud founder cells
Source: Nat Commun. 2026 Sep 3;17:10486. doi: 10.1038/s41467-026-76441-3 (PMC13635237; doi:10.1038/s41467-026-76441-3)
Supplement: Supplementary file 1 [file 41467_2026_76441_MOESM1_ESM.pdf]

Supplementary Information

Multi-stage single-cell atlas of a chordate non-embryonic development unveils dedifferentiating bud founder cells

Marie Lebel[1], Solène Marchal[1], Tiphaine Sancerni[1], Sharon Rabiteau[1], Pragati Sharma[1], Tal D. Scully[2], Estelle Balissat[1], Laurel S. Hiebert[3], Anthony W. De Tomaso[3], Allon M. Klein[2], Alexandre Alié[1, †], Stefano Tiozzo[1,†,*]

[1] Laboratoire de Biologie du Développement de Villefranche-sur-Mer (LBDV), UMR7009, CNRS, Sorbonne Université, 06230 Villefranche-sur-Mer, France

[2] Department of Systems Biology, Harvard Medical School, Boston, MA, USA

[3] Molecular, Cellular and Developmental Biology, University of California, Santa Barbara, CA, USA.

[†]Joint supervision

*Corresponding author: stefano.tiozzo@imev-mer.fr

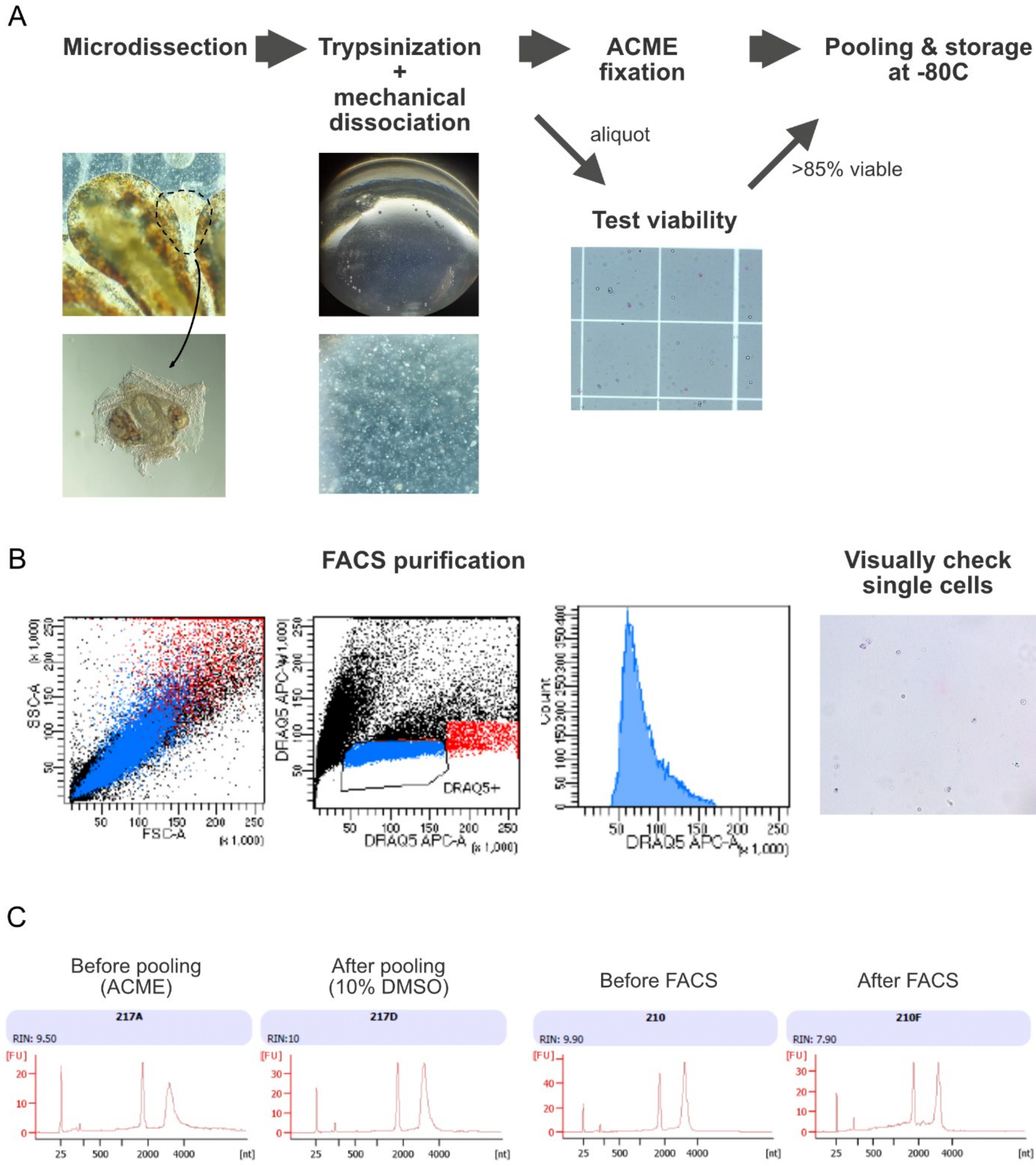

Supplementary Figure 1. Preparation of the samples for the full budding scRNAseq dataset. (A) Main steps of the sample preparation shown for one sample, from dissection to preservation at -80°C. **(B)** Example of FACS gating based on DNA content (stained with Draq5) to remove debris and cell aggregates (sample ML231-B2 shown), the same gating/sorting strategy has been follow for each sample. **(C)** Bio-Analyzer chromatograms representatives of the different samples dissociated and tested for RNA quality, both before and after cryopreservation, and before and after FACS.

Samples	Phase	Number of buds pooled	Estimated number of singlets before fixation	Average number of singlets/bud	Average viability (%)	Pooled dissoci ations
**ML231-B2**	B2	53	905,400	17,083	93,8	
**ML232-B2**	B2	65	1,234,240	18,988	93,6	
**ML232-B1**	B1	83	509,600	6,140	96,0	from 2 different days
**ML232-A2**	A2	156	686,880	4,403	91,5	
**ML245-A1**	A1	209	506,160	2,422	90,3	from 2 different days
**ML245-D**	D	337	694,000	2,059	89,38	from 4 different days
**ML263-C2**	C2	50	1,193,840	23,876	94,98	
**ML275-C1**	C1	82	2,161,440	26,359	95,94	from 2 different days

Supplementary Table 1: Summarized dissociation metrics for each sample in the budding dataset. The sample ID is the same as the one used in the Seurat object. Unless otherwise mentioned in the comment column, samples were obtained by pooling dissociation batches from the same day and colony. The number of singlets and viability were estimated on a 10µl aliquot of each dissociation batch (100-150µl total, 10-12 buds/budlets each) before fixation.

Sample	Estimated Number of Cells	Mean Reads per Cell	Median Genes per Cell	Fraction Reads in Cells	Confidently Mapped to the Transcriptome	Number of Reads
**ML231-B2**	7,494	33,224	1,031	50.4%	43.7%	248,979,225
**ML232-B2**	9,094	29,972	1,602	69.8%	64.8%	272,561,325
**ML232-B1**	9,087	29,822	1,667	70.2%	63.4%	270,990,370
**ML232-A2**	7,052	30,717	1,621	71.3%	64.6%	216,616,437
**ML245-A1**	11,001	26,380	1,609	75.0%	55.1%	290,211,004
**ML245-D**	21,648	22,588	1,621	73.3%	59.1%	488,974,966
**ML263-C2**	4,346	92,592	1,292	46.5%	62.2%	402,405,588
**ML275-C1**	8,535	28,833	1,278	56.2%	60.1%	246,088,402

Supplementary Table 2. Summary of mapping and filtering by CellRanger. The sample IDs are the same as the ones used in the Seurat object. The number of cells is estimated by filtering out empty droplets with a computed threshold at the elbow of the barcode rank plot.

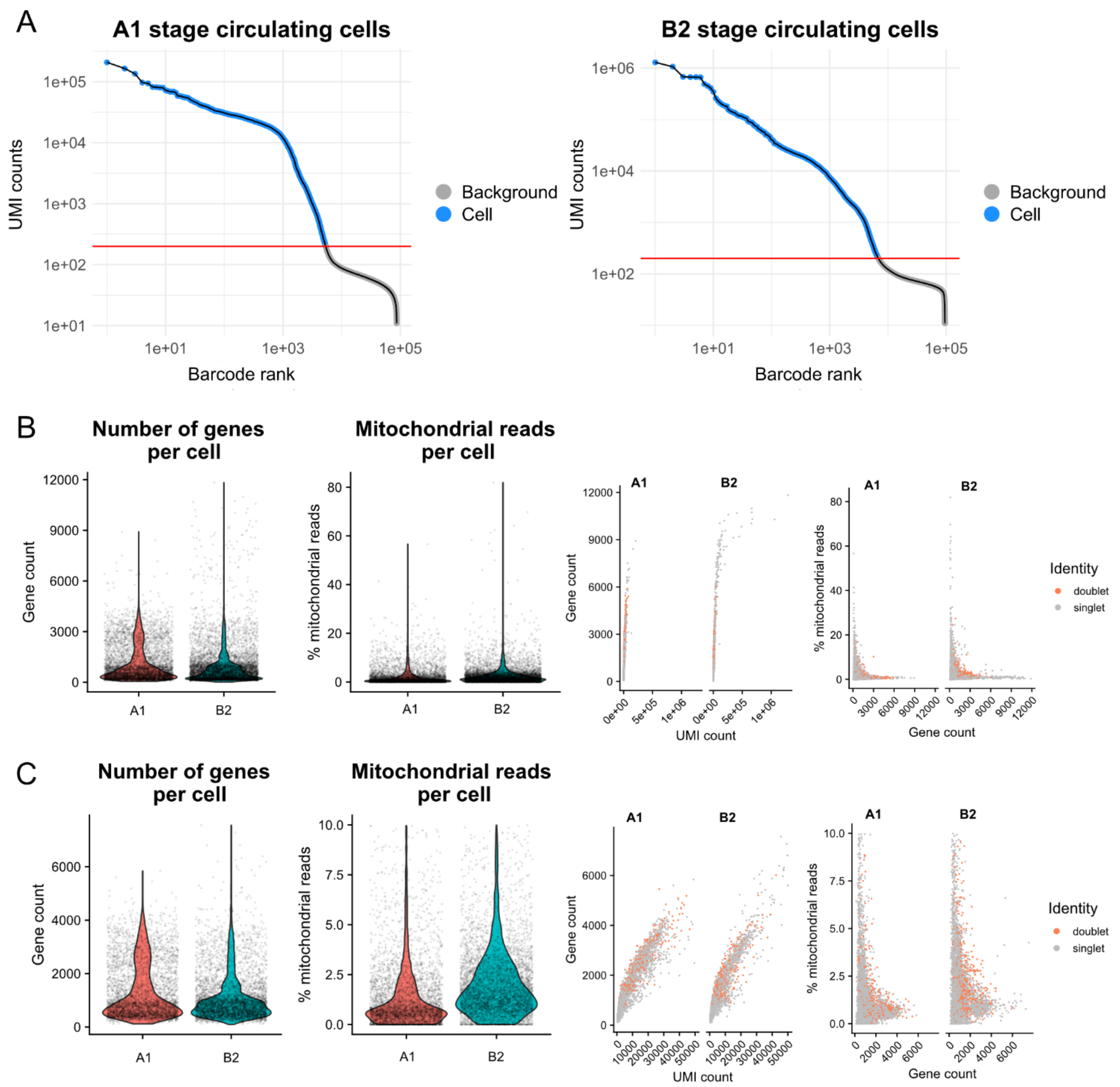

Supplementary Figure 2. Distribution of the quality metrics in each circulating cell sample pre- and post-filtering. (A) Barcode rank plots of the two circulating cell samples showing the threshold to filter out inferred empty droplets. Red line = 200 UMIs threshold. **(B-C)** Distribution of quality control metrics **(B)** pre- and **(C)** post-filtration. Cells were kept if the number of detected UMIs (nCount_RNA) was between 500 and 50000, and the percentage of UMIs from mitochondrial genes (percent.mt) was under 10. We decided against filtering out the doublets in these samples because, contrary to expectations, the "doublets" detected by *scDblFinder* are evenly distributed, and not concentrated toward high UMI counts/high gene counts. In addition, due to the nature of the samples (circulating cells), only rare multiplets are expected.

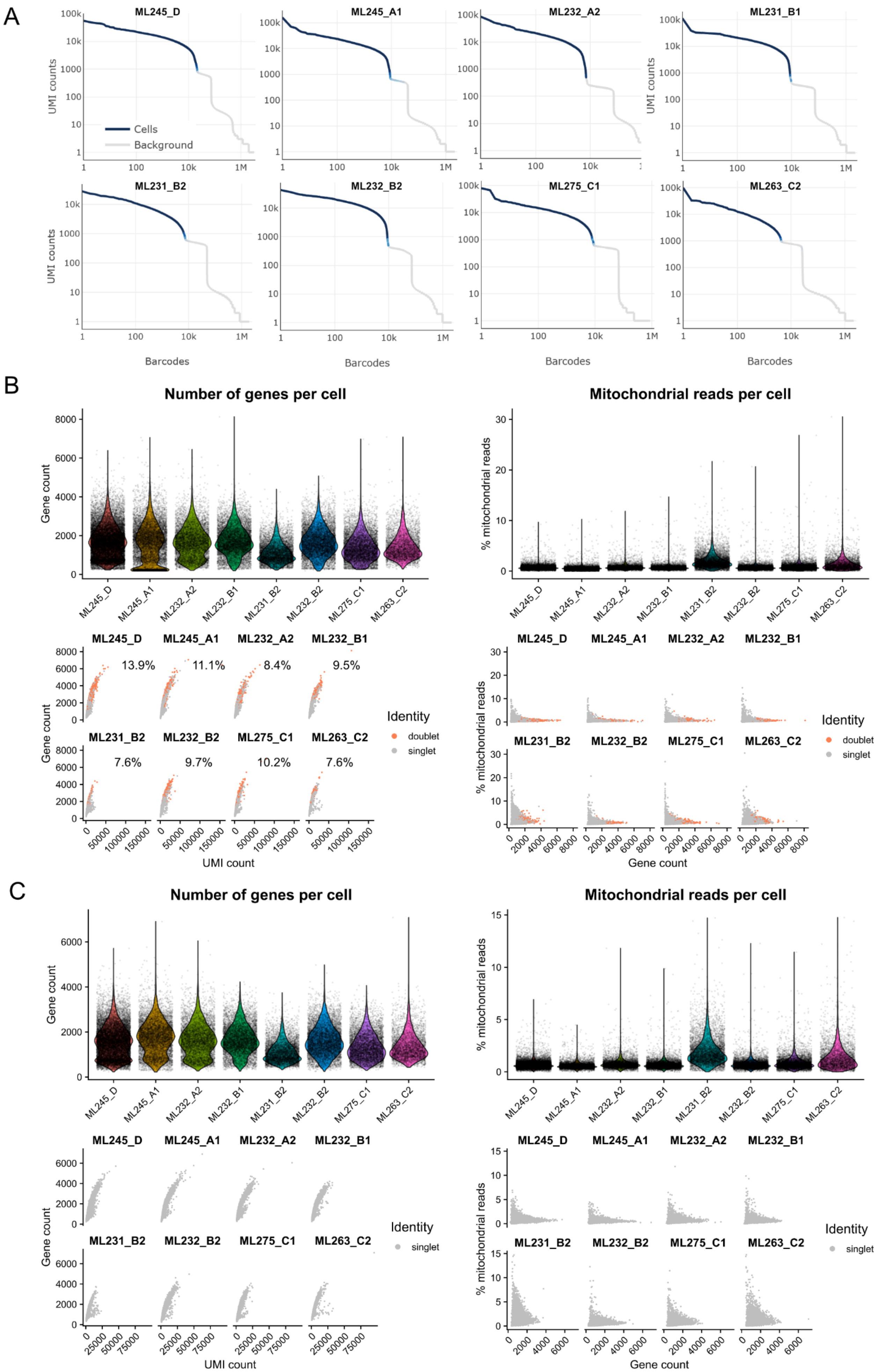

A
ML245_D
ML245_A1
ML232_A2
ML231_B1
ML231_B2
ML232_B2
ML275_C1
ML263_C2
UMI counts
Barcodes
Cells
Background
B
Number of genes per cell
Gene count
Mitochondrial reads per cell
% mitochondrial reads
13.9%
11.1%
8.4%
9.5%
7.6%
9.7%
10.2%
7.6%
Identity
doublet
singlet
UMI count
Gene count
C
Number of genes per cell
Gene count
Mitochondrial reads per cell
% mitochondrial reads
Identity
singlet
UMI count
Gene count

Supplementary Figure 3. Distribution of the quality metrics in each budding sample pre- and post-filtering. (A) Barcode rank plots of the eight budding samples and filtering from CellRanger. **(B-C)** Distribution of quality control metrics **(B)** pre- and **(C)** post-filtration. Cells were kept if the number of detected UMI (nCount_RNA) was higher than 750, the percentage of UMI from mitochondrial genes (percent.mt) under 15%, and either the gene count (nFeature_RNA) was over 400 or the percent.mt under 5%. Rate of doublets detected by scDbFinder are highlighted in B (left figure).

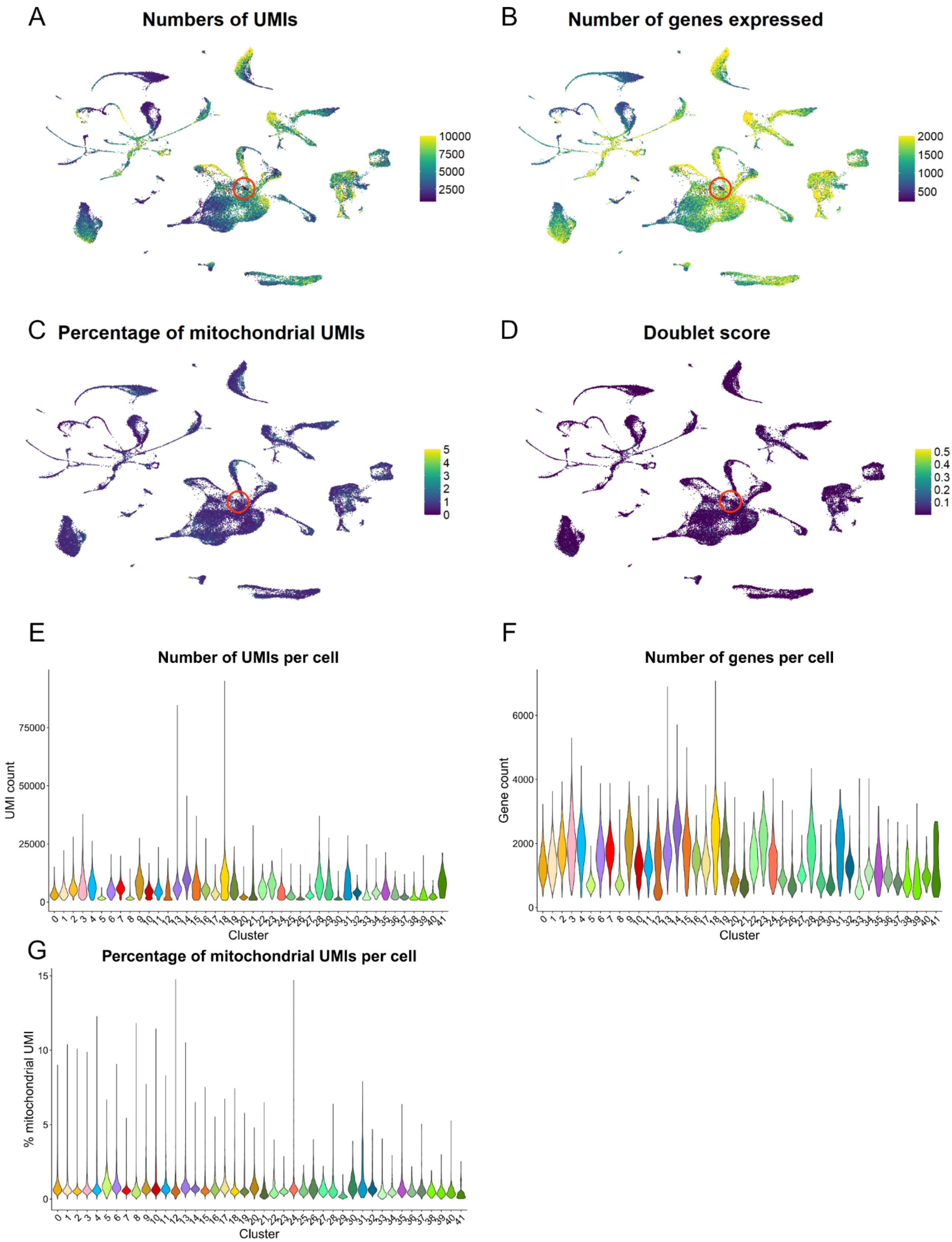

A
Numbers of UMIs
10000
7500
5000
2500
B
Number of genes expressed
2000
1500
1000
500
C
Percentage of mitochondrial UMIs
5
4
3
2
1
0
D
Doublet score
0.5
0.4
0.3
0.2
0.1
E
Number of UMIs per cell
UMI count
75000
50000
25000
0
Cluster
F
Number of genes per cell
Gene count
6000
4000
2000
0
Cluster
G
Percentage of mitochondrial UMIs per cell
% mitochondrial UMI
15
10
5
0
Cluster

Supplementary Figure 4. Quality metrics of the full budding dataset. (A-D) Distribution across the UMAP and **(E-G)** violin plots of quality metrics per cluster. Most clusters show a good distribution of the quality metrics. A portion of cluster 12 might be cells of lower quality, as it has both very low UMI numbers and gene counts (dashed red circle in A, B). **(A, E)** Total number of RNA molecules (UMIs) per cell (the UMAP color max was set at 10,000 for readability). **(B, F)** Genes counts per cell (the UMAP color max was set at 2,000 for readability). **(C, G)** Percentage of mitochondrial RNA molecules per cell (the UMAP color max was set at 5% for readability). **(D)** Doublet score assigned by *scDblFinder*. All cells correspond to singlets. No high doublet score cluster is visible, suggesting good filtering.

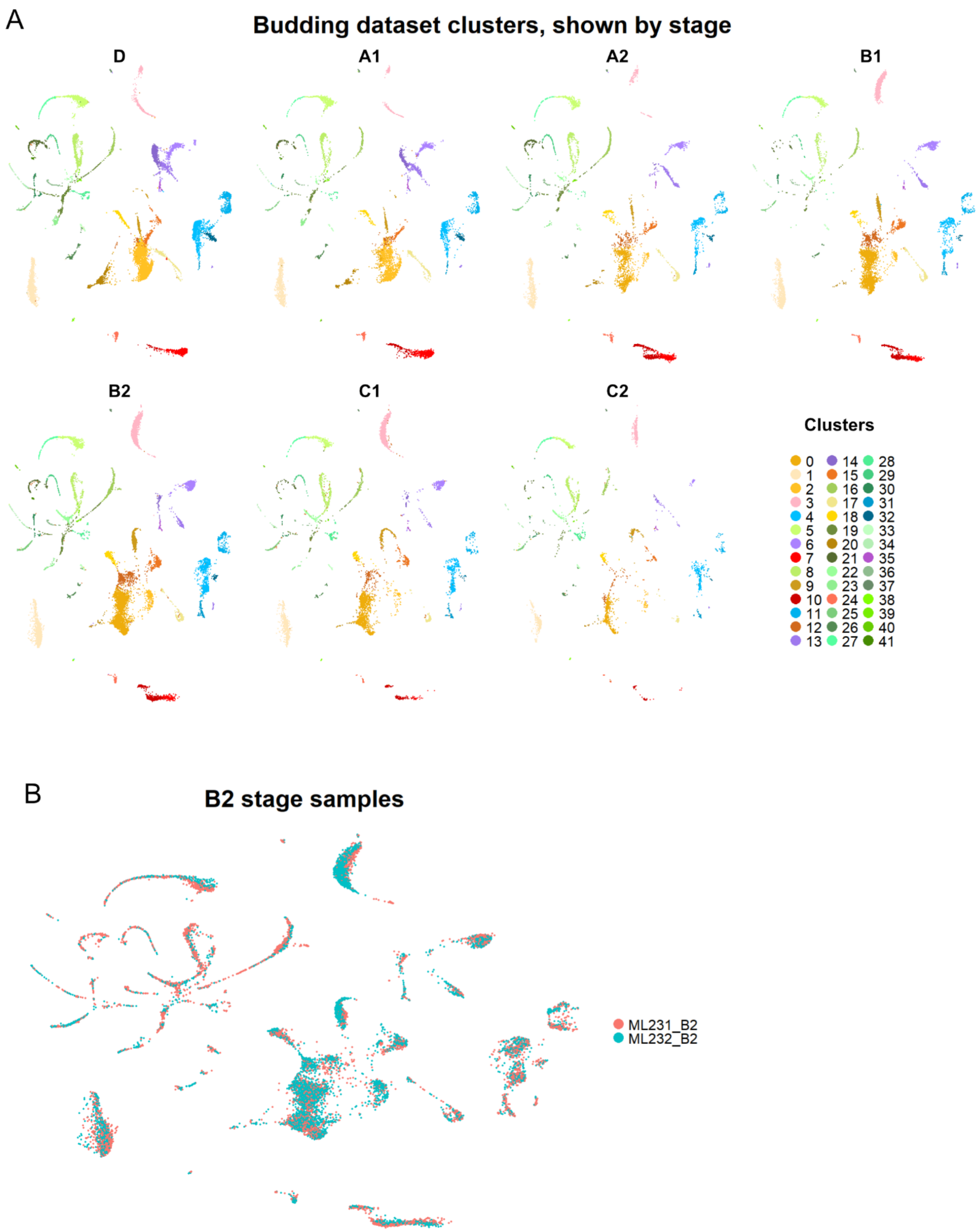

Supplementary Figure 5. Distribution of the stages and of the two B2 stage replicates across the budding dataset. (A) UMAP projection of the full budding dataset split by stage: D, A1, A2, B1, B2, C1 and C2. **(B)** UMAP projection with only the two B2 samples replicates. The B2 samples (prepared and sequenced at different times on the same clonal colony) are superposed almost perfectly on the UMAP, suggesting a low batch effect in our dataset.

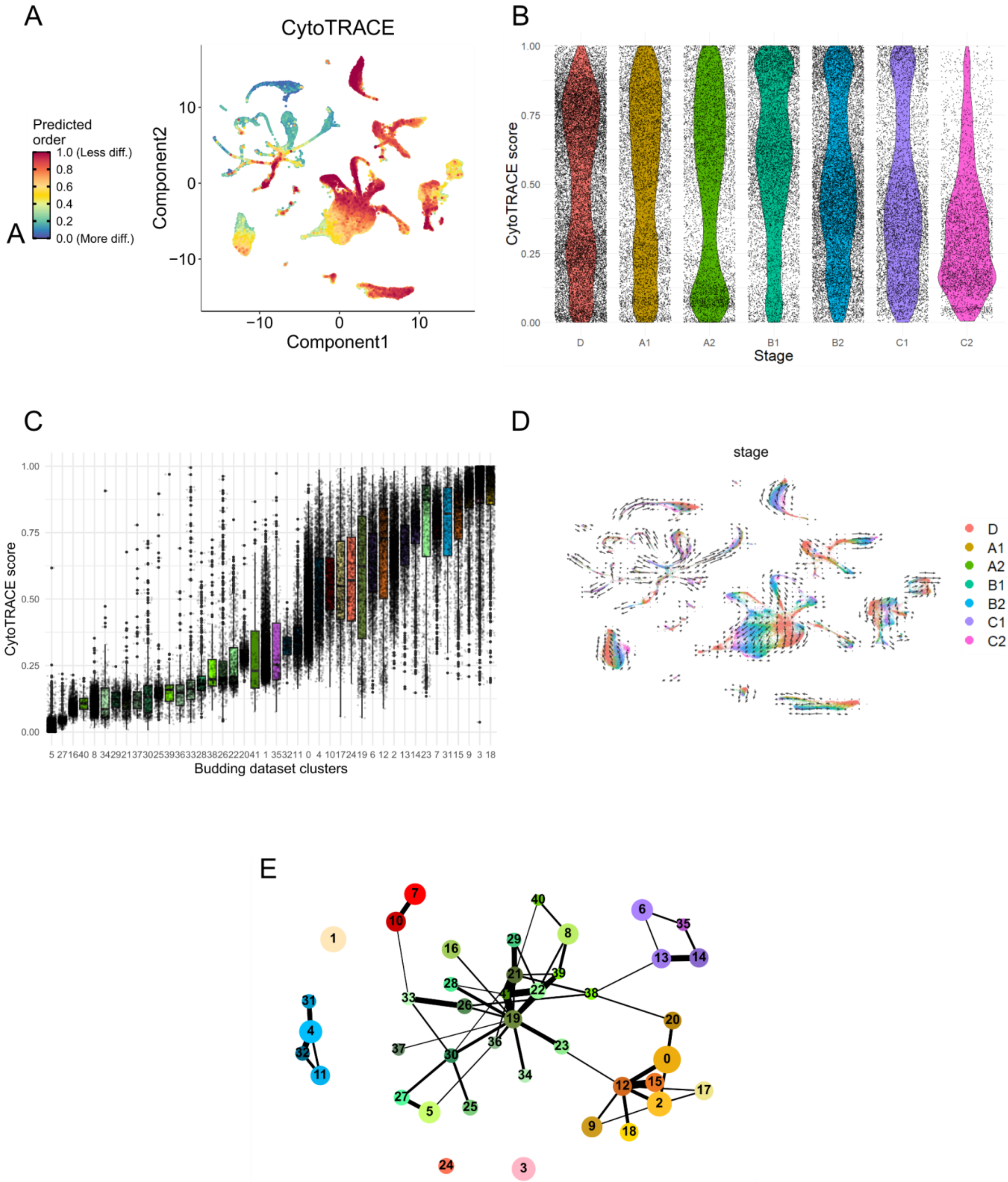

Supplementary Figure 6. CytoTRACE, RNA velocity and PAGA analyses of the full budding dataset. (A-C) CytoTRACE score, **(A)** Distribution of the score over the full budding dataset UMAP, **(B)** Score distribution by stage (n=66,227 cells, with 3969-18331 cells per stage), **(C)** Score distribution by cluster (n=66,227 cells, with 93-7482 cells per cluster), the boxplot show the median (line in the box), the first and third quartiles (the hinges of the box) and the largest/smallest value no further than 1.5 * inter-quartile range from the corresponding hinge (the whiskers), as well as any outlier beyond that

point as bold points. All data are represented as small points. **(D)** RNA velocity projected on the UMAP, with colors representing stage. **(E)** Abstracted graph (PAGA) of the full budding dataset clusters, with PAGA threshold=0.1, showing some low strength connections between the “hemocytes” module (green) and the other modules.

**Published gene name**	**Gene id and name in our dataset**	**Tissue/Cell type from budlet stage C1**	**References**
Vasa	*Boschl.Bs4.g5801-DDX4*	Germline	[36]
Piwi	*Boschl.Bs5.g6728-PIWIL1.1*	Germline	[35]
Myh3	*Boschl.Bs15.g19623-Myh6*	Body wall muscle	[21]
Mrf	*Boschl.Bs4.g5757-MYOD1*	Cells of the mantle (possibly muscle precursors)	[21]
Ebf	*Boschl.Bs10.g14018-EBF1*	Body wall muscle precursors and developing nervous system	[21]
Myh2	*Boschl.Bs8.g10861-MYH3.1*	Heart	[21]
FoxF	*Boschl.Bs7.g9951-FOXF1.1*	Epidermis, Dorsal branchial, Heart	[21]
Tbx1/10	*Boschl.Bs2.g1884-TBX1.1*	Mesenchymal cells, branchial domain, cerebral ganglion region, and developing heart	[21]
Nk4	*Boschl.Bs6.g8341-NKX2-3*	Left branchial and left peribranchial region, developing myocardium	[21]
Gata-a	*Boschl.Bs2.g2024-GATA5.1*	Gut primordium and developing heart	[9]
Tgf-β-f	*Boschl.Bs8.g10511*	Follicular cells /Support cells	[36]

Gata-b	*Boschl.Bs13.g17419-GATA3.1*	External peribranchial and posterior branchial	[9]
FoxA1	*Boschl.Bs6.g7874-FOXA1*	Gut rudiment (esp. esophagus), Endostyle rudiment, Stigmata	[9]*
FoxA2	*Boschl.Bs8.g11479-FOXA2*	Gut	[9]*
Zic-r.a	*Boschl.Bs13.g17465*	Developing central nervous system	[20]
Pax3/7	*Boschl.Bs8.g10830-PAX3.1*	Developing and mature central nervous system	[20]
Tbx2/3	*Boschl.Bs9.g12221-TBX2.1*	Dorsal tube, Stomach, Branchial flanking the endostyle	[9]
SoxB1	*Boschl.Bs11.g14812-SOX3.1*	Stigmata, Endostyle, Neural Gland, Esophagus, Germline, Heart, and some hemoblasts	[55]

Supplementary Table 3. Published pattern of expression of genes in *B. schlosseri,* with their ID in our dataset and publication of origin.

*The patterns of expression of FoxA1 and FoxA2 observed in the budding dataset are inverted compared to the description of Ricci et al. 2016[9]

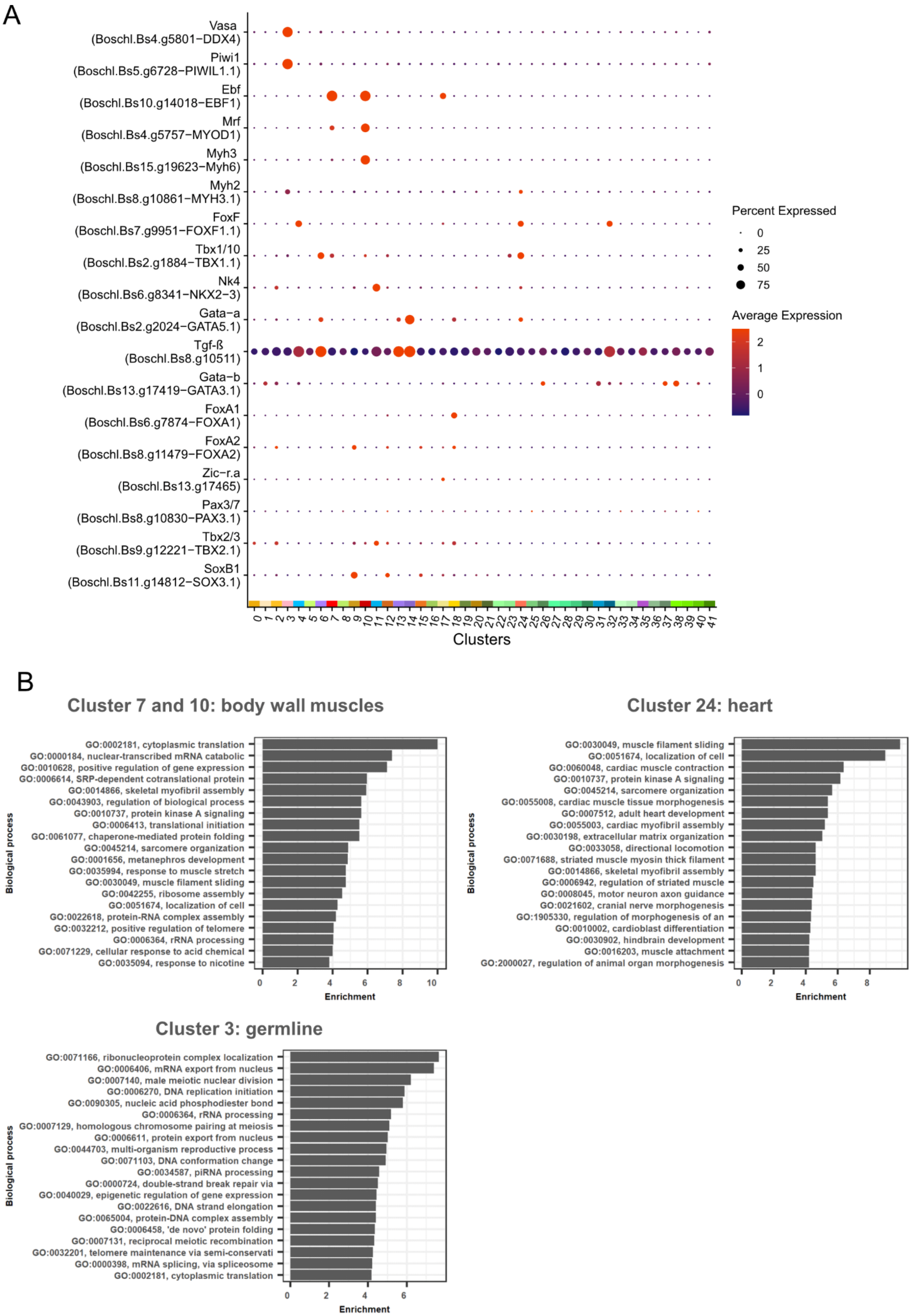
A
Vasa (Boschl.Bs4.g5801−DDX4)
Piwi1 (Boschl.Bs5.g6728−PIWIL1.1)
Ebf (Boschl.Bs10.g14018−EBF1)
Mrf (Boschl.Bs4.g5757−MYOD1)
Myh3 (Boschl.Bs15.g19623−Myh6)
Myh2 (Boschl.Bs8.g10861−MYH3.1)
FoxF (Boschl.Bs7.g9951−FOXF1.1)
Tbx1/10 (Boschl.Bs2.g1884−TBX1.1)
Nk4 (Boschl.Bs6.g8341−NKX2−3)
Gata−a (Boschl.Bs2.g2024−GATA5.1)
Tgf-ß (Boschl.Bs8.g10511)
Gata−b (Boschl.Bs13.g17419−GATA3.1)
FoxA1 (Boschl.Bs6.g7874−FOXA1)
FoxA2 (Boschl.Bs8.g11479−FOXA2)
Zic−r.a (Boschl.Bs13.g17465)
Pax3/7 (Boschl.Bs8.g10830−PAX3.1)
Tbx2/3 (Boschl.Bs9.g12221−TBX2.1)
SoxB1 (Boschl.Bs11.g14812−SOX3.1)
Clusters
Percent Expressed
0
25
50
75
Average Expression
2
1
0
B
Cluster 7 and 10: body wall muscles
GO:0002181, cytoplasmic translation
GO:0000184, nuclear-transcribed mRNA catabolic
GO:0010628, positive regulation of gene expression
GO:0006614, SRP-dependent cotranslational protein
GO:0014866, skeletal myofibril assembly
GO:0043903, regulation of biological process
GO:0010737, protein kinase A signaling
GO:0006413, translational initiation
GO:0061077, chaperone-mediated protein folding
GO:0045214, sarcomere organization
GO:0001656, metanephros development
GO:0035994, response to muscle stretch
GO:0030049, muscle filament sliding
GO:0042255, ribosome assembly
GO:0051674, localization of cell
GO:0022618, protein-RNA complex assembly
GO:0032212, positive regulation of telomere
GO:0006364, rRNA processing
GO:0071229, cellular response to acid chemical
GO:0035094, response to nicotine
Biological process
Enrichment
Cluster 24: heart
GO:0030049, muscle filament sliding
GO:0051674, localization of cell
GO:0060048, cardiac muscle contraction
GO:0010737, protein kinase A signaling
GO:0045214, sarcomere organization
GO:0055008, cardiac muscle tissue morphogenesis
GO:0007512, adult heart development
GO:0055003, cardiac myofibril assembly
GO:0030198, extracellular matrix organization
GO:0033058, directional locomotion
GO:0071688, striated muscle myosin thick filament
GO:0014866, skeletal myofibril assembly
GO:0006942, regulation of striated muscle
GO:0008045, motor neuron axon guidance
GO:0021602, cranial nerve morphogenesis
GO:1905330, regulation of morphogenesis of an
GO:0010002, cardioblast differentiation
GO:0030902, hindbrain development
GO:0016203, muscle attachment
GO:2000027, regulation of animal organ morphogenesis
Biological process
Enrichment
Cluster 3: germline
GO:0071166, ribonucleoprotein complex localization
GO:0006406, mRNA export from nucleus
GO:0007140, male meiotic nuclear division
GO:0006270, DNA replication initiation
GO:0090305, nucleic acid phosphodiester bond
GO:0006364, rRNA processing
GO:0007129, homologous chromosome pairing at meiosis
GO:0006611, protein export from nucleus
GO:0044703, multi-organism reproductive process
GO:0071103, DNA conformation change
GO:0034587, piRNA processing
GO:0000724, double-strand break repair via
GO:0040029, epigenetic regulation of gene expression
GO:0022616, DNA strand elongation
GO:0065004, protein-DNA complex assembly
GO:0006458, 'de novo' protein folding
GO:0007131, reciprocal meiotic recombination
GO:0032201, telomere maintenance via semi-conservati
GO:0000398, mRNA splicing, via spliceosome
GO:0002181, cytoplasmic translation
Biological process
Enrichment

Supplementary Figure 7. Annotation of clusters identity from published expression patterns and GO term enrichment. (A) Scaled expression of genes with described expression patterns in *B. schlosseri* and other tunicates. The genes correspond to the ones presented in Supplementary Tab. 3. **(B)** GO term enrichment analysis of the body wall muscles, heart, and germline clusters.

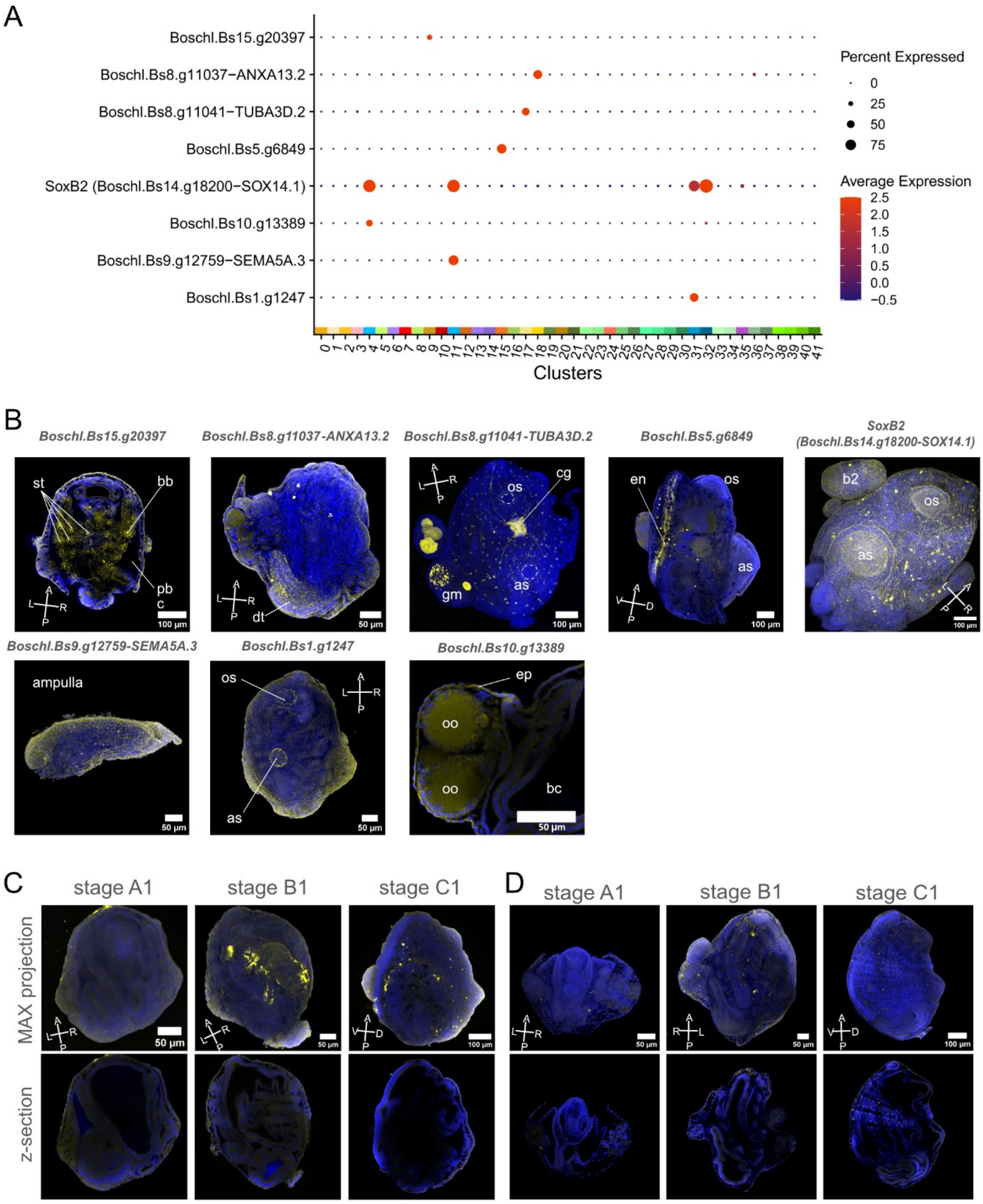

Supplementary Figure 8. Additional gene expression patterns to annotate the clusters, and negative controls. (A) Scaled expression of additional marker genes specific to a tissue type, in the different clusters of the full budding dataset and **(B)** corresponding expression patterns observed by WMISH in primary buds or in ampullae. **(C-D)** Negative controls for **(C)** WMISH and **(D)** HCR. Some mesenchymal cells with specific morphology are fluorescent in the FITC (C) or Alexa-647 (D) emission window. Additionally, surface staining is sometimes observed in WMISH in remnants of the tunic. All

presented patterns of expression have been observed in at least 3 samples of the same stage. Probe is in yellow, DAPI or Hoechst staining is in blue. st: stigmata; bb: branchial basket; pbc: peribranchial chamber; dt: digestive tract; os: oral siphon; as: atrial siphon; cg: cerebral ganglion; gm: gonadal mass; en: endostyle; oo: oocyte; ep: epidermis; bc: branchial chamber, b2: budlet.

**Gene name**	**ID**	**Expression in the endostyle zone**								**Other Branchial expression zones**	**Source**
		**1**	**2**	**3**	**4**	**5**	**6**	**7**	**8**		
Ttf1/ Nkx2.1	*Boschl.Bs11.g14583*	X	X	X	X	X	X				This study and *C. robusta* [40,41,84]
	Boschl.Bs5.g6849	X	X	X	X	X	x				This study
Runx	*Boschl.Bs12.g15762*	x		x		x					This study
FoxA-a/ FoxA2	*Boschl.Bs8.g11479-FOXA2*	c		C					C	Extremities of stigmata	*Ciona sp.*[45]
Vim	*Boschl.Bs2.g1741*		x		x						This study
Bs-Vwfl2	*Boschl.Bs11.g14818*		X								*B. schlosseri* [36]
Ci-Vwfl2	*Boschl.Bs11.g14815*		C								*C. robusta* [40]
Ci-Vwfl1	*Boschl.Bs11.g14818*				C						*C. robusta* [40]
Ends10	*Boschl.Bs5.g6637-TRIP11.1*				C						*Ciona sp.* [41]
Ends11	*Boschl.Bs12.g16049*		c	C	c	c					*Ciona sp.* [41]
Ends1	*Boschl.Bs2.g2666*						C				*C. robusta* [40,44]
FoxD	*Boschl.Bs12.g16697*						X				This study and *C. robusta* [40]
FoxQ	*Boschl.Bs15.g19643*	c		C	c	X		X		partial	This study and *C. robusta* [40,84]

Pax 2/5/8a	*Boschl.Bs16.g20739*			c		X		X			This study + *C. robusta* [40,45]
FoxE	*Boschl.Bs16.g21212*					C	C	C			*C. robusta*
Nk4	*Boschl.Bs6.g8341*							X			This study and *Ciona sp.* [46]
Tbx1/10	*Boschl.Bs2.g1884*							dorsal and flanking the endostyle			*B. schlosseri* [21]
Tbx2/3	*Boschl.Bs9.g12221-TBX2.1*							flanking the endostyle rudiment			*B. schlosseri* [9]
Tpo	*Boschl.Bs12.g16197*							C			*C. robusta*
Duox	*Boschl.Bs6.g8282-DUOX2, Boschl.Bs6.g8283-DUOXA1.1*							C			*Ciona sp.* [46]
Pax1/9	*Boschl.Bs6.g8552*								X	branchial	This study

Supplementary Table 4. Published expression patterns in the endostyle zones and other branchial tissues. Genes used as reference for identifying the clusters in Figure 3D. X/x: expression pattern reported in *B. schlosseri*, C/c: expression reported in *Ciona* but not observed in this study. Uppercase: strong expression, lowercase: low/partial expression.

A

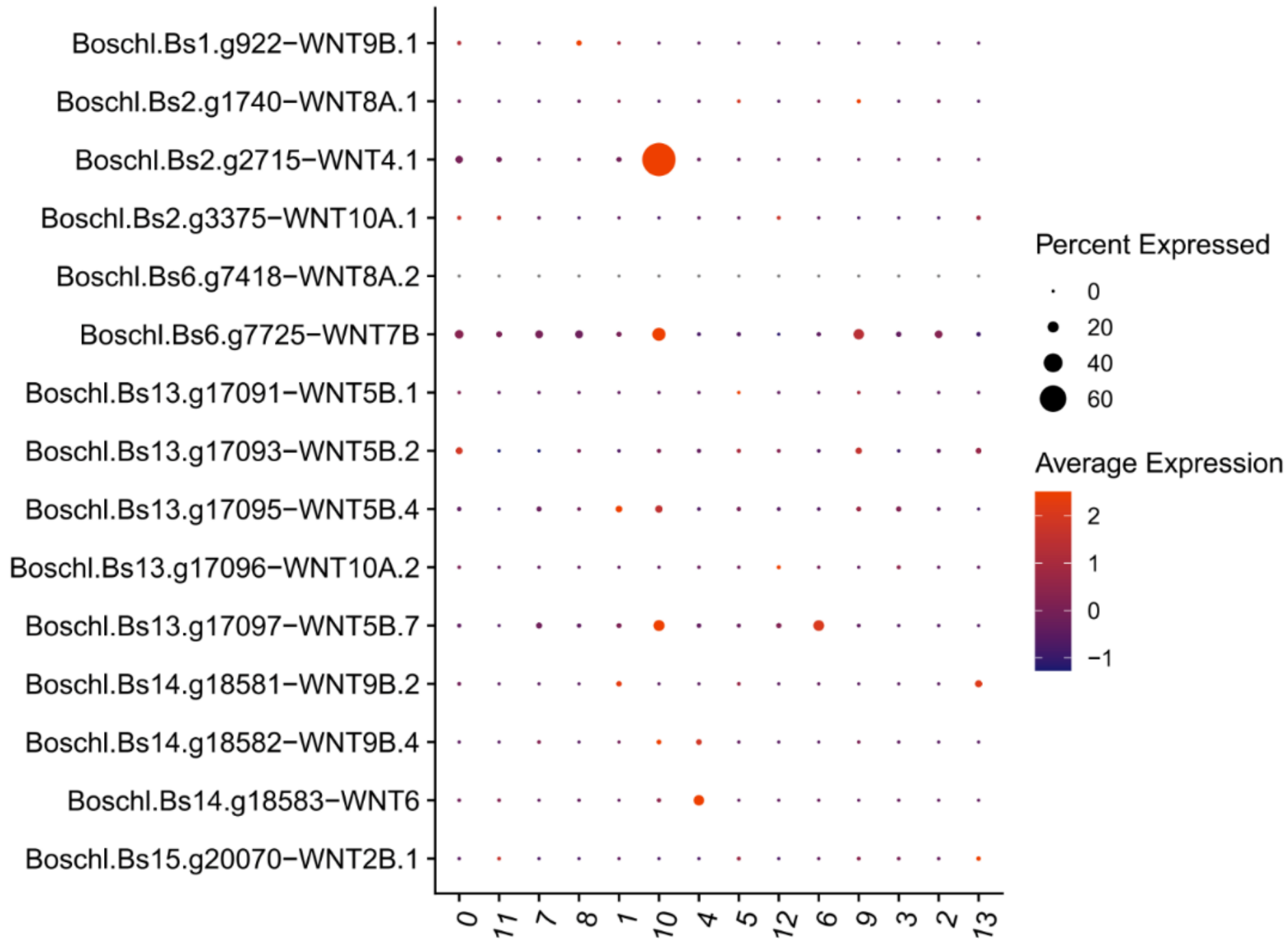

B

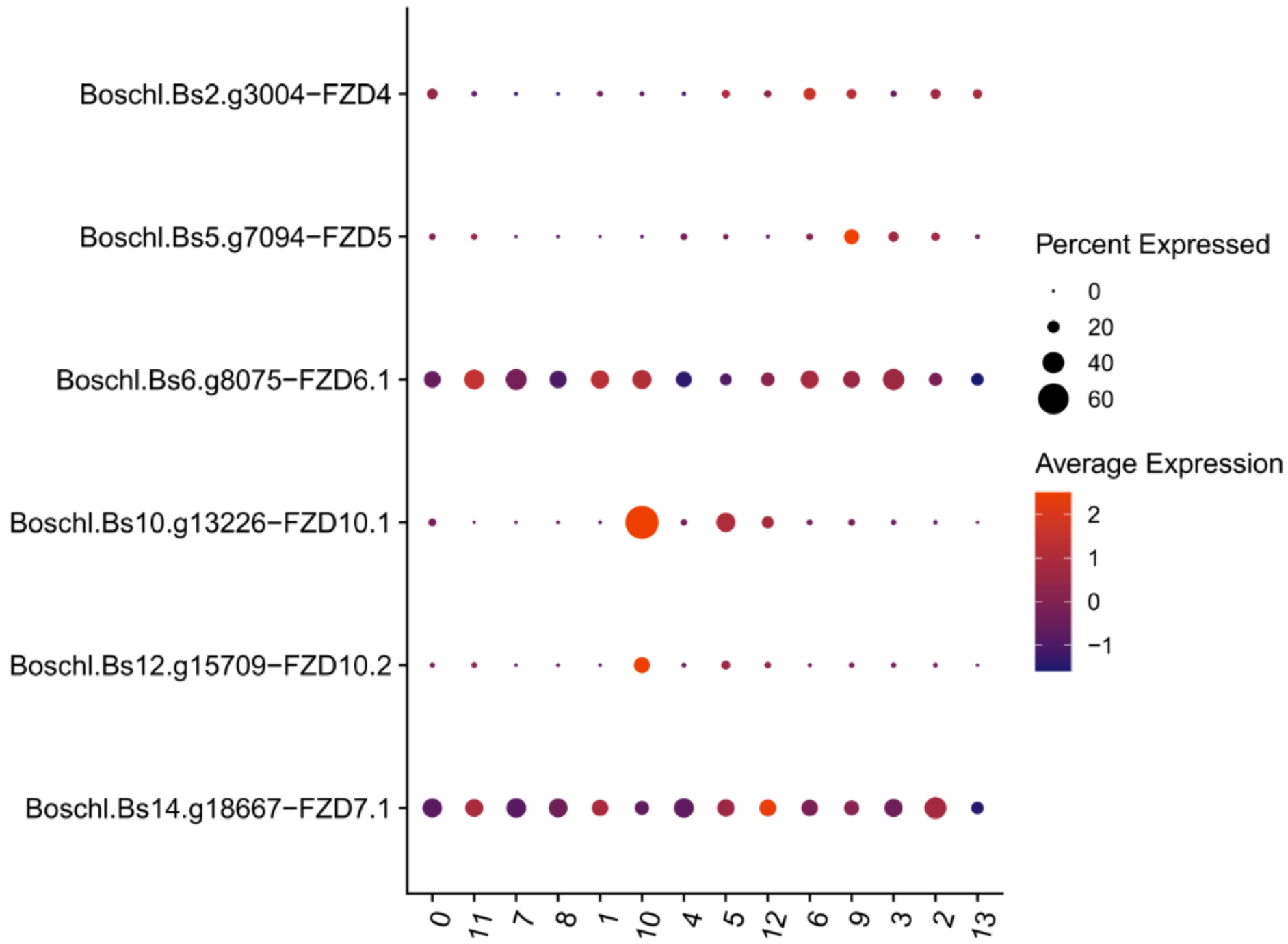

Supplementary Figure 9. Scaled expression of *Wnt* and *Fzd* genes in the endostyle subset. (A-B) The presented *Wnt* (A) and *Fzd* (B) genes were selected from the functional annotation. Clustering corresponds to the endostyle subclusters presented in Figure 3A.

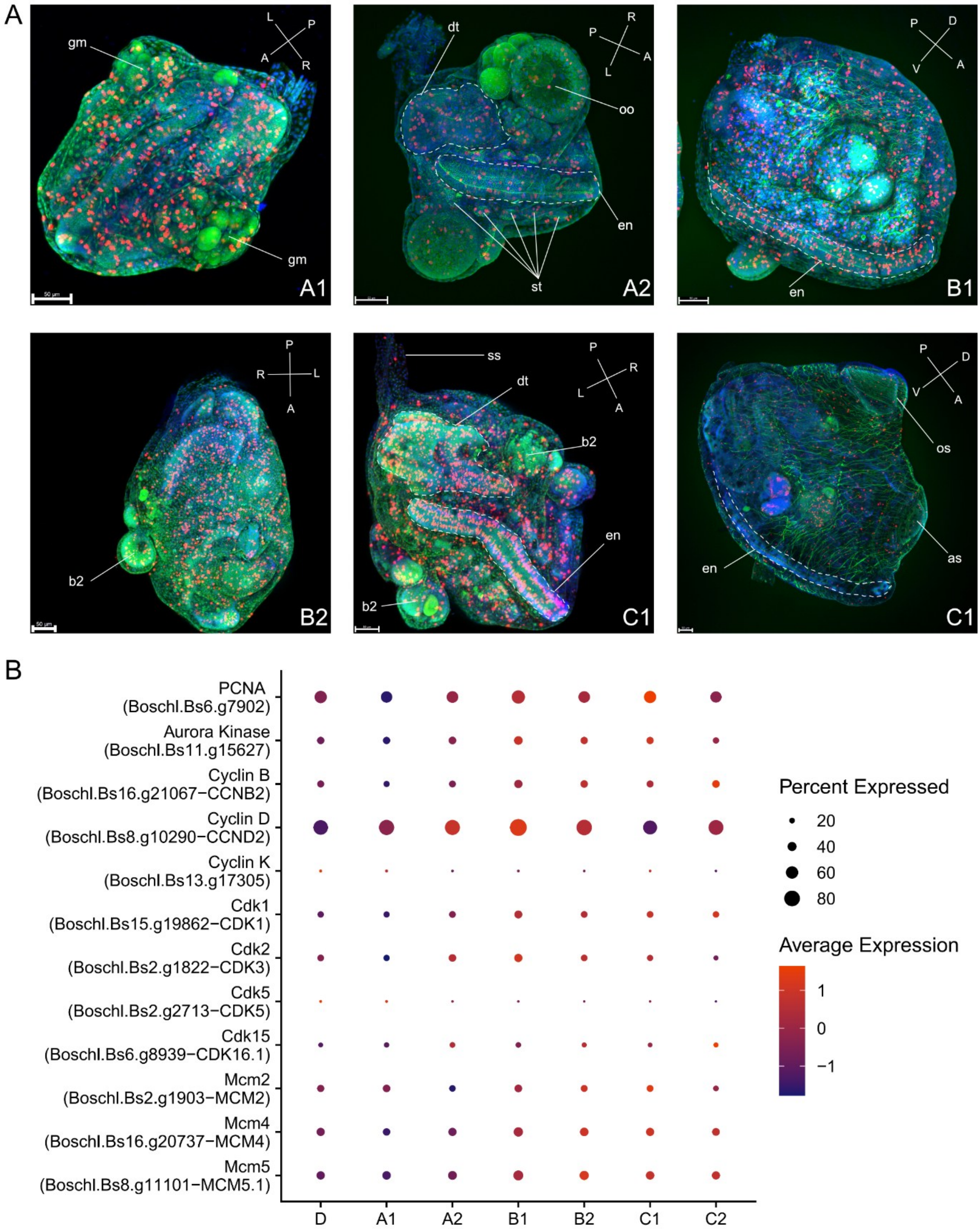

Supplementary Figure 10. Proliferative dynamic in the budding dataset. (A) Confocal pictures of buds and budlets stained with an antibody against phospho-histone H3 (red), phalloidin (green) and Hoechst (blue). Orientation is shown on the top right, scale bar is 50µm. **(B)** Scaled expression of proliferation-associated genes across budding stages in the budding dataset, highlighting widespread mitotic activity during bud and budlet development. gm: gonadal mass; dt: digestive tract; st: stigmata; en: endostyle; ss: stalk; b2: budlet; os: oral siphon; as: atrial siphon.

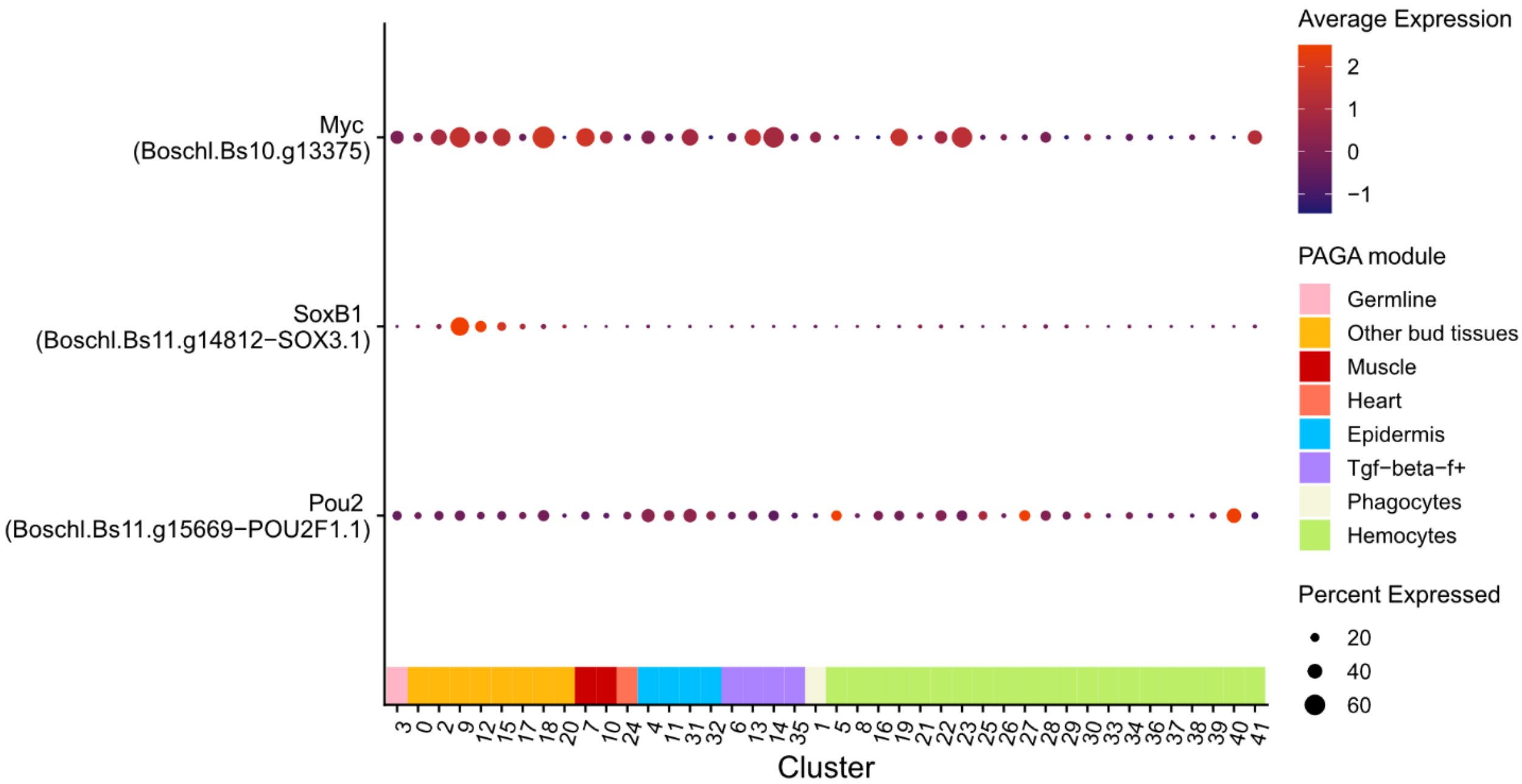

Supplementary Figure 11. Expression of Yamanaka factors-related genes. Scaled expression of Yamanaka factors-related genes in the clusters of the full budding dataset

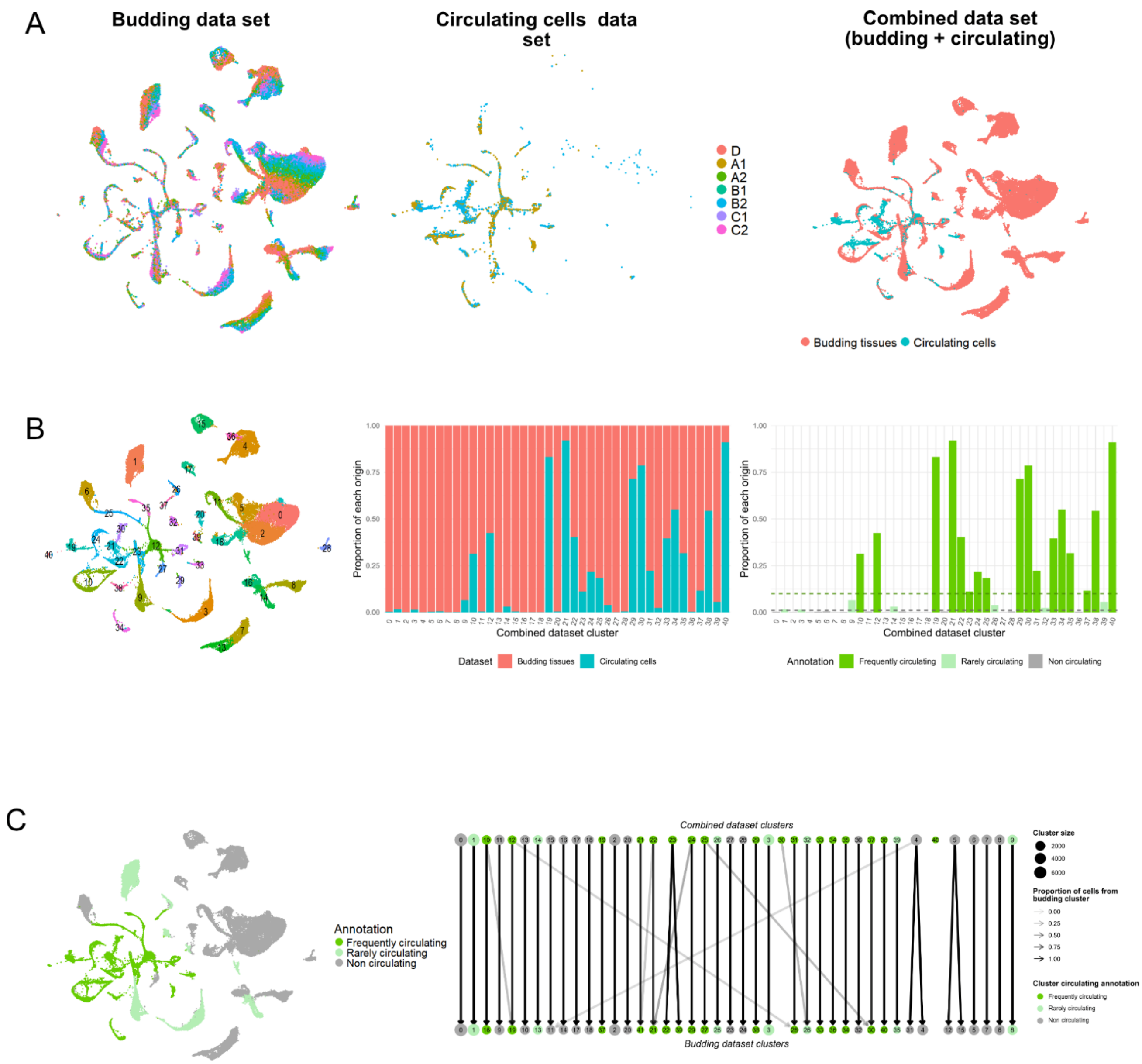

Supplementary Figure 12. Comparison of budding and circulating cells datasets. (A) UMAP projections of the seven budding stages (left) and two circulating datasets (center) labeled by stage, and their overlap (right). **(B)** UMAP projection of the combined dataset clusters (left). Proportions of cells coming from each dataset in each cluster (center), and corresponding annotation on circulating status (right). The 1% and 10% thresholds between circulating statuses are highlighted with dashed lines. **(C)** UMAP projection of the combined dataset (left) labeled by circulating status and correspondence of cluster numbers between combined dataset and budding dataset (right).

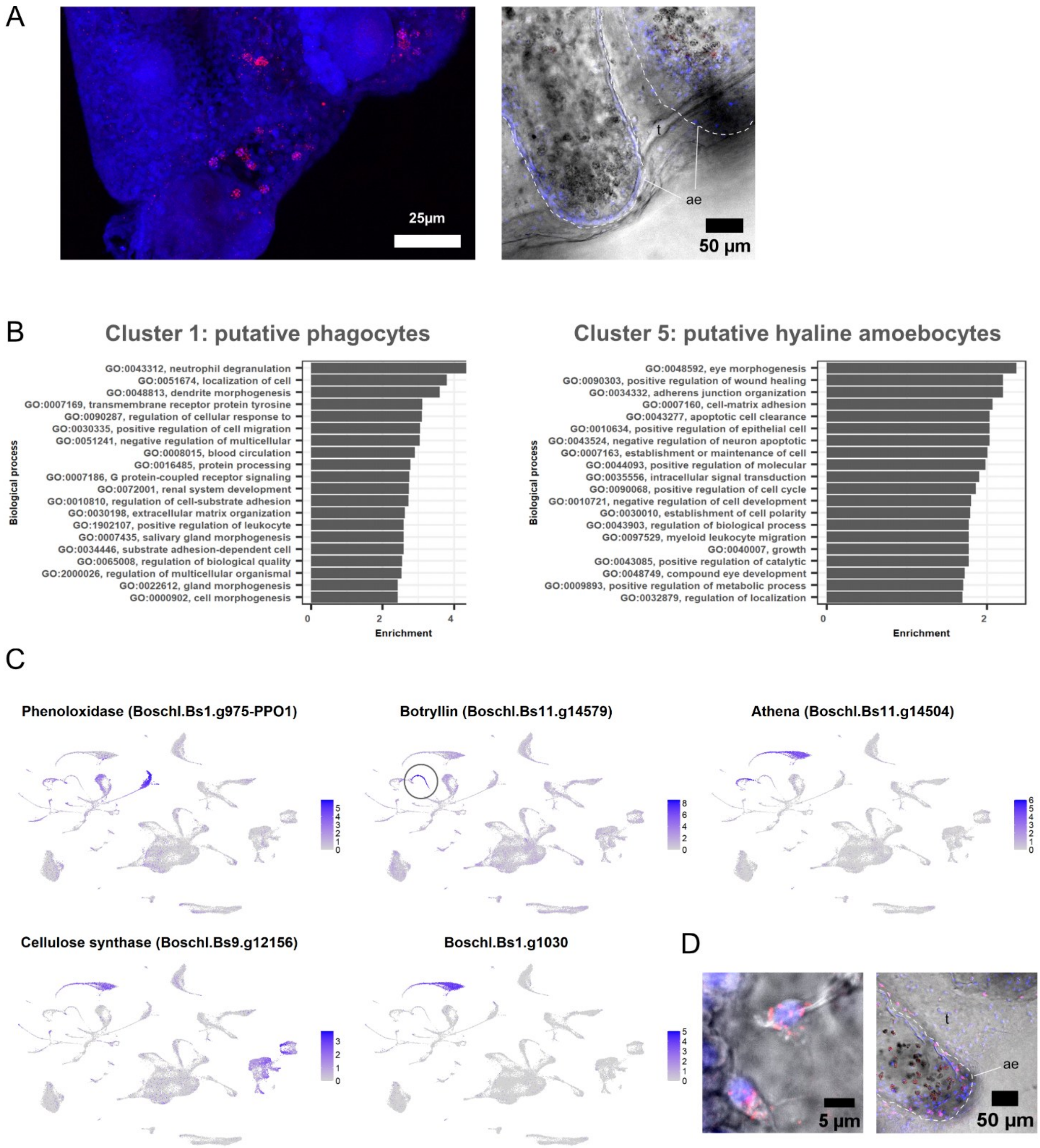

Supplementary Figure 13. Additional analysis of circulating cells clusters. (A) Negative controls of HCR showing autofluorescent morula cells in a primary bud, and no autofluorescent cells in the tunic. **(B)** 20 most significantly enriched Gene Ontology terms in various hemocyte clusters. **(C)** Expression plots of various genes known to be expressed in circulating cells. **(D)** HCR showing expression of *Boschl.Bs1.g1030* in amoeboid cells in circulation (left) and in the tunic surrounding the ampullae (right). ae: ampullae epithelium; t: tunic.

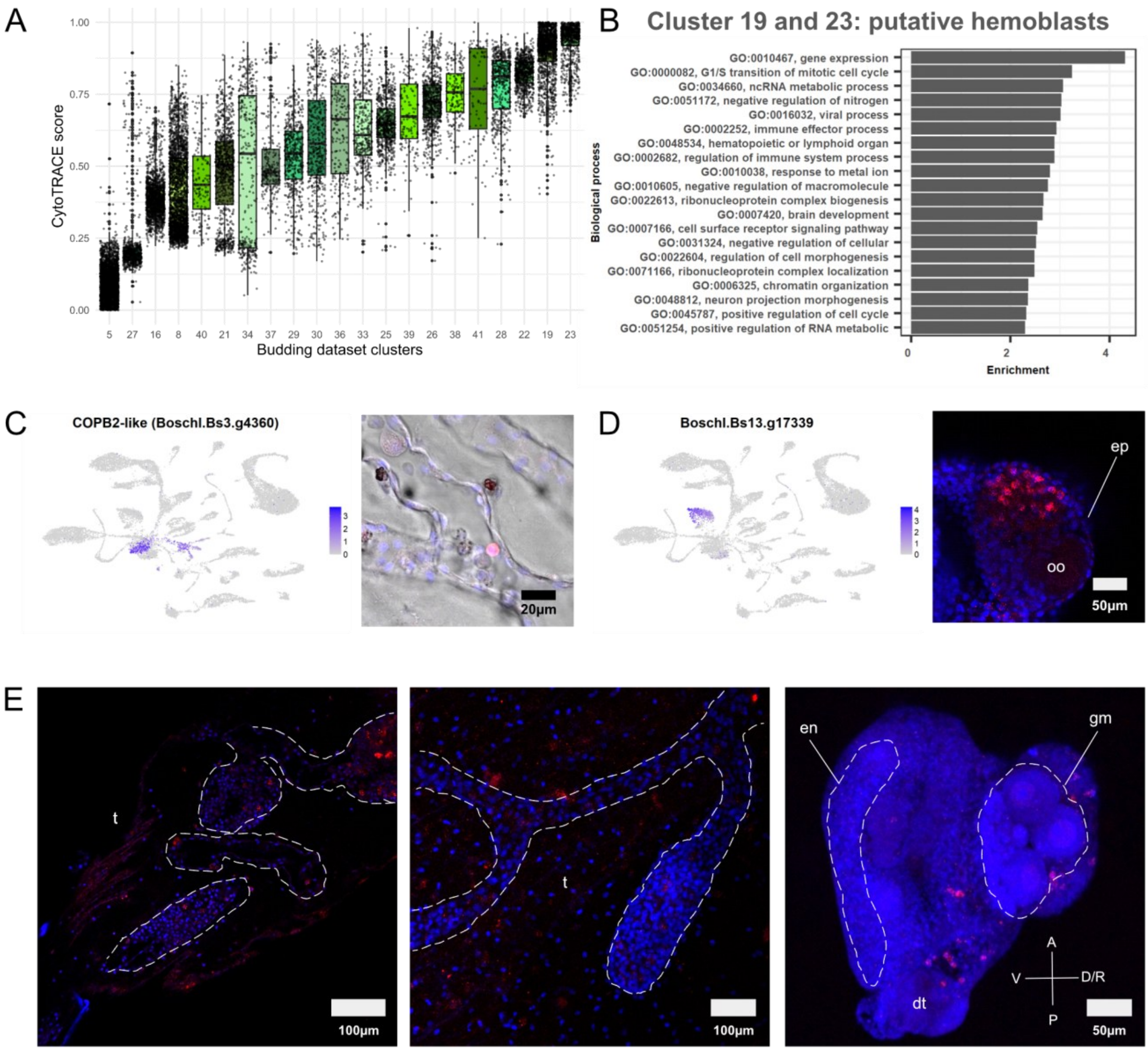

Supplementary Figure 14. Additional analysis of the putative hemoblasts. (A) CytoTRACE scores computed on the hemocyte subset, per cluster (clustering of the full budding dataset, Figure 1C; (n=16,137 cells, with 93-2924 cells per cluster), the boxplot show the median (line in the box), the first and third quartiles (the hinges of the box) and the largest/smallest value no further than 1.5 * inter-quartile range from the corresponding hinge (the whiskers), as well as any outlier beyond that point as bold points. All data are represented as small points. **(B)** 20 most significantly enriched Gene Ontology terms in the clusters 19 and 23 compared with the rest of the dataset. **(C-D)** Expression plots and HCR of marker genes of putative hemoblasts (cluster 19) and another hemocyte cluster (cluster 28) **(C)**, or resident hemoblast-like only (cluster 23) **(D)**. **(E)** Negative controls of HCR showing autofluorescent morula cells, vasculature is outlined. All presented patterns of expression have been observed in at least 3 samples of the same stage. Probe is in yellow, DAPI or Hoechst staining is in blue. ep: epidermis; oo: oocyte; t: tunic; en: endostyle; gm: gonadal mass; dt: digestive tract.

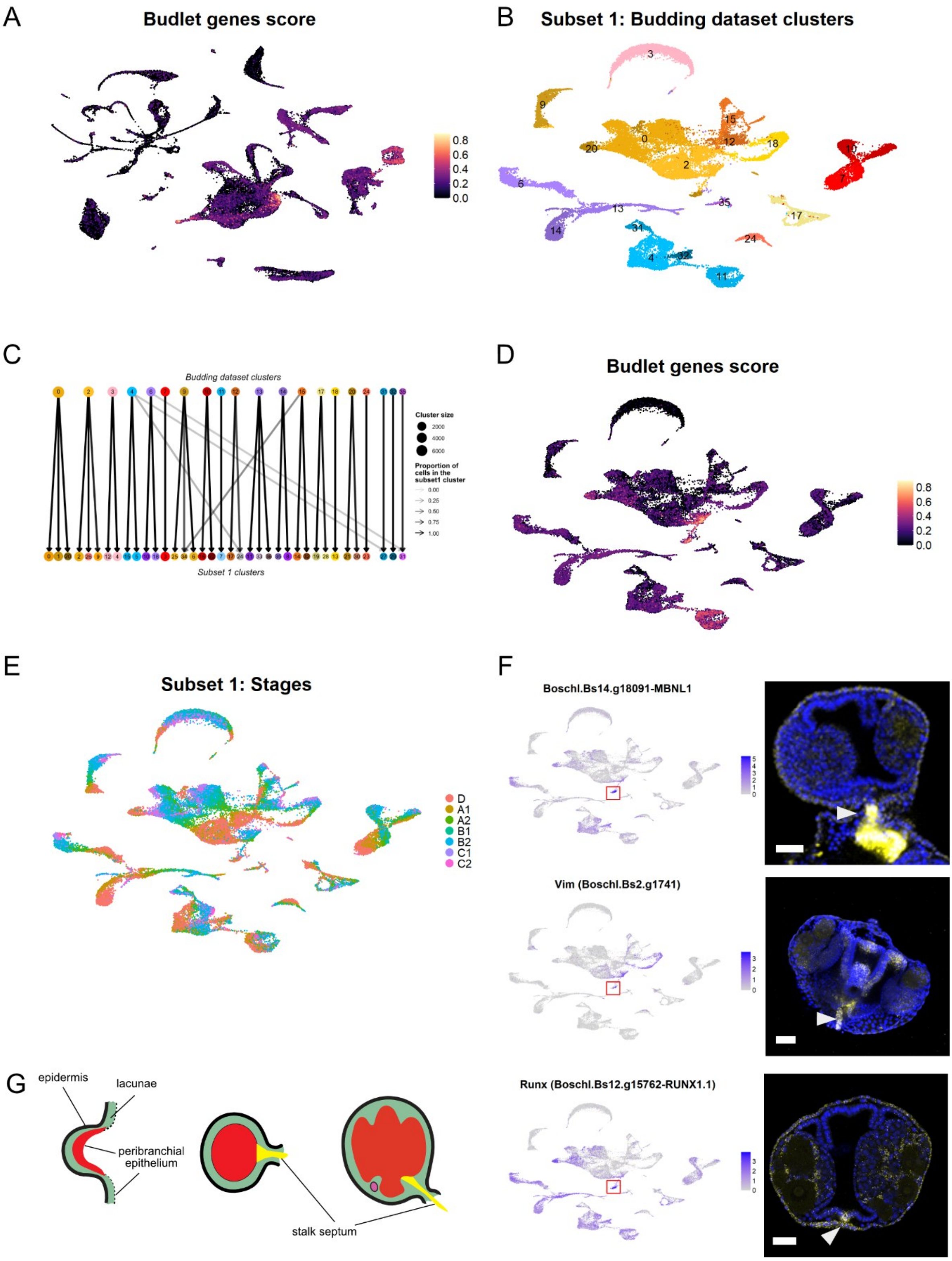

Supplementary Figure 15. Additional analyses to search for the budding founder cells. (A) UMAP of the budding dataset showing UCell gene signature score based on the expression of *Vim* (*Boschl.Bs2.g1741*), *Runx* (*Boschl.Bs12.g15762-RUNX1.1*) and *Nk4* (*Boschl.Bs6.g8341-NKX2-3*). **(B)** UMAP projection of the subset without hemocytes and putative phagocytes (subset-1), showing full budding dataset clusters. **(C)** Correspondence of cluster numbersbetween full budding dataset and

subset-1; **(D-E)** UMAP projection of the subset-1 showing UCell gene signature score based on the expression of *Vim* (*Boschl.Bs2.g1741*), *Runx* (*Boschl.Bs12.g15762-RUNX1.1*) and *Nk4* (*Boschl.Bs6.g8341-NKX2-3*) (D) and budding stages (E). **(F)** Expression plots and corresponding HCR of budlet at stage C1/C2, of genes expressed in the budlet stalk septum; red square highlights the stalk septum cluster (cluster 30). All presented patterns of expression have been observed in at least 3 samples of the same stage. Probe is in yellow, DAPI or Hoechst staining is in blue. Arrowhead: stalk septum. Scale bar 20 μm. **(G)** Schematics of the formation of the budlet stalk septum (yellow).

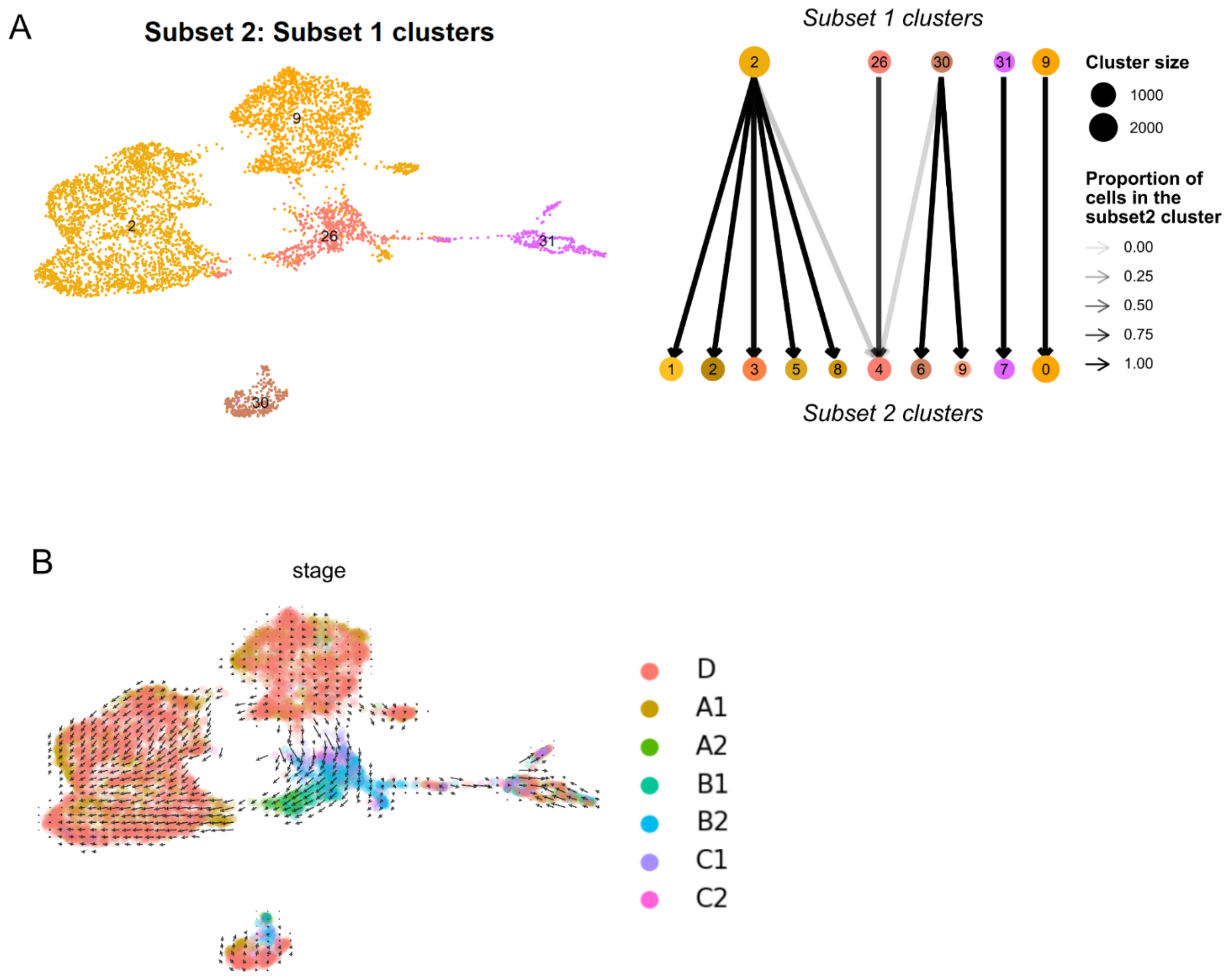

Supplementary Figure 16. Budlet founder cells are a subset of the peribranchial population. (A) UMAP projection of subset-2 containing early branchial (9) and early peribranchial (2), budlet (26), budlet stalk septum (30) and *Bhf*+ (31) cell clusters (left) and correspondence between subset-1 and subset-2 clusters (right). **(B)** Projected RNA velocity vectors for the "budlet" subset.

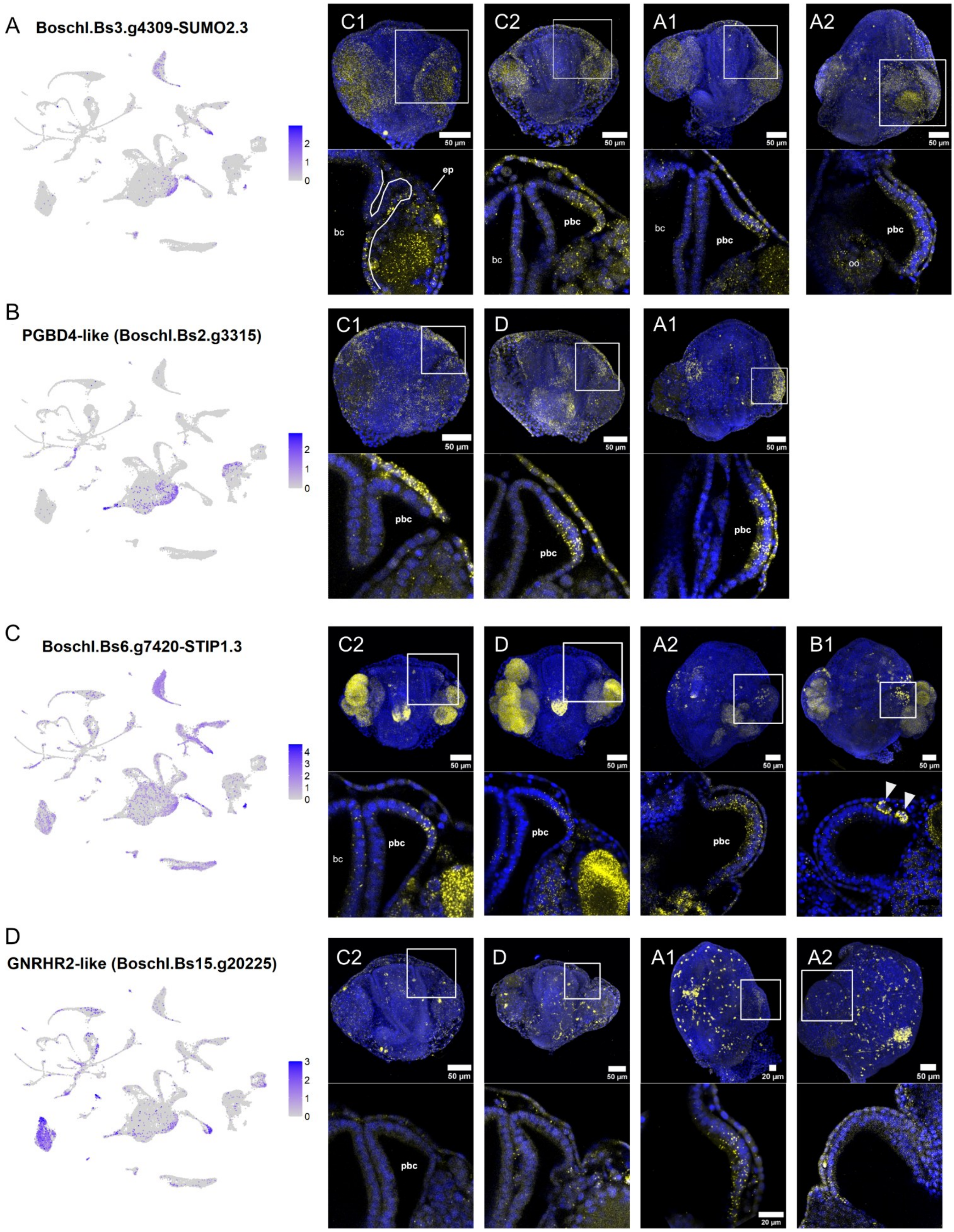

Supplementary Figure 17. Expression patterns of the budding founder cell markers over time. (A-D) Expression plots (left) and confocal images of HCR (right) on genes enriched in the budding founder cells. The top rows are maximum projections of the whole z-stack. The bottom rows

correspond to a confocal section of the budlet area squared in the top row image. Phase is on the top left. **(A)** *Sumo2-like* (*Boschl.Bs3.g4309-SUMO2.3*) is expressed from phase C1, in the external side of the peribranchial primordia, and it continues to be expressed until phase A2. The limit between the peribranchial epithelium and the gonad is highlighted by dashed curve. *Sumo2-like* is also expressed in maturing oocytes, and in some *Tgf-β-f*+ cells in the gonads **(B)** *Pgbd4-like* (*Boschl.Bs2.g3315*) first expression in the peribranchial primordia has been detected at phase D in the budlet founders. **(C)** *Stip1-like* (*Boschl.Bs6.g7420-STIP1.3*) first expression in the peribranchial primordia has been detected at stage C2 at the anterior-external side of the right peribranchial epithelium. *Stip1-like* positive mesenchymal cells near the bud (white arrowheads) probably correspond to migrating germline or to *Tgf-β-f*+ cells, consistent with its high expression in cluster 3 and 13 (see Figure 1). *Stip1-like* is also expressed in the maturing nervous system **(D)** *Gnrhr2-like* (*Boschl.Bs15.g20225*) is expressed in the budlet and the peribranchial primordia just posterior to it from the early phase A1, and can still be detected at the posterior ventral side of the budlet at phase A2 (additional budlet primordium, developing later). It is also expressed in the nervous system as well as the cells of cluster 23, with a similar expression pattern as observed from genes of this cluster (see phase D image and Figure 4K). All presented patterns of expression have been observed in at least 3 samples of the same stage. Probe is in yellow, DAPI or Hoechst staining is in blue. ep: epidermis; bc: branchial chamber; pbc: peribranchial chamber; oo: oocyte.

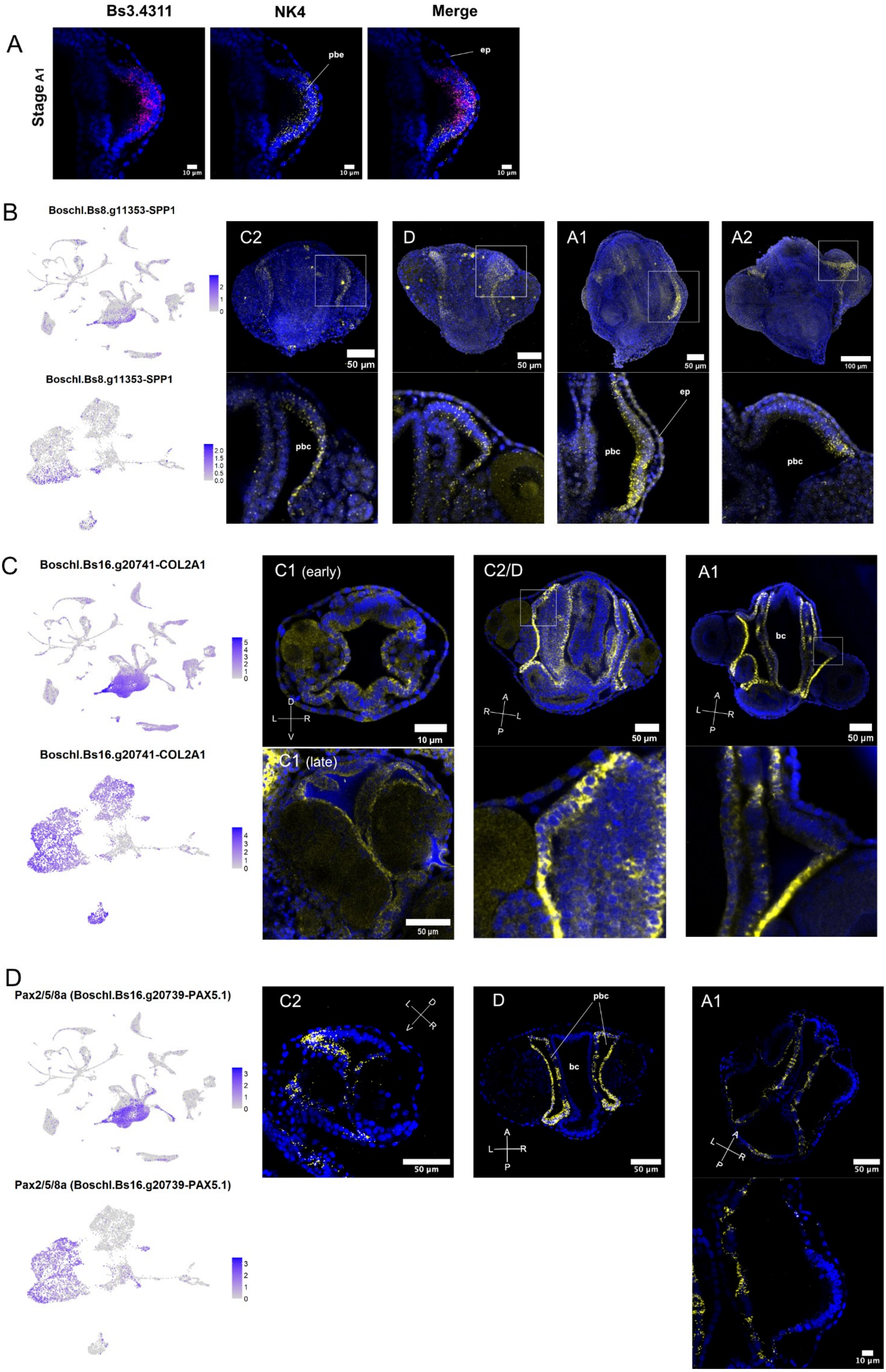

A
Bs3.4311
NK4
Merge
Stage A1
pbe
ep
10 µm
B
Boschl.Bs8.g11353-SPP1
Boschl.Bs8.g11353-SPP1
C2
D
A1
A2
50 µm
100 µm
pbc
ep
C
Boschl.Bs16.g20741-COL2A1
Boschl.Bs16.g20741-COL2A1
C1 (early)
C2/D
A1
C1 (late)
bc
10 µm
50 µm
D
Pax2/5/8a (Boschl.Bs16.g20739-PAX5.1)
Pax2/5/8a (Boschl.Bs16.g20739-PAX5.1)
C2
D
A1
pbc
bc
50 µm
10 µm

Supplementary Figure 18. Co-expression of founder cells markers and expression pattern of peribranchial epithelium genes that stop being expressed in the budlet. (A) Double HCR showing co-expression of *Boschl.Bs3.g4311* (red) and *Boschl.Bs6.g8341-NKX2-3* (yellow) in the budding founder cells of . **(B-D)** Expression plots and confocal images of **(B)** *Boschl.Bs8.g11353-SPP1* HCR and **(C)** the collagen *Boschl.Bs16.g20741-COL2A1* WMISH and **(D)** *Boschl.Bs16.g20739-PAX5.1*. All the three genes are present in the budding founder cells, but depleted in the budlet from phase A1-B2 compare to the peribranchial epithelium at later stages. The bottom rows correspond to a confocal section of the budlet area squared in the top row image. Stage is on the top left. All presented patterns of expression have been observed in at least 3 samples of the same stage. Probes are in yellow and red, DAPI or Hoechst staining is in blue. Pbe: peribranchial epithelium; ep: epidermis; bc: branchial chamber; pbc: peribranchial chamber.

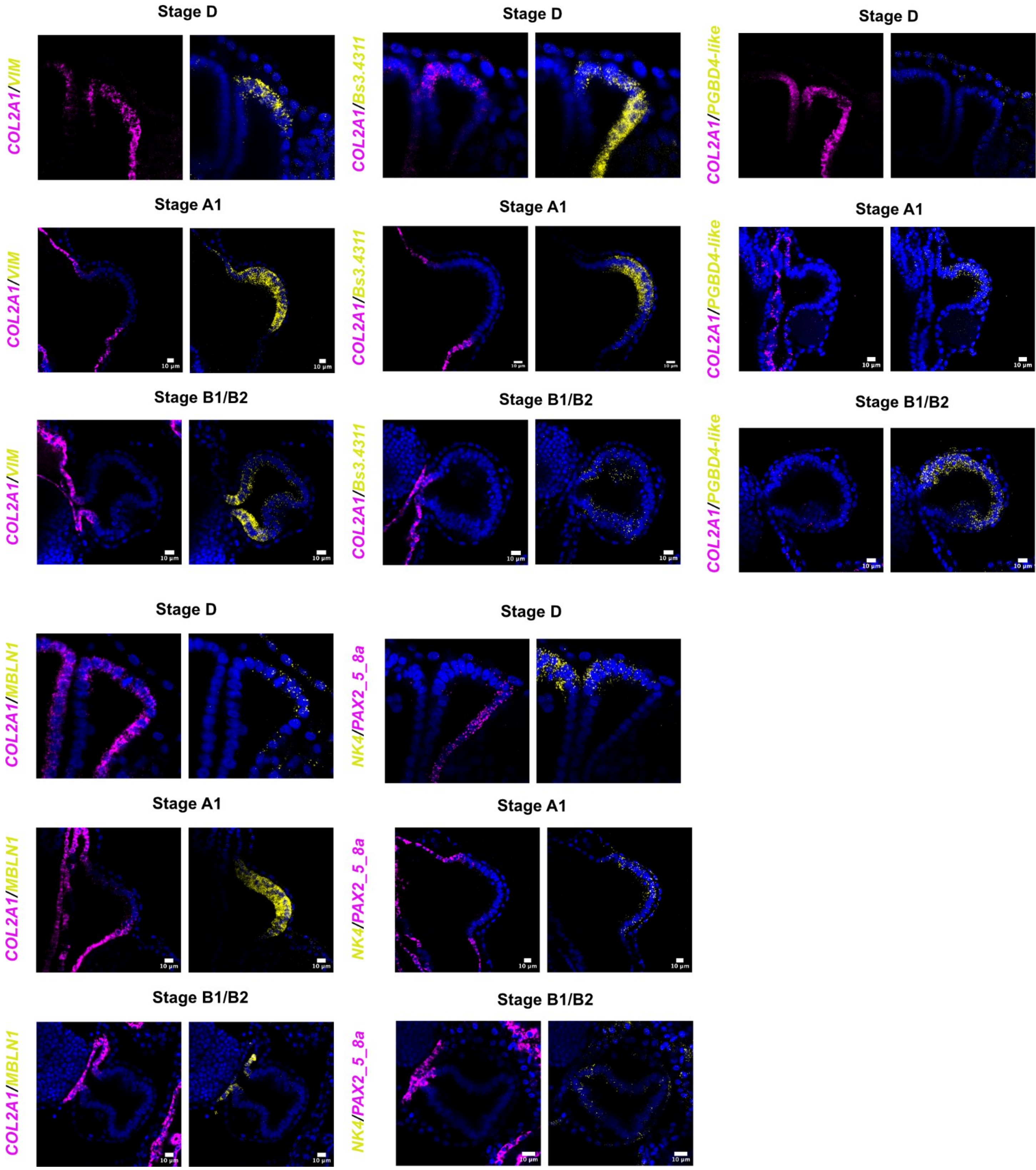
Stage D
COL2A1/VIM
Stage A1
COL2A1/VIM
Stage B1/B2
COL2A1/VIM
Stage D
COL2A1/Bs3.4311
Stage A1
COL2A1/Bs3.4311
Stage B1/B2
COL2A1/Bs3.4311
Stage D
COL2A1/PGBD4-like
Stage A1
COL2A1/PGBD4-like
Stage B1/B2
COL2A1/PGBD4-like
Stage D
COL2A1/MBLN1
Stage A1
COL2A1/MBLN1
Stage B1/B2
COL2A1/MBLN1
Stage D
NK4/PAX2_5_8a
Stage A1
NK4/PAX2_5_8a
Stage B1/B2
NK4/PAX2_5_8a

Supplementary Figure 19. Split-channel visualization of the double HCR experiments shown in Figure 7B. For each developmental stage and gene combination, the exact same confocal images presented in Figure 7B are displayed with separated fluorescence channels to facilitate visualization of the individual expression domains and their overlap. All presented patterns of expression have been observed in at least 3 samples of the same stage. Probes are in yellow and red, DAPI or Hoechst staining is in blue.
