# Supplementary material for: Multi-stage single-cell atlas of a chordate non-embryonic development unveils dedifferentiating bud founder cells
Source: Nat Commun. 2026 Sep 3;17:10486. doi: 10.1038/s41467-026-76441-3 (PMC13635237; doi:10.1038/s41467-026-76441-3)
Supplement: Supplementary file 2 [file 41467_2026_76441_MOESM2_ESM.pdf]

Description of Additional Supplementary Files

File Name: Supplementary Data 1

Description: List of *in situ* hybridization probes.
