# Supplementary material for: Multi-stage single-cell atlas of a chordate non-embryonic development unveils dedifferentiating bud founder cells
Source: Nat Commun. 2026 Sep 3;17:10486. doi: 10.1038/s41467-026-76441-3 (PMC13635237; doi:10.1038/s41467-026-76441-3)
Supplement: Supplementary file 3 [file 41467_2026_76441_MOESM4_ESM.pdf]

nature portfolio

Corresponding author(s): *Double-anonymous peer review submissions: write DAPR and your manuscript number here instead of author names.*

Last updated by author(s): *YYYY-MM-DD*

Reporting Summary

Nature Portfolio wishes to improve the reproducibility of the work that we publish. This form provides structure for consistency and transparency in reporting. For further information on Nature Portfolio policies, see our Editorial Policies and the Editorial Policy Checklist.

Statistics

For all statistical analyses, confirm that the following items are present in the figure legend, table legend, main text, or Methods section.

n/a	Confirmed	
☐	☒	The exact sample size (*n*) for each experimental group/condition, given as a discrete number and unit of measurement
☐	☒	A statement on whether measurements were taken from distinct samples or whether the same sample was measured repeatedly
☐	☒	The statistical test(s) used AND whether they are one- or two-sided<br>*Only common tests should be described solely by name; describe more complex techniques in the Methods section.*
☒	☐	A description of all covariates tested
☐	☒	A description of any assumptions or corrections, such as tests of normality and adjustment for multiple comparisons
☐	☒	A full description of the statistical parameters including central tendency (e.g. means) or other basic estimates (e.g. regression coefficient) AND variation (e.g. standard deviation) or associated estimates of uncertainty (e.g. confidence intervals)
☒	☐	For null hypothesis testing, the test statistic (e.g. *F*, *t*, *r*) with confidence intervals, effect sizes, degrees of freedom and *P* value noted<br>*Give P values as exact values whenever suitable.*
☒	☐	For Bayesian analysis, information on the choice of priors and Markov chain Monte Carlo settings
☒	☐	For hierarchical and complex designs, identification of the appropriate level for tests and full reporting of outcomes
☒	☐	Estimates of effect sizes (e.g. Cohen's *d*, Pearson's *r*), indicating how they were calculated

Our web collection on statistics for biologists contains articles on many of the points above.

Software and code

Policy information about availability of computer code

Data collection	No software was used for data collection.<br>The publicly available mitochondrial genome was downloaded from GenBank (HF548551.1)
Data analysis	Scripts and markdown used to analyse the data will be made available on github upon publication<br>Libraries: CellRanger count (v7.1.0 for budding datasets, v6.1.2 for circulating cells datasets), Seurat (v5.1.0), Bioconductor (v3.18) , scanPy (v1.9.8), scDblFinder (v1.16.0), Clustree (v0.5.1), Eggnog emapper (v2.1.10), topGO (v2.54.0), CytoTRACE (v0.3.3), PAGA, Velocyto (v0.17), scVelo (v0.3.2), sceasy (v0.0.7), Monocle3 (v1.3.7)

For manuscripts utilizing custom algorithms or software that are central to the research but not yet described in published literature, software must be made available to editors and reviewers. We strongly encourage code deposition in a community repository (e.g. GitHub). See the Nature Portfolio guidelines for submitting code & software for further information.

Data

Policy information about availability of data

All manuscripts must include a data availability statement. This statement should provide the following information, where applicable:

- Accession codes, unique identifiers, or web links for publicly available datasets
- A description of any restrictions on data availability
- For clinical datasets or third party data, please ensure that the statement adheres to our policy

Sequencing data will be made available on public repositories upon publication

Research involving human participants, their data, or biological material

Policy information about studies with human participants or human data. See also policy information about sex, gender (identity/presentation), and sexual orientation and race, ethnicity and racism.

Reporting on sex and gender	This study did not involve research in humans.
Reporting on race, ethnicity, or other socially relevant groupings	n/a
Population characteristics	n/a
Recruitment	n/a
Ethics oversight	n/a

Note that full information on the approval of the study protocol must also be provided in the manuscript.

Field-specific reporting

Please select the one below that is the best fit for your research. If you are not sure, read the appropriate sections before making your selection.

☒ Life sciences ☐ Behavioural & social sciences ☐ Ecological, evolutionary & environmental sciences

For a reference copy of the document with all sections, see nature.com/documents/nr-reporting-summary-flat.pdf

Life sciences study design

All studies must disclose on these points even when the disclosure is negative.

Sample size	Sample size for the scRNA-seq dataset were determined by the technical limitations of the 10X Chromium 3' v3 kits, recommendning around 10,000 cells per sample to reduce the probaility of encapsulating doublets. Sample sizes for ISH and HCR was determined to ensure qualitative reproductibility of the patterns of expression.
Data exclusions	Lower quality cells were excluded from the analysis. The exact parameters and reasoning for the exclusion are detailed in the manuscript.
Replication	Only one stage (B2) had a biological replicate.
Randomization	This study did not involved randomization as no specific effect was compared. Buds were collected at random in colonies of the right stage.
Blinding	Blinding is not relevant for this study.

Reporting for specific materials, systems and methods

We require information from authors about some types of materials, experimental systems and methods used in many studies. Here, indicate whether each material, system or method listed is relevant to your study. If you are not sure if a list item applies to your research, read the appropriate section before selecting a response.

Materials & experimental systems

n/a	Involved in the study	
☐	☒	Antibodies
☒	☐	Eukaryotic cell lines
☒	☐	Palaeontology and archaeology
☐	☒	Animals and other organisms
☒	☐	Clinical data
☒	☐	Dual use research of concern
☒	☐	Plants

Methods

n/a	Involved in the study	
☒	☐	ChIP-seq
☒	☐	Flow cytometry
☒	☐	MRI-based neuroimaging

Antibodies

Antibodies used	Anti-phospho-histone 3 (Abcam #AB176840)
Validation	*Describe the validation of each primary antibody for the species and application, noting any validation statements on the manufacturer's website, relevant citations, antibody profiles in online databases, or data provided in the manuscript.*

Animals and other research organisms

Policy information about studies involving animals; ARRIVE guidelines recommended for reporting animal research, and Sex and Gender in Research

Laboratory animals	*For laboratory animals, report species, strain and age OR state that the study did not involve laboratory animals.*
Wild animals	For the budding samples, young oozoids were collected on glass slides from the port of Villefranche-sur-Mer (43.697882, 7.30735), then isolated in small containers (~1 L) at 18°C to let them grow for a few weeks. Once the colonies had 10-20 blastozooids, they were transferred to a cold marine culture system (12°C) with semi-open circulation of 5µm-filtered natural sea water. One of these isogenic colonies (labelled as FC*) was chosen to perform all the dissociations.<br><br>For the circulating cell samples, animals were collected in Santa Barbara, California, USA. Colonies were attached to glass slides with sewing thread and allowed to grow onto slides. Colonial animals were allowed to grow for a couple of months in the lab. They were then shipped overnight to Boston.
Reporting on sex	Botryllus schlosseri are hermaphroditic animals, with both male and female gametes. The individuals used in this study werer kept at low temperatures inhibiting full sexual maturation
Field-collected samples	For the budding samples, animals were were fed with a mix of live Tisochrysis lutea, live Chaetoceros gracilis, ISO800 and Shellfish Diet 1800 (Reed Mariculture) and kept in a cold marine culture system (12°C) described previously (Langenbacher et al. 2015).<br><br>For the circulating cell samples, animals were first kept in Santa Barbara in a semi flow through mariculture system with 0.5µm-filtered seawater at 16-18 ºC, and they were fed twice a day with cultures of the following live microalgae: Dunaliella sp. (Carolina Biological CAT#152160), Isochrysis sp. (Carolina Biological CAT#153180), Nannochloropsis sp. (Carolina Biological CAT#153220), Tetraselmis sp. (Carolina Biological CAT#152610). In Boston, they were kept in artificial sea water (ASW, Instant Ocean SS15-10) at 17 ºC.
Ethics oversight	No ethical approval or guidance was needed. Ascidians are invertebrates that fall outside the EU Directive 2010/63/EU of the European Parliament and of the Council of 22 September 2010 on the protection of animals for scientific purposes.

Note that full information on the approval of the study protocol must also be provided in the manuscript.

Plants

Seed stocks	*Report on the source of all seed stocks or other plant material used. If applicable, state the seed stock centre and catalogue number. If plant specimens were collected from the field, describe the collection location, date and sampling procedures.*
Novel plant genotypes	*Describe the methods by which all novel plant genotypes were produced. This includes those generated by transgenic approaches, gene editing, chemical/radiation-based mutagenesis and hybridization. For transgenic lines, describe the transformation method, the number of independent lines analyzed and the generation upon which experiments were performed. For gene-edited lines, describe the editor used, the endogenous sequence targeted for editing, the targeting guide RNA sequence (if applicable) and how the editor was applied.*
Authentication	*Describe any authentication procedures for each seed stock used or novel genotype generated. Describe any experiments used to assess the effect of a mutation and, where applicable, how potential secondary effects (e.g. second site T-DNA insertions, mosiacism, off-target gene editing) were examined.*
