# Supplementary material for: Multi-stage single-cell atlas of a chordate non-embryonic development unveils dedifferentiating bud founder cells
Source: Nat Commun. 2026 Sep 3;17:10486. doi: 10.1038/s41467-026-76441-3 (PMC13635237; doi:10.1038/s41467-026-76441-3)
Supplement: Supplementary file 4 [file 41467_2026_76441_MOESM5_ESM.pdf]

Peer Review File

Multi-stage single-cell atlas of a chordate non-embryonic development unveils dedifferentiating bud founder cells

Corresponding Author: Dr Stefano Tiozzo

This file contains all reviewer reports in order by version, followed by all author rebuttals in order by version.

Version 0:

Reviewer comments:

Reviewer #1

(Remarks to the Author)
The manuscript by Lebel et al. presents a novel single-cell transcriptomic atlas for the colonial ascidian Botryllus schlosseri, by collecting buds and budlets at various colonial developmental phases. It represents a new wool of research offered to the scientific community. Using this atlas, the authors propose some interesting differentiattion trajectories of cells, with particular reference to haemocyte differentiation. The same atlas, led the authors to identify, in the peribranchial epithelium, the putative founder cells of the new bud generation supporting the hypothesis that buds derive from de-differentiation and re-differentiation of cells of the peribranchial epithelium.
In my opinion, the manuscript is of great importance to scholars involved in chordate research and the study of asexual reproduction. I warmly support its publication after a minor revision. I have only few comments listed below:

1. Page 3, line 56. I suggest to use the term "phase" instead of "stage" as what called "A", "B", "C" and "D" include the developmental stages of adult zooids, buds and budlets.
2. Page 7, fig. 1A. The figure was modified according to Ballarin et al., 2008 (Cell Tisse Res), not fron Lauzon et al., 2002).
3. In their 2009 paper, Rosner et al., using an immunocytochemical approach, claimed that piwi and vasa are expressed also by cells other than cells of the germ line, such as phagocytes and epithelial cells. This seems to be in contrast with the sentence in the text saying that "...the role of piwi and vasa appears to be confined to the germline. Did the authors consider this point?

Reviewer #2

(Remarks to the Author)
In this paper, Lebel et al use single cell RNAseq to follow the changes of gene expression on the cellular level during budding of the colonial urochordate Botryllus. Previous work has postulated the role of migratory multipotent stem cells in this process. Lebel et al first generate a developmental cell atlas covering multiple stages of zooid formation and carefully validate most cell clusters by HCR. They use several methods of trajectory analyses to reconstruct the differentiation program during zooid formation. They conclude that new cell types of zooids derive through dedifferentiation rather than from circulating stem cells.

To my knowledge this represents the first large single cell atlas of this colonial urochordate model organism. This will provide a very important resource for the community and a basis for comparison with other urochordates and chordates in general. The bioinformatic analyses and the experimental validations are careful and thorough. The paper is well written and the data of high quality. It therefore certainly deserves publication in Nature Communications. However, while the authors are unable to identify the postulated stem cell population, the support for dedifferentiation from a certain part of the epithelial cells necessarily is based on the absence of evidence for multipotent stem cells. They argue that the previously identified circulating cells are stem cells of the blood system and of the gametes, but not of other somatic cell types. This is supported by the independent sequencing of cells “bleeding” from the vasculature. These cell populations show profiles of hemocytes and gamete precursors, as well as some possible phagocytic cells. They identify part of the peribranchial epithelium to undergo the dedifferentiation. This is based on the downregulation of differentiation markers and the upregulation of several specific genes marking the dedifferentiated cell status e.g. This is convincingly demonstrated by the localized gene expression through HCR, marking the future region of zooid budding. However, even though I think the conclusion is quite plausible, there is no direct experimental proof, e.g. genetic labeling by Cre-Lox, transplantation or gene knockdowns of the

stem cell markers. It is unclear to me, whether this is even technically possible in Botryllus. I therefore recommend to tone down the conclusions and keep the door open for other explanations.
More specific comments are below:

1) While I find the conclusion that there might not be a dedicated multipotent migratory stem cell population required for bud formation fairly plausible, I still wonder whether they can exclude that the circulatory TGFb-f+ cells or the putative phagocytic cells might act as such stem cells. If not, they should still raise and discuss this possibility in the light of previous publications by Rinkevich et al.

2) On page 17, the authors state that the four circulating cell populations have only minimal connection suggesting that they may arise from separate lineages. However, it is well possible that the intermediate stages have not been captured (as conceded by the authors as well). This is indeed the case in many other scRNAseq dataset where the lineages are well established, yet the connection appears at times disrupted, if the intermediate stages are very short in comparison to the early and late stages. Should be toned down, unless there is experimental evidence.

Minor comments:
Line 150: clusters 0,2, 20 are annotated as branchial and peribranchial epithelia, but they seem to overlap with TFs characteristic for the heart. What is the connection between these tissues?

Uli Technau

Reviewer #3

(Remarks to the Author)
This manuscript uses single-cell sequencing approaches to probe the cellular and gene regulatory basis of bud emergence in the tunicate Botryllus schlosseri. As one of the intriguing features of the system, individuals undergo asexual reproduction via budding of new individuals (zooids) from adult animals. This process raises a number of interesting questions in terms of cell and evolutionary biology. As nicely explained in the introduction, a long-standing question in the field concerns the origin of the bud founder cells, with circulating pluripotent stem cells as one option and somatic epithelial cells as another. Building largely on the analysis of two extensive single-cell sequencing data sets, the authors provide further evidence for the epithelial origin of the zooid.

Overall, the manuscript is well written and the analyses are generally sound (see technical questions below). The first part of the manuscript is essentially a cell-atlas-style description of cell types in adults and emerging zooids in B. schlosseri, combining scRNA-seq data with a seemingly new single-channel HCR-based protocol to localize the corresponding cell types within the animal. The analysis of the bud founder cells in Figures 6 and 7 combines lineage analyses based on the single-cell data sets with HCR localizations. By the nature of the work, large sections of the manuscript are model-organism specific, but they could be made more accessible to non-tunicate researchers (see comments below). However, the origin of the zooid founder cells is of broad interest to the cell and developmental biology community. With the addition of orthogonal evidence further corroborating the sequencing data, I can recommend publication of this manuscript in Nature Communications.

Major points

1) For non-specialists, the expression data are often hard to interpret. This is because many of the sections are not annotated and appear to be cut and oriented in different planes (Fig. 2, Fig. 3c). A general anatomy explainer cartoon and clear indication of the tissues represented in specific sections would greatly improve the accessibility of these data.

2) The lineage reconstructions are certainly interesting and, in combination with the de-differentiation data, are supportive of an epithelial origin of the zooid founder cells. However, given the caveats of scRNA-seq data, especially for detecting rare events, orthogonal lines of evidence on this important point would be very valuable. If morpholino or RNAi approaches remain difficult in this system, whole-mount in situ hybridizations with the new markers might help to delineate the earliest events in zooid formation. Alternatively, could maybe lineage tracing based on SNPs in the scRNA-seq data help corroborate the somatic/epithelial origin of the zooids?

3) The cell counts reported in Supplementary Table 2 suggest that sample ML245-D exceeded 20,000 cells, which is curious given the technical limitations of the 10x Chromium kit. Can you please explain the origin of the high cell count in this case? Similarly, the need to remove apparent low-quality clusters during cluster subsetting is somewhat unusual and may point to issues with the initial cell-filtering cutoffs. Please comment and include barcode-rank plots of the data in Supplementary Table 2 to allow a better appreciation of the data quality across samples.

Minor points

In Supplementary Figure 1B, all three flow cytometry panels are labelled as ML208, whereas the legend states that the figure corresponds to ML231-B2. Please clarify this inconsistency.

In line 227, planarians should be added to the list of animals with adult pluripotent stem cells, as they were instrumental in defining this cell class.

Version 1:

Reviewer comments:

Reviewer #1

(Remarks to the Author)
I confirm my approval of the publication of the manuscript by Lebel et al. However, I regret to say that my previous suggestions for improvement were almost completely neglected.

As I said in my previous revision, Fig. 1A is modified from Ballarin et al.,2008, but this was not modified in the text (now it appears from Wolf et al., 2019), neither was the citation present among the references

Additionally, and more important, for sake of clarity, I suggested to replace the term “stage” with “phase” in most of the cases where the term appears. This suggestion was not taken into consideration but it is useful in order to avoid confusion. If it is correct to deal with the developmental stages of the zooid, from its appearance ad thickening of the peribranchial epithelium to the resorption at the takeover (11 stages according to Berrill (1941) and Sabbadin (1955); 12 stages according to Izzard (1973)), it is not correct to use the same term to indicate the cyclical changes in a colony. They should be called “phases” and each phase includes the developmental stages of zooids, buds and budlets. They are identified with four letters (A-D) by the Japanese and American researchers (and in this manuscript) and with a triplet of numbers referring to the developmental stages of the three colonial generations by the Italian scientists. As a consequence, the expressions “budlet stage D” or “bud stage A1” (page 12, line 6) should be replaced with “budlet (from colony) at phase D” or “bud (from colony) at phase A1”.
I summarise here below the parts of the manuscript deserving changes:
Page 3, line 27. Here, the term “stage” is correct and should be maintained
Page 3, line 28. “Phase D” instead of “stage D”
Page 6, lines 4, 5,20. Replace “stage” with “phase” four times
Figure 2. “phase A1-2”, “phase B1-2”, “phase “C1-2”
Page 12, lines 6-7.
Page 17, line 27
Page 18, line 21
Page 21, lines 14. “A1-C2 budlet cells” should be modified to “budlet cells from phase A1-C2”
Page 21, lines 27. “A2-C2 stage budlet cells” should be modified to “budlet cells from phase A2-C2”
Page 23, lines 5, 16
Page 24, line 1
Page 26, lines 12, 16, 17, 18
Page 28, line 23
Page 29, line 11
Page 31, lines 27, 28
Page 32, lines 12, 13
Page 33, line 6
Page 34, line 9. Replace “A1 sample” and “B2 sample” with “sample from phase A1” and “sample from phase B2”
Suppl. Table 1. “Phase” instead of “stage” in the title of the2nd column
Suppl. Fig. 2A replace “A1 stage circulating cells” and “B2 stage circulating cells” with “circulating cells from phase A1 “ and “circulating cells form phase B2”
Suppl. Fig. 5A. “Budding dataset clusters, shown by phase”
Suppl. Fig. 5B. “replace “B2 stage samples” with “samples from phase B2”. In the title of the legend replece “stage” with “phase”. In the legend of the part B replace “B2 samples” with “samples from phase B2”
Page 75 (legend of suppl. Fig. 17), lines 2, 3, 6, 9, 15, 17
Page 77 (legend of suppl. Fig. 18), line 7. “A1-B2 budlet” should be replaced with “budlet from phase A1-B2”

Reviewer #2

(Remarks to the Author)
The authors carefully revised the manuscript and fully satisfied my and the other reviewers comments in my view. They prudently toned down the conclusions leaving room for alternative explanations and the limitations of the study. Taken together, this is a beautiful, and important piece of work. I recommend to accept it now.

One last little thing:
Pg. 9. "are also involved pharyngeal development"
should be: are also involved in pharyngeal development

Reviewer #3

(Remarks to the Author)
Thank you- the revisions conclusively address all my concerns. I have no further concerns and congratulate the authors on a

very nice piece of work.

Open Access This Peer Review File is licensed under a Creative Commons Attribution 4.0 International License, which permits use, sharing, adaptation, distribution and reproduction in any medium or format, as long as you give appropriate credit to the original author(s) and the source, provide a link to the Creative Commons license, and indicate if changes were made.
In cases where reviewers are anonymous, credit should be given to 'Anonymous Referee' and the source.
The images or other third party material in this Peer Review File are included in the article's Creative Commons license, unless indicated otherwise in a credit line to the material. If material is not included in the article's Creative Commons license and your intended use is not permitted by statutory regulation or exceeds the permitted use, you will need to obtain permission directly from the copyright holder.
To view a copy of this license, visit https://creativecommons.org/licenses/by/4.0/

Response to Reviewers
Manuscript: "*Multi-stage single-cell atlas of a chordate non-embryonic development unveils dedifferentiating bud founder cells*"
(NCOMMS-25-87463)

We sincerely appreciate the reviewers' positive and constructive feedback, which have helped us significantly improve the manuscript. Below, we reproduce each comment in full, point by point (in *italics*), followed by our response.

We also provide a tracked-changes version of the revised manuscript, in which all revisions can be readily identified. Please note that while the pages and line numbers quoted by the Reviewers refer to the submitted version of the manuscript prior peer review, the coordinates in the responses (page and line numbers) refers to the revised article with the changes highlighted.

Reviewer #1

(Remarks to the Author):The manuscript by Lebel et al. presents a novel single-cell transcriptomic atlas for the colonial ascidian Botryllus schlosseri*, by collecting buds and budlets at various colonial developmental phases. It represents a new wool of research offered to the scientific community. Using this atlas, the authors propose some interesting differentiattion trajectories of cells, with particular reference to haemocyte differentiation. The same atlas, led the authors to identify, in the peribranchial epithelium, the putative founder cells of the new bud generation supporting the hypothesis that buds derive from de-differentiation and re-differentiation of cells of the peribranchial epithelium.In my opinion, the manuscript is of great importance to scholars involved in chordate research and the study of asexual reproduction. I warmly support its publication after a minor revision. I have only few comments listed below:*

Response: We thank the reviewer for her/his constructive reading and for emphasizing the value for the community

1. Page 3, line 56. I suggest to use the term "phase" instead of "stage" as what called "A", "B", "C" and "D" include the developmental stages of adult zooids, buds and budlets.

Response: done (Page 3, line 27)

2. Page 7, fig. 1A. The figure was modified according to Ballarin et al., 2008 (Cell Tisse Res), not fron Lauzon et al., 2002).

Response: We have added the reference Ballarin et al. (2008) to the figure. The reference Lauzon et al. (2002) is cited specifically in relation to the staging method and has therefore been retained (Page 7).

3. In their 2009 paper, Rosner et al., using an immunocytochemical approach, claimed that piwi and vasa are expressed also by cells other than cells of the germ line, such as phagocytes and epithelial cells. This seems to be in contrast with the sentence in the text saying that "...the role of piwi and vasa appears to be confined to the germline. Did the authors consider this point?

Response: With the protocols available in Rosner et al. (2009), it is difficult to clearly distinguish background noise from true signal, making the assessment of Piwi and Vasa expression in epithelia and phagocytic cells uncertain. We nevertheless acknowledged their observations and revised the sentence

(Page 14 lines 12-15) in " […] *Piwi* and *Vasa* appears to the be largely restricted to the germline[25,52]. Only limited protein expression has been reported outside this context, notably in the digestive epithelium and in putative phagocytic cells only in the adult zooid[53]".

We did add the reference Rosner et al. (2009).

Reviewer #2

(Remarks to the Author): *In this paper, Lebel et al use single cell RNAseq to follow the changes of gene expression on the cellular level during budding of the colonial urochordate Botryllus. Previous work has postulated the role of migratory multipotent stem cells in this process. Lebel et al first generate a developmental cell atlas covering multiple stages of zooid formation and carefully validate most cell clusters by HCR. They use several methods of trajectory analyses to reconstruct the differentiation program during zooid formation. They conclude that new cell types of zooids derive through dedifferentiation rather than from circulating stem cells.*

To my knowledge this represents the first large single cell atlas of this colonial urochordate model organism. This will provide a very important resource for the community and a basis for comparison with other urochordates and chordates in general. The bioinformatic analyses and the experimental validations are careful and thorough. The paper is well written and the data of high quality. It therefore certainly deserves publication in Nature Communications. However, while the authors are unable to identify the postulated stem cell population, the support for dedifferentiation from a certain part of the epithelial cells necessarily is based on the absence of evidence for multipotent stem cells. They argue that the previously identified circulating cells are stem cells of the blood system and of the gametes, but not of other somatic cell types. This is supported by the independent sequencing of cells "bleeding" from the vasculature. These cell populations show profiles of hemocytes and gamete precursors, as well as some possible phagocytic cells. They identify part of the peribranchial epithelium to undergo the dedifferentiation. This is based on the downregulation of differentiation markers and the upregulation of several specific genes marking the dedifferentiated cell status e.g. This is convincingly demonstrated by the localized gene expression through HCR, marking the future region of zooid budding. However, even though I think the conclusion is quite plausible, there is no direct experimental proof, e.g. genetic labeling by Cre-Lox, transplantation or gene knockdowns of the stem cell markers. It is unclear to me, whether this is even technically possible in Botryllus. I therefore recommend to tone down the conclusions and keep the door open for other explanations.

Response: We thank Reviewer #2 for the very positive evaluation of our work and for recognizing both the quality of the dataset and its value as a resource for the community. We fully agree that, in the absence of direct functional lineage-tracing or genetic manipulation approaches in Botryllus, our conclusions should be framed more cautiously. Accordingly, we have moderated several statements in the manuscript and revised the Discussion to more explicitly acknowledge the limitations of the current experimental toolbox, while keeping open alternative interpretations consistent with the data (see details below).

More specifically we modified: Page 26, lines 7, 26-30 ; Page 27, lines 18-21, 23-25, 28-30; Page 28, lines 3, 4, 6-7, 9-13, 15-19, 25-31; page 29 lines 1-4; Page 30, lines 3, 8-13.

More specific comments are below:

1) While I find the conclusion that there might not be a dedicated multipotent migratory stem cell population required for bud formation fairly plausible, I still wonder whether they can exclude that the circulatory TGFb-f+ cells or the putative phagocytic cells might act as such stem cells. If not, they should still raise and discuss this possibility in the light of previous publications by Rinkevich et al.

Response: We thank the reviewer for raising this important point. We agree that, based on our data, we cannot formally exclude the possibility that specific circulating populations—particularly Tgf-β-f^{+} cells—may contribute to budding under certain contexts.

In our dataset, Tgf-β-f^{+} cells form a transcriptionally heterogeneous group, largely associated with germline support and gonadal compartments, and only rarely detected in circulation (Fig. 5A–B). Importantly, we do not observe a clear transcriptional or trajectory-based continuity between these populations and the identified peribranchial founder cells initiating budlet formation (Fig. 6C–F). Similarly, the putative phagocytic population, while occasionally present near budding tissues and enriched for immune and migratory signatures, does not show molecular features indicative of epithelial integration or lineage continuity with bud founders.

That said, our single-cell transcriptomic approach cannot definitively rule out rare, transient, or context-dependent contributions of circulating cells, as previously proposed based on grafting and lineage-competition experiments (e.g. Rinkevich et al. 2013 *Dev. Cell*, Voskoboynik et al. 2008 *C ell Stem Cell*).

We have therefore revised the Discussion to explicitly acknowledge this limitation and to frame our conclusions as excluding a *mandatory* or *dominant* role for a dedicated migratory multipotent stem cell population in peribranchial budding, rather than excluding any contribution under all conditions (Page 28, lines 3-7, 9-13, 14-16).

2) On page 17, the authors state that the four circulating cell populations have only minimal connection suggesting that they may arise from separate lineages. However, it is well possible that the intermediate stages have not been captured (as conceded by the authors as well). This is indeed the case in many other scRNAseq dataset where the lineages are well established, yet the connection appears at times disrupted, if the intermediate stages are very short in comparison to the early and late stages. Should be toned down, unless there is experimental evidence.

Response: We agree with the Reviewer comment and we modified the text accordingly: Page 17, lines 22-23; Page 27 lines 28-30; Page 28 lines 3-7; Page 30, lines 8-13.

Minor comments:

Line 150: clusters 0,2, 20 are annotated as branchial and peribranchial epithelia, but they seem to overlap with TFs characteristic for the heart. What is the connection between these tissues?

Response: In the clusters mentioned by the reviewer (0, 2, and 20), the only differentially over-expressed transcription factors that could be considered “heart-associated” are *Gata-a* (i.e *Gata4/5/6*), *Tbx1/10* and *Nk4* (i.e. *nkx2-3/5/6*)**.** However, in ascidians these genes are not exclusively cardiac markers. Indeed, they are key regulators of the cardiopharyngeal program but are also involved in pharyngeal tissue

specification (Wang et al., 2013, *PLoS Biology*) and formation of the gut (Ricci et al. 2016 *Dev. Biol.*; Supp. Talbe 3), which is consistent with our annotation of these clusters as branchial and peribranchial epithelia**.**

Moreover, Nk4 expression has previously been reported during the early stages of budding in both *Botryllus schlosseri* and *Polyandrocarpa zorritensis* (Ricci et al., 2016 *Scientific Rep.*; Alié et al., 2018 *Mol. Biol. Evol.*). Notably, cluster 2 also contains budlet cells, suggesting that the expression of this transcription factor may reflect the activation of developmental programs associated with budding rather than a direct cardiac identity.

Given the well-known pleiotropic roles of these regulators outside the ascidian cardiopharyngeal network (summarized in Supplementary Fig. 7), their presence in these epithelial clusters is therefore not unexpected. In addition, the origin of the heart during asexual budding in colonial ascidians remains unresolved (Prünster et al. 2019, *EvoDevo*; Nunzi et al. 1979, *Dev. Biol*). Consequently, the possibility that a subset of epithelial cells within the branchial/peribranchial region may retain multipotent cardiopharyngeal potential cannot be excluded.

To clarify this point, we have expanded the session Results (Page 9, lines 8-12) to explicitly address this issue and to highlight the possible developmental relationship between these epithelial territories and heart formation during budding.

Reviewer #3:

This manuscript uses single-cell sequencing approaches to probe the cellular and gene regulatory basis of bud emergence in the tunicate Botryllus schlosseri. As one of the intriguing features of the system, individuals undergo asexual reproduction via budding of new individuals (zooids) from adult animals. This process raises a number of interesting questions in terms of cell and evolutionary biology. As nicely explained in the introduction, a long-standing question in the field concerns the origin of the bud founder cells, with circulating pluripotent stem cells as one option and somatic epithelial cells as another. Building largely on the analysis of two extensive single-cell sequencing data sets, the authors provide further evidence for the epithelial origin of the zooid.

Overall, the manuscript is well written and the analyses are generally sound (see technical questions below). The first part of the manuscript is essentially a cell-atlas-style description of cell types in adults and emerging zooids in B. schlosseri, combining scRNA-seq data with a seemingly new single-channel HCR-based protocol to localize the corresponding cell types within the animal. The analysis of the bud founder cells in Figures 6 and 7 combines lineage analyses based on the single-cell data sets with HCR localizations. By the nature of the work, large sections of the manuscript are model-organism specific, but they could be made more accessible to non-tunicate researchers (see comments below). However, the origin of the zooid founder cells is of broad interest to the cell and developmental biology community. With the addition of orthogonal evidence further corroborating the sequencing data, I can recommend publication of this manuscript in Nature Communications.

Response: We thank the reviewer for the positive evaluation of our work and for the constructive suggestion to incorporate additional orthogonal evidence supporting the conclusions drawn from the single-cell analyses. We address these points in detail below and indicate where the manuscript has been revised accordingly.

Major points

1) For non-specialists, the expression data are often hard to interpret. This is because many of the sections are not annotated and appear to be cut and oriented in different planes (Fig. 2, Fig. 3c). –– cartoon and clear indication of the tissues represented in specific sections would greatly improve the accessibility of these data.

Response: to improve the accessibility of the expression data for non-specialist readers Figures 2, 3C, 5H-I, 6A and 6H, as well as Supp. Figures 8B-D, 10A, 13A, 14D-E, 15F, 17, 18 and 19 have been revised to include section coordinates, developmental stage, anatomical outlines, and key structural annotations to facilitate interpretation. General anatomy explainer cartoons have been also added in Figure 2.

2) The lineage reconstructions are certainly interesting and, in combination with the de-differentiation data, are supportive of an epithelial origin of the zooid founder cells. However, given the caveats of scRNA-seq data, especially for detecting rare events, orthogonal lines of evidence on this important point would be very valuable. If morpholino or RNAi approaches remain difficult in this system, whole-mount in situ hybridizations with the new markers might help to delineate the earliest events in zooid formation. Alternatively, could maybe lineage tracing based on SNPs in the scRNA-seq data help corroborate the somatic/epithelial origin of the zooids?

Response: To provide additional evidence, we expanded the spatial validation of candidate markers using whole-mount HCR and adapted double-HCR technique to colonial tunicates. We specifically focused on early budding stages (budlet stage D), prior to the clear cytological manifestation of the bud onset (budlets stages A to C), leveraging the temporal resolution of the scRNA-seq dataset.

- More specifically, in Figure 6H, we added three further founder-specific markers (*Boschl.Bs3.g4311*, *Boschl.Bs3.g4316*, and *Boschl.Bs7.g100032*), selected among the top differentially expressed in this cell population, that further refine the spatial localization of the founder population (Page 23, lines 3-4; Figure 6H and figure legend).
- We also adapted a double-HCR protocol and demonstrates the spatial overlap of the identified founder markers within the same cell population (Page 23, lines 6-7; Supp. Figure 18A).
- In addition, we did add double-HCR experiments, combining epithelial markers (*Boschl.Bs16.g20741=COL2A1*, *Boschl.Bs16.g20739-PAX5.1*) with founder-associated genes (*Boschl.Bs3.g4311*, *Boschl.Bs14.g18091-MBNL1*, *Boschl.Bs14.g18091-MBNL1*, *Boschl.Bs6.g8341-NKX2-3*), and provided improved resolution of the transition from peribranchial epithelium to budding founder cells (Page 23, lines 14-18; Figure 7B and Supp. Figure 19).

As pointed out by the Reviewer functional perturbation approaches such as morpholino delivery are currently difficult and not feasible in this system, as these tools are typically applied during embryogenesis, whereas budding occurs later and reuses many genes that are also required during early development. Perturbing these genes at the embryonic stage would therefore prevent normal development and preclude analysis of their potential role in budding. RNAi approaches are also not yet reliably established in Botryllus (ongoing work is attempting to optimize this technique). Also, lineage reconstruction based on SNP information from the scRNA-seq data is unfortunately not feasible in our dataset, as samples consist of pools of tens to hundreds of buds (cf. Supplemental Table 1) at comparable developmental stages. While they share a common base genotype as they derive from a founding individual, this mix of many buds in each sample prevents making SNP-based lineage inference.

3) The cell counts reported in Supplementary Table 2 suggest that sample ML245-D exceeded 20,000 cells, which is curious given the technical limitations of the 10x Chromium kit. Can you please explain the origin of the high cell count in this case?

We have added barcode-rank plots for all samples in Supplementary Figure 3 and modified the figure legend (Pages 52-53). These plots show a clear and well-defined inflection ("knee") for ML245-D at ~20,000 barcodes, supporting that the higher cell count reflects genuine cell-associated barcodes rather than ambient RNA contamination or an ambiguous threshold.

The higher cell number in ML245-D originates from variability in cell loading. Although we targeted ~10,000 cells per sample based on pre-loading concentration estimates (see Methods), the 10x Chromium Single Cell 3' v3 kit allows higher loading densities, with the expected trade-off of increased multiplet rates. Consistent with this, doublet detection using scDblFinder identified a higher proportion of doublets in ML245-D (13.9%) compared to the other samples (7.6–11.1%), indicating that the increased recovery is attributable to higher input rather than technical artefacts. We update these information in Supplementary figure 3B (Page 52).

Similarly, the need to remove apparent low-quality clusters during cluster subsetting is somewhat unusual and may point to issues with the initial cell-filtering cutoffs. Please comment and include barcode-rank plots of the data in Supplementary Table 2 to allow a better appreciation of the data quality across samples.

Response: This approach is motivated by the observation that low-quality cells were not uniformly distributed but were largely confined to specific clusters (e.g., cluster 12; Supplementary Figure 4), which shows a bimodal distribution of gene counts. Given the diversity of cell types present in our dataset, setting a harsher threshold from the start would have led to removing the majority of cells in other clusters (notably clusters 5, 8, 20, 21, 26, 30 and 33). Post hoc filtering within subsets therefore allowed removal of low-quality cells while preserving rare or transcriptionally less complex populations that would otherwise have been lost under a global threshold (see Material and Methods, page 35 lines 29-30 and page 36, line 16).

To improve transparency, we now:

(i) included, as suggested, barcode-rank plots for all samples (Supplementary Figure 3A, pages 52-53),

(ii) report per-sample doublet rates (Supplementary figure 3B, pages 52-53),

(iii) clarified the rationale and implementation of the two-step filtering procedure in the Methods section (Page 35, lines 8-15; page 35 lines 29-30 and page 36, line 16) .

Minor points

In Supplementary Figure 1B, all three flow cytometry panels are labelled as ML208, whereas the legend states that the figure corresponds to ML231-B2. Please clarify this inconsistency.

Response: They are the same sample. The nomenclature just corresponds to different points of the protocol (ML208 as dissociated cells versus ML231 as single cell encapsulation and sequencing). We modified the figure (Page 48).

In line 227, planarians should be added to the list of animals with adult pluripotent stem cells, as they were instrumental in defining this cell class.

Response: we added planarians as well as relative references (Page 14, line 6).

Response to Reviewers

Manuscript: "*Multi-stage single-cell atlas of a chordate non-embryonic development unveils dedifferentiating bud founder cells*"

(NCOMMS-25-87463A)

Reviewer #1

(Remarks to the Author): *I confirm my approval of the publication of the manuscript by Lebel et al. However, I regret to say that my previous suggestions for improvement were almost completely neglected. As I said in my previous revision, Fig. 1A is modified from Ballarin et al.,2008, but this was not modified in the text (now it appears from Wolf et al., 2019), neither was the citation present among the references*

Response: We sincerely apologize for this oversight. Following your previous review, we had indeed updated the attribution of Fig. 1A to Ballarin et al. (2008) as you suggested. Unfortunately, during the final stages of manuscript preparation, this correction was inadvertently lost, resulting in the incorrect attribution to Wolf et al. (2019) and the omission of the corresponding reference from the bibliography.

This has now been corrected in the revised version. Figure 1A is properly attributed to Ballarin et al. (2008), and the corresponding reference has been reinstated in the reference list. We thank the reviewer for pointing out this error.

Additionally, and more important, for sake of clarity, I suggested to replace the term "stage" with "phase" in most of the cases where the term appears. This suggestion was not taken into consideration but it is useful in order to avoid confusion. If it is correct to deal with the developmental stages of the zooid, from its appearance ad thickening of the peribranchial epithelium to the resorption at the takeover (11 stages according to Berrill (1941) and Sabbadin (1955); 12 stages according to Izzard (1973)), it is not correct to use the same term to indicate the cyclical changes in a colony. They should be called "phases" and each phase includes the developmental stages of zooids, buds and budlets. They are identified with four letters (A-D) by the Japanese and American researchers (and in this manuscript) and with a triplet of numbers referring to the developmental stages of the three colonial generations by the Italian scientists. As a consequence, the expressions "budlet stage D" or "bud stage A1" (page 12, line 6) should be replaced with "budlet (from colony) at phase D" or "bud (from colony) at phase A1".

I summarise here below the parts of the manuscript deserving changes:

Page 3, line 27. Here, the term "stage" is correct and should be maintained

Page 3, line 28. "Phase D" instead of "stage D"

Page 6, lines 4, 5,20. Replace "stage" with "phase" four times

Figure 2. "phase A1-2", "phase B1-2", "phase "C1-2"

Page 12, lines 6-7.

Page 17, line 27

Page 18, line 21

Page 21, lines 14. "A1-C2 budlet cells" should be modified to "budlet cells from phase A1-C2"

Page 21, lines 27. "A2-C2 stage budlet cells" should be modified to "budlet cells from phase A2-C2"

Page 23, lines 5, 16

Page 24, line 1

Page 26, lines 12, 16, 17, 18

Page 28, line 23

Page 29, line 11

Page 31, lines 27, 28
Page 32, lines 12, 13
Page 33, line 6
Page 34, line 9. Replace “A1 sample” and “B2 sample” with “sample from phase A1” and “sample from phase B2”
Suppl. Table 1. “Phase” instead of “stage” in the title of the2nd column
Suppl. Fig. 2A replace “A1 stage circulating cells” and “B2 stage circulating cells” with “circulating cells from phase A1 “ and “circulating cells form phase B2”
Suppl. Fig. 5A. “Budding dataset clusters, shown by phase”
Suppl. Fig. 5B. “replace “B2 stage samples” with “samples from phase B2”. In the title of the legend replece “stage” with “phase”. In the legend of the part B replace “B2 samples” with “samples from phase B2”
Page 75 (legend of suppl. Fig. 17), lines 2, 3, 6, 9, 15, 17
Page 77 (legend of suppl. Fig. 18), line 7. “A1-B2 budlet” should be replaced with “budlet from phase A1-B2”

Response: Following the reviewer's recommendations, we have now systematically revised the terminology throughout the manuscript, figures, supplementary figures, tables, and legends. Specifically, we now consistently use "phase" to refer to the cyclical states of the colony (A-D and A1-C2), while retaining "stage" exclusively for the developmental stages of individual zooids, buds, and budlets, in accordance with the established terminology proposed by Berrill, Sabbadin, and subsequent authors.

All of the specific corrections listed by the reviewer have been implemented in the revised manuscript. We believe this substantially improves the clarity and terminological consistency of the work, and we thank the reviewer for insisting on this important distinction.

Reviewer #2

(Remarks to the Author): *The authors carefully revised the manuscript and fully satisfied my and the other reviewers comments in my view. They prudently toned down the conclusions leaving room for alternative explanations and the limitations of the study. Taken together, this is a beautiful, and important piece of work. I recommend to accept it now.*
One last little thing:
Pg. 9. "are also involved pharyngeal development"
should be: are also involved in pharyngeal development

Response: We sincerely thank the reviewer for the very positive assessment of our work and for the constructive feedback throughout the review process. We greatly appreciate the recommendation for acceptance. The typographical error on page 9 has been corrected as suggested ("are also involved in pharyngeal development").

Reviewer #3:

Thank you- the revisions conclusively address all my concerns. I have no further concerns and congratulate the authors on a very nice piece of work.

Response: We sincerely thank the reviewer for the positive evaluation of our work and for the constructive comments throughout the review process. We greatly appreciate the recommendation for acceptance.
